# Integrated preservation of water activity as key to intensified chemoenzymatic synthesis of bio-based styrene derivatives

**DOI:** 10.1038/s42004-024-01138-x

**Published:** 2024-03-14

**Authors:** Philipp Petermeier, Jan Philipp Bittner, Tobias Jonsson, Pablo Domínguez de María, Emil Byström, Selin Kara

**Affiliations:** 1 Biocatalysis and Bioprocessing Group, Department of Biological and Chemical Engineering, Aarhus University, 8000 Aarhus C, Denmark; 2 Institute of Thermal Separation Processes, Hamburg University of Technology, 21073 Hamburg, Germany; 3 Diduco AB, Tvistevägen 48C, 90736 Umeå, Sweden; 4 Sustainable Momentum SL, Av. Ansite 3, 4-6, 35011 Las Palmas de Gran Canaria, Canary Islands Spain; 5 SpinChem AB, Tvistevägen 48C, 90736 Umeå, Sweden; 6 Institute of Technical Chemistry, Leibniz University Hannover, 30167 Hannover, Germany

**Keywords:** Biocatalysis, Synthetic chemistry methodology, Green chemistry, Immobilized enzymes

## Abstract

The valorization of lignin-derived feedstocks by catalytic means enables their defunctionalization and upgrading to valuable products. However, the development of productive, safe, and low-waste processes remains challenging. This paper explores the industrial potential of a chemoenzymatic reaction performing the decarboxylation of bio-based phenolic acids in wet cyclopentyl methyl ether (CPME) by immobilized phenolic acid decarboxylase from *Bacillus subtilis*, followed by a base-catalyzed acylation. Key-to-success is the continuous control of water activity, which fluctuates along the reaction progress, particularly at high substrate loadings (triggered by different hydrophilicities of substrate and product). A combination of experimentation, thermodynamic equilibrium calculations, and MD simulations revealed the change in water activity which guided the integration of water reservoirs and allowed process intensification of the previously limiting enzymatic step. With this, the highly concentrated sequential two-step cascade (400 g·L^–1^) achieves full conversions and affords products in less than 3 h. The chemical step is versatile, accepting different acyl donors, leading to a range of industrially sound products. Importantly, the finding that water activity changes in intensified processes is an academic insight that might explain other deactivations of enzymes when used in non-conventional media.

## Abstract (web summary)

Lignin-derived phenolic acids can be upgraded to styrene derivatives through chemoenzymatic processes, however, the scale-up of such processes remains challenging. Here, the authors find that controlling the water activity during the decarboxylation of bio-based phenolic acids, including through the integration of a water reservoir, enables high conversions and efficient reaction times, that can be combined with a versatile acyl donor substrate scope.

## Introduction

**Fig. 1:**
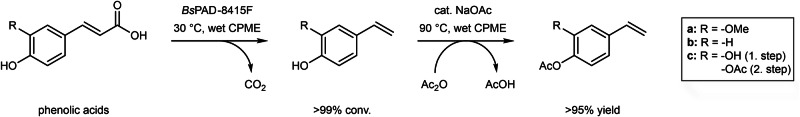
Chemoenzymatic synthesis of acetylated hydroxystyrenes from phenolic acids in water saturated (wet) CPME. This sequential one-pot two-step process relies on biocatalytic decarboxylation and chemical acetylation. The decarboxylation is catalyzed by *Bs*PAD, a phenolic acid decarboxylase from *Bacillus subtilis*, covalently immobilized on methacrylate resin ECR8415F and termed *Bs*PAD-8415F. The acetylation is base-catalyzed by sodium acetate. As illustrated by the unspecified residue R, the cascade accepts a range of phenolic acids depending on the substrate scope of the decarboxylase: ferulic acid (FA), *p*-coumaric acid (*p*CA), and caffeic acid (CA).

The use of bio-based feedstock´s represents a promising strategy to replace fossil-based resources in future chemical processes. Within so-called biorefineries, different biomasses (*e.g.*, lignocellulose, municipal organic wastes, *etc.*) can be smartly defunctionalized to deliver an array of chemicals and biofuels^1^. In the quest for such transformative chemistry, we recently reported a chemoenzymatic strategy to upgrade lignin-derived phenolic acids to styrene derivatives that can be used as bio-based polymer precursors (see Fig. 1)^2^. Earlier chemical routes have successfully demonstrated this valorization too, yet with shortcomings such as the severity of reaction conditions, the use of hazardous chemicals, or limitations in cost-effective upscaling due to type or number of operations^3,4^. Additionally, the range of disclosed well-controlled polymerizations of hydroxystyrene-type monomers underpins the potential role that these renewable polystyrenes may eventually play^3–9^. Thus, by combining experimental and computational means, we previously established a sequential chemoenzymatic cascade which leverages the high catalytic activity of phenolic acid decarboxylase from *Bacillus subtilis* (*Bs*PAD, PDB entry: 2P8G) under mild reaction conditions^2^. Moreover, this preceding study revealed that covalent enzyme immobilization on a macroporous polymeric resin (ECR8415F) resulted in heterogeneous biocatalyst beads (*Bs*PAD-8415F) with increased enzymatic stability and reusability^2^. By pairing this biocatalyst with a cost-effective inorganic base catalyst in a micro-aqueous reaction system based on the (potentially) renewable solvent cyclopentyl methyl ether (CPME)^10^, a robust chemoenzymatic synthetic routine was established (Fig. 1). Herein, robustness refers to the beneficial absence of enzyme leaching, the high degree of retained biocatalyst activity enabling catalyst recycling, and the easily scalable nature of the synthetic process^2^. Importantly, the same solvent was applied in both catalytic steps, leading to reduced waste formation, and simplifying the linear sequential two-step cascade^11^. The overall synthesis was demonstrated for the conversion of ferulic acid (FA) into 4-acetoxy-3-methoxystyrene (AMS) on a 1 L scale with a product titre of 19 g·L^–1^ (0.1 M) and an isolated yield of 18.3 g AMS. Although both the scale-up and preparative implementation were successful, the former volumetric productivities (1.6 g·L^–1^·h^–1^) and product titres remained insufficient to envisage an on-scale synthesis of potential high-volume chemicals.

Here, two aspects were considered crucial to demonstrate that the presented chemoenzymatic transformation could attract future industrial interest. On the one hand, yields and volumetric productivities of the enzymatic step must be significantly improved. However, in preliminary experiments, the reaction did not proceed further when higher substrate loadings were added. On the other hand, broadening the chemical step’s versatility is valuable at the industrial level since an ample range of structurally related products would be accessible by the same reaction system. This work addresses both research lines, showing that the reported chemoenzymatic strategy can reach industrially sound conditions.

## Results and Discussion

### Understanding intensified biocatalysis

**Fig. 2:**
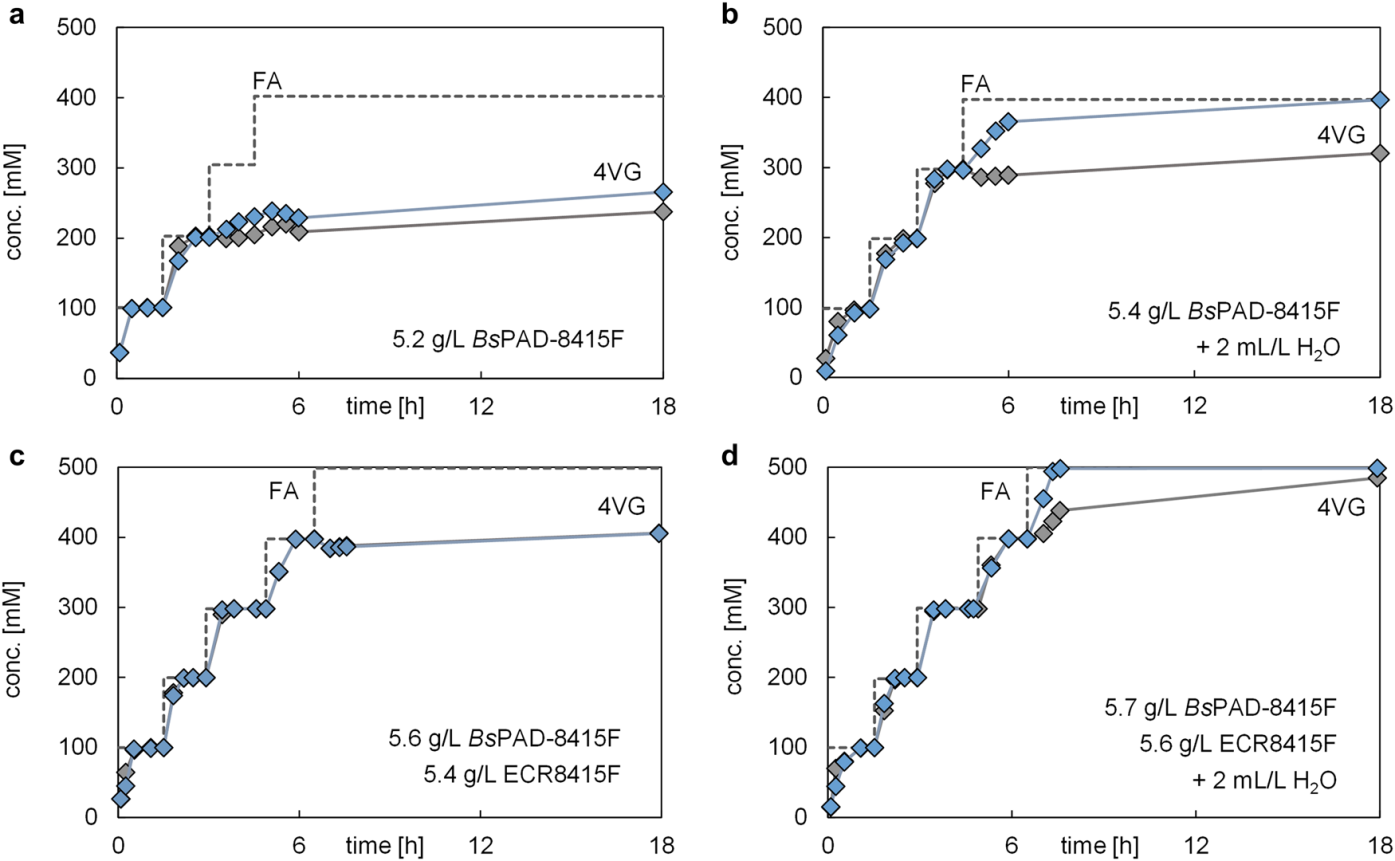
Fed-batch process intensification using different water reservoirs to sustain water activity during the reaction progress. The dashed line indicates the total amount of added substrate ferulic acid (FA), and the two respective other progress curves indicate the accumulation of the decarboxylation product in each experimental duplicate. Common reaction conditions: 100 mM FA (initially), wet CPME, 30 °C, 1000 rpm, 1 mL reaction volume. Specifics regarding the average use of immobilized enzyme (*Bs*PAD-8415F), untreated moist support beads (ECR8415F), and added water are given in graphs (**a**–**d**) and represent experiments using (**a**) biocatalyst beads, (**b**) biocatalyst beads plus excess water, (**c**) biocatalyst beads plus moist support beads, and (**c**) a combination of biocatalyst beads, moist support beads, and excess water. All immobilized enzyme beads, moist support beads, and water were added just once at the beginning of fed-batch experiments; only FA was added stepwise.

A critical step to reach industrially sound conditions is the intensification of the cascade. For the proof-of-concept, all reactions were performed at modest product titres (100 mM). However, thermodynamic equilibrium calculations from our previous work indicate much higher solubilities of hydroxystyrenes in wet CPME^2^. Hence, in the first set of experiments, a fed-batch process was established (Fig. 2). Based on the previously established decarboxylation (100 mM FA, 5 g·L^−1^ *Bs*PAD-8415F beads, wet CPME, 30 °C, 1000 rpm), the fed-batch included the addition of more solid substrate FA (corresponding to +100 mM when dissolved) once the first batch was fully converted. Yet, while the first batch always proceeded as expected^2^, the enzymatic activity ceased immediately as the second batch of substrate was added. Interestingly, the subsequent addition of more biocatalyst (*Bs*PAD-8415F) led to continued decarboxylation, eliminating product inhibition as the potential root cause. Quite strikingly, when a particularly freshly prepared *Bs*PAD-8415F biocatalyst lot was used, fed-batch experiments could be run successfully, starting by converting 100 mM FA, subsequently continuing to convert another 100 mM FA, and only gradually stopping after another feeding of 100 mM FA. Overall, a total of 252 mM FA was successfully converted by using the freshly prepared biocatalyst (Fig. 2a).

As mentioned above, limitations due to product inhibition were ruled out based on experimental observations, albeit known to be a common phenomenon for PAD:s in aqueous buffer systems^12–14^. Therefore, the transition from aqueous to non-conventional media plays a role on the solvation of the inhibitory product within the microenvironment of the enzyme. In fact, solubilities of accumulating hydroxystyrenes in wet CPME are extremely high ( > 1 kg·kg^−1^), whereas more limited values are noted in polar aqueous buffer systems (range of some g·kg^−1^). By envisioning the enzyme as a pseudo-phase that is effectively distributed in the bulk phase of the reaction medium, wet CPME might act as an effective product sink, draining the local hydroxystyrene concentration from the enzyme, opposite to what would occur in the buffer. In aqueous media, the more hydrophobic sites of the enzyme would become the thermodynamically more favourable place for hydroxystyrenes to reside. This would force the product to interfere in the reaction progress, leading to product inhibition. Likewise, substrate inhibition can also be excluded as the cause of the sudden loss of enzyme activity during substrate addition. This is substantiated by the fact that at every stage of substrate feeding a maximum of 100 mM FA was added, which was the same substrate concentration present in the highly productive initial batch.

To explain the observed results, the possibility that the substrate dissolution might cause a sudden but transient disruption in the reaction medium affecting the biocatalyst was considered. To assess this, the heterogeneous biocatalyst *Bs*PAD-8415F was compartmentalized in a rotating bed reactor (RBR) from SpinChem AB (Umeå, Sweden). After full conversion of the initial 100 mM FA, the RBR holding all the heterogeneous biocatalyst was taken out of the reaction mixture and stored in a beaker of wet CPME, while the reaction mixture was fed with an additional 100 mM FA. Only after full dissolution of the new added substrate, the RBR including the biocatalyst was returned to the reaction mixture. In this experiment, a severe catalyst deactivation was observed as well, dismissing the idea of a transient detrimental effect during the dissolution process.

As another hypothesis, we considered that a cooperative action of the substrate (ferulic acid, FA) and hydroxystyrene product (4-vinylguaiacol, 4VG) at increased concentrations may generate changes in the media (or the enzyme) that could lead to the observed deactivation. This effect might not have visible consequences at diluted substrate concentrations, but would be reflected in intensified systems (higher substrate or product loadings). Considering that so far only fresh *Bs*PAD-8415F preparations enabled a successful fed-batch operation, identifying their advantage was critical. Interestingly, with the standard kinetic assay in buffer, no significant difference in specific activity between freshly prepared and stored biocatalyst preparations was observed (always 198 ± 27 U/g). However, their slightly different pourability during handling and weighing indicated variation in moisture contents. This can be attributed to the loss of water content from the immobilized enzyme preparations during their storage. Since it was shown previously that the enzyme activity is highly sensitive to changes in the water content in CPME^2^, this factor was assessed further. In our earlier account, we reported that the highest enzyme activity in CPME was observed at 100% water saturation, which also corresponds to a water activity ${a}_{W}$ = 1.0 (100% relative humidity). Water activity has been recognized for decades as an important parameter to correlate with enzyme activity, from classic organic media to neoteric solvents^15–19^. This arises from the fact that enzyme molecular flexibility and thus catalytic activity typically require structurally bound water, necessitating a minimum water activity in the enzyme’s microenvironment.

While the effects on enzyme activity created by different water activities may not be so significant at low substrate concentrations – where water activity would remain relatively stable –, this may be different at intensified systems (*e.g.*, fed-batch operations), where the water activity would not remain sufficiently constant. If this were the case, an extra provision of water should sustain higher fed-batch conversions at higher substrate loadings, as presumably observed with fresher *Bs*PAD-8415F preparations (which contained more water). To validate this, we spiked the already water saturated micro-aqueous reaction system with small amounts of additional (free) water (Fig. 2b). Furthermore, unmodified commercial support ECR8415F beads were also thought of as potentially useful water reservoirs, as they were specified with a moisture content of 70–80% and had the advantage of not introducing a second liquid phase to the system (Fig. 2c). Additionally, a combination of ECR8415F beads and extra free water was also tested in the fed-batch experiments (Fig. 2d). The results clearly provide strong evidence for the importance of changes in water activity and the benefit of using auxiliary water reservoirs during the reaction progress. Thus, while fresh biocatalyst beads without water addition yielded a maximum of 252 mM 4VG (Fig. 2a), the addition of extra free water led to 359 mM conversion (Fig. 2b). An even better result of 406 mM 4VG was enabled by the concomitant use of unmodified ECR8415F beads (Fig. 2c). Notably, the highest product titre of 492 mM 4VG was obtained when the addition of free water and ECR8415F beads were combined (Fig. 2d).

**Fig. 3:**
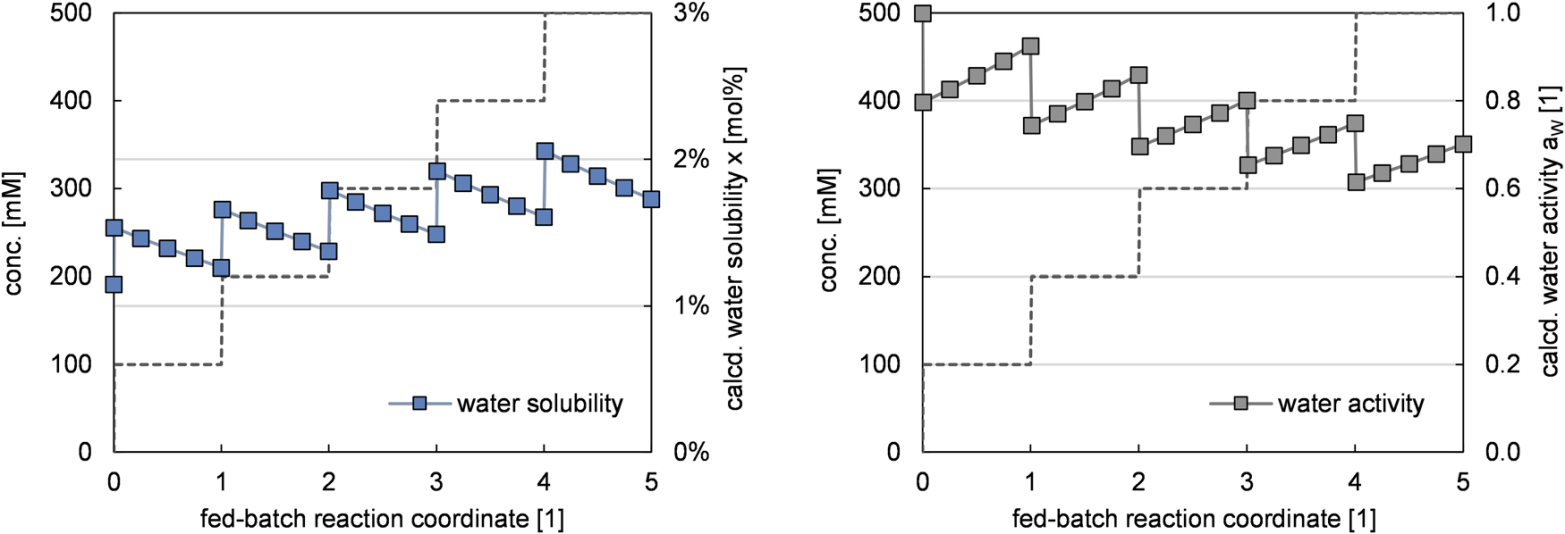
Calculated water solubility [mol%] and water activity [1] in a hypothetical fed-batch reaction. These thermodynamic equilibrium calculations assumed only initially dissolved water, but no added free water (reservoir). The reaction coordinate refers to individual batches of added and gradually consumed substrate in 100 mM intervals. Dashed lines serve as visual aids and indicate the total process intensity, *i.e.*, cumulative concentration of substrate and product. All solubility and activity data were calculated using BIOVIA COSMOtherm 2020^20–23^.

To further shed light on these findings, thermodynamic equilibrium calculations of the reaction medium were applied to characterize its water solubility and water activity during fed-batch operations. To that end, the BIOVIA COSMOtherm 2020 software package^20–23^ was used to assess mixtures of phenolic acid substrate (FA), hydroxystyrene product (4VG), and reaction media (CPME), that represent distinct stages in the fed-batch experiment (Fig. 3). The tool employed, COSMOtherm, is a predictive thermodynamic model that allows to estimate solubilities, phase equilibria, and chemical activities in bulk phases.

From Fig. 3, some conclusions can be drawn. First, the changing composition of the overall reaction medium during the evolution of the reaction has inverse effects on water solubility and water activity. Second, it becomes clear that the addition of the substrate (FA) is predicted to have the most severe influence: being more hydrophilic, it causes both an immediate increase in water solubility and reduces water activity. Third, between each substrate addition, FA is gradually converted to 4VG, which is less hydrophilic by nature, and causes lower water solubility and increased water activity. Overall, this refined picture is consistent with the chemistry maxim *‘Similia similibus solvuntur’*: The overall reaction medium is composed of the following increasingly polar constituents CPME, 4VG, FA, and H_2_O. Any action that increases the overall polarity by lowering the proportion of apolar constituents – that is, when adding fresh substrate – enhances the reaction medium’s capacity to solubilize polar compounds, and vice versa. In fact, the effect of co-solubilized water on the solubility of FA in CPME is an example of this behaviour, as it showed that saturating the organic solvent with water increased FA solubility from 17 to 31 g·L^−1^ ^2^.

From a practical perspective, the trend observed in Fig. 3 reveals three distinct facts: (i) as both substrate and product are more polar than the solvent, an increase in their combined concentration [P + S] necessarily increases overall polarity and gives an overarching upwards trend for water solubility; (ii) between the substrate and the product, the former is more polar (due to its carboxylic acid moiety), rending its conversion to the product a net loss in overall polarity, explaining the downwards trend in water solubility between each substrate feeding step; (iii) the water solubility can be understood as the capacity to which the reaction medium can take up water. Once water solubility exceeds the amount of water available, water activity decreases in inverse proportion. Thus, these findings explain why enzyme deactivation was consistently triggered by substrate feeding, and even more importantly, how such deactivation can be avoided to reach industrially sound conversions in intensified biocatalytic processes. Moreover, the calculations for the hypothetical fed-batch in Fig. 3 suggest that already the first dissolution of 100 mM FA in water saturated CPME drops the water activity by a significant 20%. Thus, the internal moisture content of the enzyme beads may be essential to counter the initial drop in water activity in the so far uncritically viewed first batch.

**Fig. 4:**
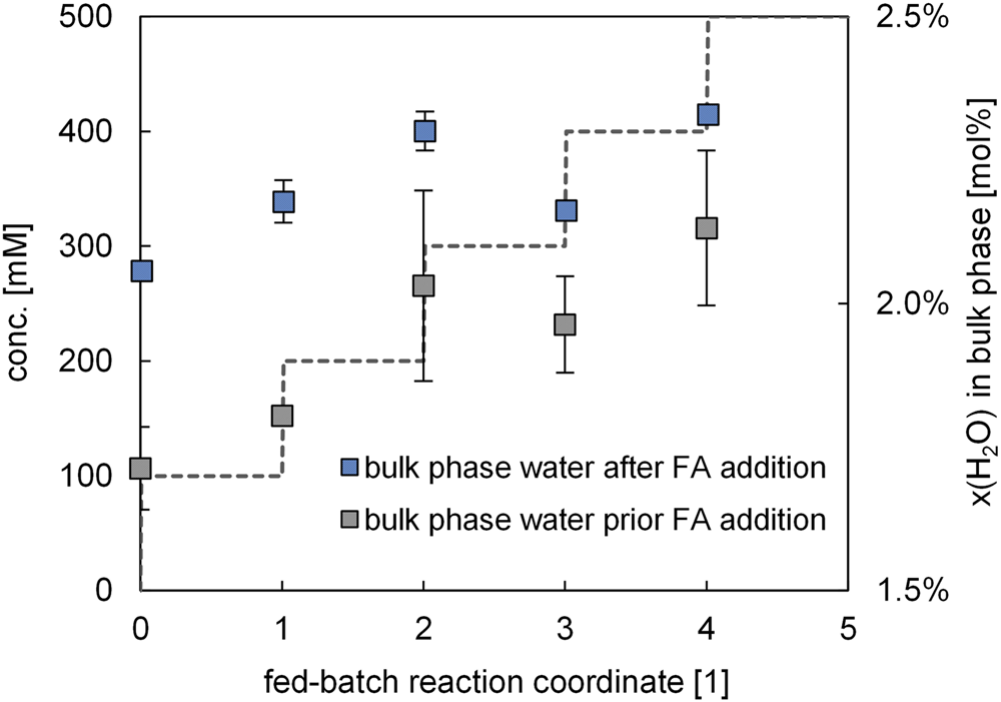
MD simulated dissolved water in the bulk organic solvent phase during fed-batch decarboxylation of ferulic acid in wet CPME. Any increase of dissolved water content is due to relocation of water from the enzyme into the bulk organic phase. The dashed line serves as a visual aid and indicates the total amount of substrate fed. Results are based on simulation duplicates from independent initial configurations.

Apart from computationally assessing variations in the reaction medium along the fed-batch reaction progress, in the next step it was studied how these changes may translate to the enzyme. For this, it is assumed that any changes in the reaction medium are in direct thermodynamic equilibrium with the pseudo-phase of the enzyme, meaning that if water activity drops in the reaction medium, the same is to be expected to occur at the enzyme. In other words, as the water solubility of the reaction medium increases, its capacity to compete with the enzyme for available water increases, leading to the gradual stripping of water from the enzyme. To validate this, molecular dynamics (MD) simulations were performed, in which first the enzyme was equilibrated in water-saturated CPME, before again substrate and decarboxylation product were added in a way that the simulations represent the stepwise changes in the fed-batch process. To keep the complexity of the modelled system manageable, the biocatalytic system was approximated by the free enzyme and did not include the carrier. The carrier was seen as another pseudo-phase with constant properties and was thus purposely dismissed in these trend simulations. The output of these simulations was the molar concentration [mol%] of dissolved water in the organic bulk phase, as to see whether in a dynamic system water would indeed be withdrawn from the enzyme. The results are given in Fig. 4, and strongly substantiate this idea, as the water concentration in the organic phase continuously rises along the reaction progress. To further quantitatively elucidate the exact mechanism(s) of associated enzymatic activity loss (structural distortions, degradation, loss of mobility, *etc.*), much larger computational studies would be required.

Finally, it must be noted that due to computational logistics, the enzyme-to-reaction-medium ratio in these simulations was much higher than in a realistic reaction system. This means, that the stripping effects that draw water from the enzyme are expected to be even more pronounced in a real-world setup, further emphasizing the importance of the above conclusions. Altogether, these findings highlight that it is not sufficient to ensure optimal reaction conditions (*e.g.*, optimum water activity) at the beginning of the reaction. The chemical evolution of the overall system along the reaction progress must be considered as well. This is of particular importance in the case of concentrated mixtures (*e.g.*, reactions for industrial purposes), where substrates and products significantly affect the chemical composition of the bulk phase. There, it is important to understand the impact of all species to design a reactive system in which the operational windows of all critical parameters are abided by at all stages. In more general terms, the changes in the water activity during intensified reactions may have implications not only on this cascade process, but also in other enzymatic systems with high substrate loadings that may show impairment in hydrophilicity between the substrate and the product.

As shown, the co-solubilization of H_2_O in CPME increases the initial substrate solubility of FA. Similarly, the accumulation of the product 4VG also enhances the overall substrate solubility of FA, and both increasing concentrations of FA and 4VG cause higher solubility of H_2_O. Consequently, the whole reactive system becomes harmoniously self-intensifying, provided that there are sufficient water reservoirs to maintain the enzymatic activity.

### Chemoenzymatic cascade intensification: The all-in strategy

Once enzyme deactivation by subsequent FA additions could be explained (empirically and theoretically), the set-up of an intensified catalytic cascade was considered. To that end, an all-in strategy was envisioned, starting with larger, initially insoluble amounts of FA in wet CPME ( > 150 mM). Compared to the previous fed-batch operation, this all-in strategy consists of three main stages:

(i) Initiation shock: Excess amounts of FA are added to water-saturated CPME (${a}_{W}$ = 1), causing saturation of the homogeneous liquid phase with substrate and increasing water solubility in CPME. As this would reduce water activity to a suboptimal level (${a}_{W}$ < 1), additional free water reservoirs or sufficient inherent moisture content within the deployed enzyme beads are necessary to cushion the initial shock in water activity.
(ii) Concurrent evolution: The heterogeneous biocatalyst converts the substrate dissolved in the liquid phase. The product 4VG accumulates in the liquid bulk phase, causing the dissolution of more substrate. As a result, the proportion of polar species (FA and 4VG) in the liquid bulk phase grows disproportionally to the reaction progress, increasing overall polarity and water solubility. During this stage, a maximum amount of water is drawn from the water reservoirs to maintain water activity in the organic bulk phase.
(iii) Homogeneous finish: At the latest when all substrate has been dissolved, the zenith of water solubility has passed, as any further conversion reduces overall bulk phase polarity and thus water solubility. Hence, in this stage, it is expected that water reservoirs display a net-uptake of water.

Eventually, this should result in a homogeneous water-saturated solution of 4VG in CPME, as thermodynamic equilibrium calculations predict extremely high solubilities for hydroxystyrene intermediates in CPME (>1 kg·kg^−1^)^2^. This all-in strategy was tested at increasingly greater substrate concentrations (Table 1). The specific catalyst productivity [mmol_product_·g_cat_^–1^·h^–1^] was introduced as critical parameter for the systematic optimization. As it is clear from the first two entries (0.1 M), productivity suffers severely from the addition of free water. This is explained by the aggregation of catalyst beads in the small, undissolved water droplets (for >2 µL H_2_O added), which adds severe mass transfer limitations to the reactive system. However, by increasing the bead-to-free-water ratio, the proportion of catalyst beads affected by aggregation decreases and ultimately, the beads themselves provide enough water uptake capabilities to the system, eliminating the aggregation. This is reflected by the recovery of specific productivity in later entries in Table 1.

**Table 1:** Intensification study for the cascade transformation of ferulic acid (FA) to 4-acetoxy-3-methoxystyrene (AMS)

substrate [mol·L^–1^]	*Bs*PAD-8415F [g·L^–1^]	ECR8415F [g·L^–1^]	free H_2_O [mL·L^–1^]	decarb. conv. [%]	rxn. time [h]	productivity [mmol·g_cat_^–1^·h^–1^]	acetyl. conv. [%]	rxn. time [h]
0.1	5	0	0	>99	0.7	28.4 ± 1.5	97	2.0
0.1	5	0	4	>99	2.0	9.7 ± 0.3	96	2.0
0.3	10	0	4	>99	1.5	19.1 ± 1.0	>99	0.8
0.3	10	11	4	>99	1.1	25.5 ± 1.8	>99	0.8
0.5	11	12	4	>99	1.8	24.8 ± 0.1	>99	0.5
0.5	11	17	4	>99	1.8	25.5 ± 0.1	>99	0.4
1.0	10	18	4	>99	4.1	23.6 ± 0.5	>99	0.2
1.0	21	17	8	>99	1.7	27.1 ± 0.8	>99	0.2
1.5	31	20	10	>99	2.0	24.0 ± 0.3	>99	0.2
2.0	40	30	14	>99	2.0	24.8 ± 0.3	>99	0.1

According to the all-in strategy, not only all the biocatalyst beads (*Bs*PAD-8415F) and all the moist support beads (ECR8415F) but also all of the substrate was added from the beginning. Common reaction conditions: 1 mL water-saturated CPME, 30 °C, 1000 rpm; for the subsequent acetylation, the supernatants were subjected to +2 eq. Ac_2_O, +0.05 eq. Na_2_CO_3_, 90 °C, 1000 rpm. Conversions are based on relative product peak areas at 254 nm. The reported reaction times [h] and productivities [mmol_product_·g_cat_^–1^·h^–1^] are based on sampling times closest in time to given conversions. All results are based on experimental duplicates.

To decouple the use of biocatalyst beads from the required amounts of added free water, untreated carrier beads – without enzymes – ECR8415F were introduced again. As stated above, these beads with a moisture content of 70-80% have the advantage that they are of the same material as the catalyst beads, thus limiting the risk of abrasion. For all further experiments a minimum bead-to-free-water ratio of 4:1 (g:mL) was kept to ensure the highest catalytic productivity. This resulted in outstanding stable specific productivities and full conversions for up to 2.0 M substrate in 2 h reaction time. At these concentrations the initial mixture is a dense slurry and the reaction itself becomes very vigorous through CO_2_ release (decarboxylation step), complicating further advances in process intensification. In any case, given these results it becomes clear that the reaction system in CPME is only limited by the fluctuating water activity along reaction evolution, and does not show any significant product or substrate inhibition, not even at concentrations of 2.0 M (~400 g·L^−1^) substrate, which are definitely in the range of industrial set-ups. Furthermore, to telescope the intensified biocatalytic decarboxylation to the subsequent acylation, the obtained supernatants of various 4VG concentrations in wet CPME were subjected to our standard acetylation conditions^2^. Gratifyingly, full conversions were obtained in all intensified batches in rather low reaction times (<< 1 h) (Table 1). Moreover, higher concentrations are both beneficial for overall conversion as well as for the required reaction time.

Overall, this demonstrates the utility of our integrated measures to maintain water activity in organic media during biocatalytic conversion. It allows fully self-sustained reaction evolution and spares the need for sophisticated active control measures. Moreover, depending on the water sensitivity of the subsequent reaction, the water saturated organic solution of the biocatalysis product can either be directly subjected to the next reaction or can be treated with desiccant beforehand. Conveniently, in this case, no drying step is necessary which results in a straightforward, intensified chemoenzymatic cascade. The fact that the same reaction media can be directly transferred from one step to the next one simplifies the downstream significantly, reduces inherent costs, and leads to a diminished waste formation because the same solvent is reused in the entire pipeline. Considering the constant specific biocatalytic productivity and the increase in volumetric productivity of the chemocatalytic step at increased concentrations, we expect this cascade to not just be more resource-efficient but also more economically favourable at greater process intensity, *i.e*., at high product concentrations and high biocatalyst loadings.

### Process modularity: broadening the product scope

Once the entire reaction cascade was successfully established at intensified conditions with excellent conversions, attempts to broaden the applicability of the second step – the base-catalyzed acylation – were made. For this, acyl donors like vinyl acetate, isopropenyl acetate, acetic anhydride, and acyl chlorides were combined with different base-catalysts and assessed in the acylation of 4-vinylguaiacol (4VG). Long-chain fatty acid anhydrides were initially excluded due to low atom economy and the more cumbersome downstream caused by their amphiphilic surfactant-like properties. This screening corroborated that the combination of carboxylic anhydrides with inorganic base catalysts remained the most effective way to telescope the upstream biocatalytic conversion into an effective chemoenzymatic cascade, and no competitive reagent/catalyst pairing could be identified as a feasible alternative (Supplementary Discussion, Table S1). At the same time, however, a certain flexibility was observed with respect to the acyl acceptor (*cf*. Supplementary Fig. S1) as well as within the identified classes of acyl donor and catalyst, *i.e*., allowing the use of different carboxylic anhydrides and inorganic base salts depending on availability, cost, and intended acyl-decoration.

**Fig. 5:**
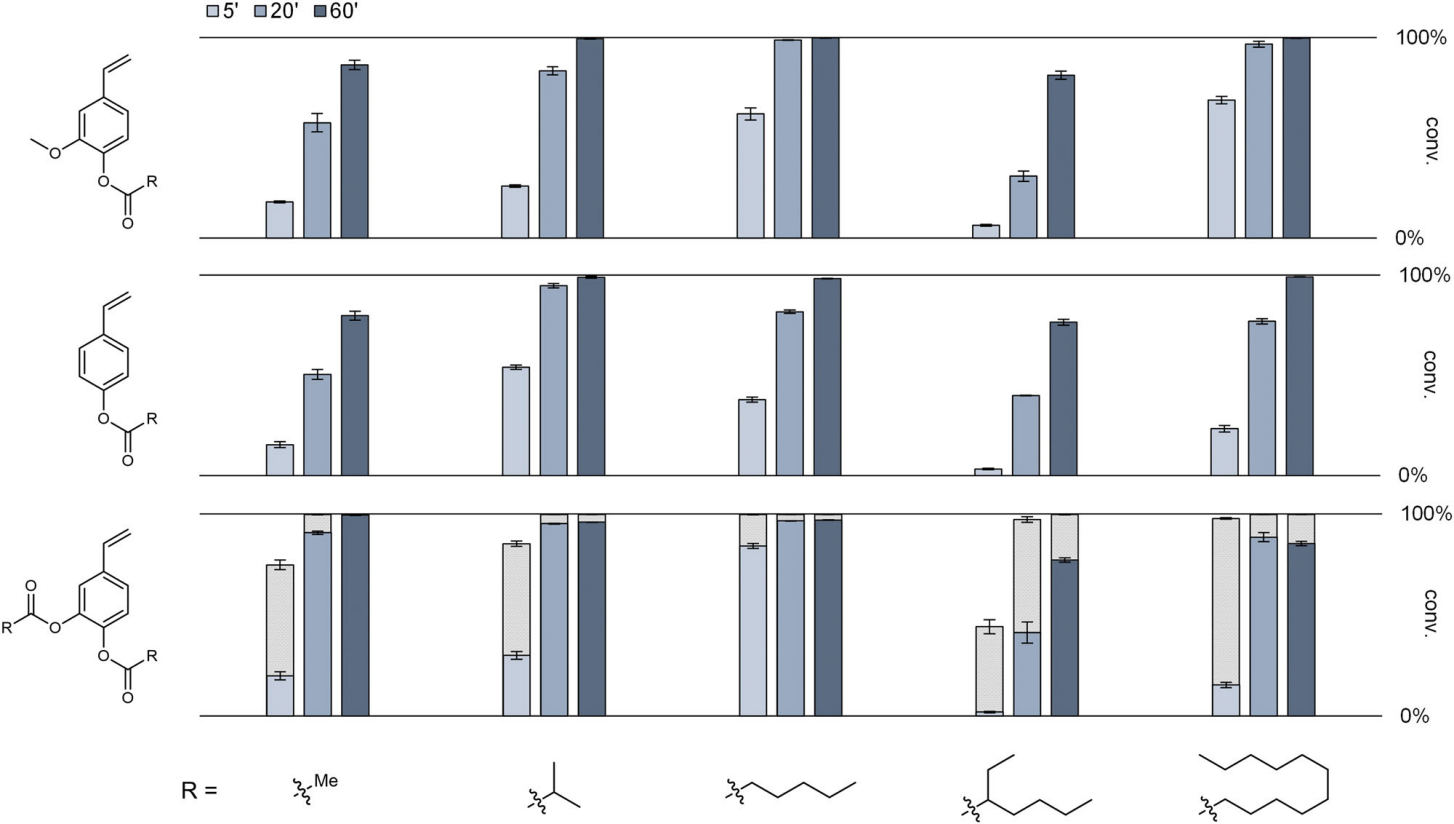
Product scope screening using carboxylic anhydrides as acyl donors. Conversions of unprotected to acyl-protected hydroxystyrenes are given for reaction times of 5, 20, and 60 min at standard reaction conditions: 100 mM hydroxystyrene, wet CPME, 0.05 eq. NaOAc, 2 eq. carboxylic anhydride (per phenolic-OH group), 90 °C, 1000 rpm. In the bottom row, grey bars represent the conversion to mono-acylated products, whereas blue bars show the formation of the diacylated product. All results are based on experimental duplicates and relative peak areas (HPLC, 254 nm). This screening necessitated highly versatile HPLC analytics to cover the entire range of polarities represented by all these products. Thus, we developed a single, moderately long (22 min) gradient method that enables chromatographic separation of all products from their respective substrates and intermediates (see Methods section and Supplementary Fig. S7).

On that basis, to broaden the applicability of the process even further, the one-pot two-step cascade was conducted with three substrates (FA, *p*CA, CA) and different anhydride acyl donors of varying length and branching profile: acetic anhydride (Ac_2_O), *iso-*butyric anhydride, hexanoic anhydride, 2-ethylhexanoic anhydride, and lauric anhydride (Fig. 5). Fortunately, all products were readily accessible with high-to-excellent conversions after 60 min reaction, which underlines the high versatility of the cascade. Very comparable trends were found in all cases with slightly lagging reaction progress when using Ac_2_O and 2-ethylhexanoic anhydride. In the case of Ac_2_O, the most hydrophilic reagent, the competition by unwanted hydrolysis with residual water in the reaction medium might presumably limit the reaction progress, compared to more productive hydrophobic acyl donors. This would also be in line with the acetylation results of 4-vinylcatechol (4VC) (lower left entry): there, the use of 2 eq. Ac_2_O (acyl donor) per phenolic-OH group (acyl acceptor) was maintained. As the substrate bears two hydroxy groups, the overall acyl donor/acceptor ratio was unchanged, while the acyl donor/water ratio was doubled. Thereby, the effect of competing water is expected to be lower, and the reaction progress of this particular acetylation was actually more comparable with the other more productive ones. In the case of 2-ethylhexanoic anhydride, its bulky nature around the electrophilic reaction center is believed to provide a kinetic barrier affecting reaction progress, but excellent conversions are obtained as well. All 15 targeted products could be straightforwardly accessed and were isolated by extractive workup yielding crude products that were characterized by HPLC, TLC, ^1^H-NMR, ^13^C-NMR, and HRMS (see Methods section and Supplementary Data). This clearly shows that the combination of highly intensified enzymatic steps with subsequent chemical reactions may become a useful strategy for future synthetic processes starting from bio-based materials.

## Conclusions

The set-up of chemoenzymatic cascades to valorize lignin-derived raw materials in an integrated manner is of utmost importance for future biorefineries. In this way, substrates can be selectively defunctionalized and upgraded to compounds of industrial interest by combining enzymes and chemocatalysts. This work constitutes a prime example, from which several findings must be emphasized as relevant for future biorefineries: (i) the combination of enzymes and base-catalysts in the same organic media simplifies the downstream processing and reduces wastes through cascade optimization and solvent recycling; (ii) the cascade is highly robust and the processes can be applied at remarkably high substrate loadings (up to 400 g·L^−1^) with full conversion and reduced reaction times (<3 h in total, covering the two steps), clearly reaching industrial requirements; (iii) the cascade shows a broad scope range of substrates, in particular for the chemical step, what enables the production of compounds with different market interests (*e.g.*, larger or shorter acyl donor chain), and creates a modular synthetic platform; and (iv) there is no need of any sophisticated process control measures such as active water control once the water needs are met and reservoirs pertinently defined.

Notably, apart from these remarkable aspects related to industrial processing, the identification and fine-tuning of critical aspects of the entire cascade – and in particular the enzymatic step – represents an important academic highlight, from which lessons can be extrapolated to many other biocatalytic reactions in non-conventional media that ought to be intensified. The present work leverages experimental assessment of enzymatic performance as a function of initial water in the system and combines it with computational means such as COSMOtherm and MD simulations to reveal the underlying competition for water in the intensified biotransformation. The relevance of keeping the water activity constant during the reaction evolution (especially in such intensified processes) has been somewhat overlooked in the past and may certainly explain many other enzymatic cases in which biocatalyst deactivations were observed, especially when enzymatic reactions are intensified. We hope that this work will serve as inspiration for other researchers developing industrial multi-step (bio)catalytic reactions in non-conventional media.

## Methods

### General information

^1^H- and ^13^C-NMR spectra were recorded on a 400 MHz instrument (*BRUKER*) at +25 °C. Chemical shifts are given in part per million (ppm) relative to solvent residual peaks of CDCl_3_ (^1^H: *δ* = 7.26 ppm, ^13^C: *δ* = 77.2 ppm) or CD_3_OD (^1^H: *δ* = 3.31 ppm, ^13^C: *δ* = 49.0 ppm) and coupling constants (*J*) are reported in Hertz (Hz). Thin layer chromatography was carried out on silica gel 60 F_254_ plates (*Merck*) and compounds were visualized by dipping into basic permanganate reagent (10 g·L^−1^ KMnO_4_, 50 g·L^−1^ Na_2_CO_3_, 0.85 g·L^−1^ NaOH, in H_2_O). High-resolution MS analytics were performed with a 6230 TOF-mass spectrometer (*Agilent Technologies*) using electrospray ionisation and time-of-flight mass selection. The phenolic acid decarboxylase used in this study (origin: *Bacillus subtilis*; PDB: 2P8G)^24^ was heterologously expressed in *E. coli*, immobilized, and assessed as described in the Supplementary Methods.

### Materials

All chemicals, materials and solvents were obtained from commercial suppliers (Acros Organics, Sigma-Aldrich, TCI Chemicals, VWR International, Merck KGaA, Thermo Fisher Scientific, ARMAR AG, J.T. Baker B. V., Biowest^®^, Scharlau Chemie S.A., Purolite Ltd.) and used as received: ferulic acid (FA, ≥99% grade), *p*-coumaric acid (*p*CA, >98.0%), caffeic acid (CA, ≥98%), NaOAc (≥99.0%), Na_2_CO_3_ (≥97%, technical), NaCl (≥99.5%), NaOH (≥98%, p.a.), Na_2_SO_4_ (≥99.0%, anhyd.), K_2_CO_3_ (≥99.0%), K_2_HPO_4_ (99%), KH_2_PO_4_ (≥ 99.5%), KMnO_4_ (≥99.0%), acetic anhydride (Ac_2_O, for synthesis), isobutyric anhydride (97%), hexanoic anhydride (98%), 2-ethylhexanoic anhydride (95%), lauric anhydride (>98%), glutaraldehyde (50 wt% in water), CPME (EMPLURA®, 100%, >10 ppm BHT), cyclohexane (≥99.5%, p.a.), ethyl acetate (EtOAc, ≥99.5%, p.a.), acetonitrile (MeCN, ≥99.8%, HPLC grade), formic acid (99-100%), CDCl_3_ (99.8 atom% D, stab. with Ag), CD_3_OD (99.8 atom% D), DMAP (≥99%), Bradford assay solution (Art. No. B5702), bovine serum albumin (BSA, lyophilized), CalB immo Plus^TM^ (on ECR1030M, Purolite Ltd.), and amino-functionalized Lifetech^TM^ ECR8415F (Purolite Ltd.) for enzyme immobilization.

### Thermodynamic equilibrium calculations

The activity coefficients and the solubility of water in mixtures with ferulic acid (FA), 4-vinylguaiacol (4VG) and cyclopentyl methyl ether (CPME) were calculated using BIOVIA COSMOtherm 2020^20–23^. The conformer sets for all molecules were generated with COSMOconf v3.0 and the COSMO calculations were performed with Turbomole v6.6^25^ using the TZVPD-FINE parametrization. The solubility of water in the respective mixtures was calculated using the equation for a liquid-liquid-equilibrium:

$${x}_{{{{{{\rm{i}}}}}}}^{{{{{{\rm{I}}}}}}}\cdot {\gamma }_{{{{{{\rm{i}}}}}}}^{{{{{{\rm{I}}}}}}}={x}_{{{{{{\rm{i}}}}}}}^{{{{{{\rm{II}}}}}}}\cdot {\gamma }_{{{{{{\rm{i}}}}}}}^{{{{{{\rm{II}}}}}}}$$

with ${x}_{{{{{{\rm{i}}}}}}}^{{{{{{\rm{I}}}}}}}$ and ${x}_{{{{{{\rm{i}}}}}}}^{{{{{{\rm{II}}}}}}}$ as composition of the two phases ${{{{{\rm{I}}}}}}$ and ${{{{{\rm{II}}}}}}$ and ${\gamma }_{{{{{{\rm{i}}}}}}}^{{{{{{\rm{I}}}}}}}$ and ${\gamma }_{{{{{{\rm{i}}}}}}}^{{{{{{\rm{II}}}}}}}$ as the activity coefficients for component ${{{{{\rm{i}}}}}}$ in the respective phase.

### MD simulations

The enzyme *Bs*PAD (PDB entry: 2P8G)^24^ was modelled with the CHARMM36m^26^ force field in the MD simulations. The solvent molecules and reactants (CPME, FA, 4VG) were modelled using the CHARMM General Force Field (CGenFF) version 4.X^27,28^. Initial topologies for these molecules and the protonation state of the enzyme at neutral pH were generated for GROMACS by the CHARMM-GUI^29–31^. The CHARMM-TIP3P variant^32,33^ was used to represent water in the MD simulations. A cut-off radius *r*_cut_ = 1.2 nm was applied for the electrostatic and Lennard-Jones interactions, whereby the force of the Lennard-Jones interactions was smoothly switched between 1.0 and 1.2 nm. Long-range electrostatic interactions were modelled using the smooth Particle-Mesh Ewald (PME) method^34^ with a PME order of 4 and a spacing of 0.18 nm. All bonds with hydrogen atoms have been fixed with LINCS^35^ for *Bs*PAD and the solvent/reactant molecules and with the SETTLE algorithm^36^ for water.

**Fig. 6:**
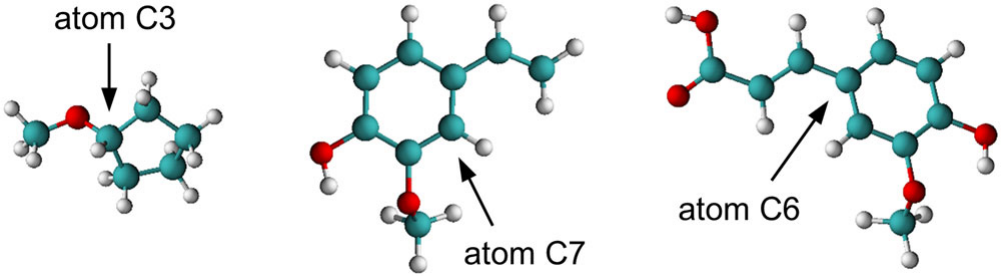
Molecular structures of CPME, 4VG and FA as used for MD simulations. The central atoms used for the determination of their position with respect to the enzyme surface are highlighted.

All MD simulations in this work were carried out using GROMACS 2019.4^37–39^. Starting with one dimeric *Bs*PAD structure, (PDB entry: 2P8G)^24^ cubic boxes with periodic boundary conditions including the respective number of solvent molecules were built using PACKMOL^40^. The positions of 14 buried water molecules in the dimeric *Bs*PAD structure were preserved and 24 sodium ions were added to the simulations box for charge neutrality as this is necessary for the PME calculations. The simulations did not include any further buffer salts, neither did the laboratory experiments. Depending on the simulated concentrations of reagents, the final equilibrated cubic box dimensions were between 9.61-9.75 nm. An energy minimization using the steepest decent algorithm was performed for 5000 steps. Afterwards, three consecutive NVT simulations for 0.5 ns each were performed, whereby all non-hydrogen protein atoms, the backbone and the C*α*–atoms of *Bs*PAD were constraint with a harmonic potential (1,000 kJ·mol^−1^·nm^−2^), respectively. This stepwise release of the enzyme structure allowed for a smooth equilibration of the solvent molecules around the *Bs*PAD without artificially distorting the enzyme in the MD simulations and has already been used elsewhere^41^. Newton’s equations of motion were numerically solved using the leapfrog algorithm^42^ with a time step of d*t* = 1 fs. The temperature of the system was controlled to 303.15 K (30 °C) using the velocity rescale thermostat^43^ with a time constant of ${\tau }_{T}$ = 1 ps. The three NVT simulations were followed by an NPT simulation for 2 ns to equilibrate the pressure of the system to 1 bar using the Berendsen barostat^44^ with a time constant of ${\tau }_{{{{{{\rm{p}}}}}}}$ = 5 ps and an isothermal compressibility of ${\kappa }_{{{{{{\rm{T}}}}}}}$ = 5 bar^−1^. The time step for the numerical integration of Newton’s equation of motion was thereby increased to 2 fs. The equilibration was followed by a sampling simulation in the NPT ensemble for 100 ns by switching the pressure control to Parrinello-Rahman^45^. To ensure proper convergence of the enzyme structure and the hydration shell composition, the last 40 ns of sampling simulation were used to analyse the distribution of solvent molecules around the enzyme structure. Figure 6 shows representations of the molecular structures of CPME, FA, and 4VG as used in the MD simulations. The central atoms used for the distribution analysis and the calculation of bulk phase concentrations are highlighted for each molecule.

The determination of the water concentration in the bulk phase of the MD simulations containing one *Bs*PAD enzyme structure is based on the method from Wedberg et al.^46^. In order to identify the distance from the protein surface to the bulk phase r_bulk_, radial concentration profiles for water ${x}_{{{{{{\rm{H}}}}}}2{{{{{\rm{O}}}}}}}(r)$ were calculated from statistical averages of the amount of water, CPME, FA, 4VG, and sodium ions in dependency of the distance r from the enzyme with a shell size of 0.05 nm.

$${x}_{{{{{{\rm{H}}}}}}2{{{{{\rm{O}}}}}}}\left(r\right)=\frac{\left\langle {N}_{{{{{{\rm{H}}}}}}2{{{{{\rm{O}}}}}}}(r)\right\rangle }{\left\langle {N}_{{{{{{\rm{H}}}}}}2{{{{{\rm{O}}}}}}}(r)\right\rangle +\left\langle {N}_{{{{{{\rm{CPME}}}}}}}(r)\right\rangle +\left\langle {N}_{{{{{{\rm{Na}}}}}}}(r)\right\rangle +\left\langle {N}_{{{{{{\rm{FA}}}}}}}(r)\right\rangle +\left\langle {N}_{4{{{{{\rm{VG}}}}}}}(r)\right\rangle }$$

Based on the convergence of ${x}_{{{{{{\rm{H}}}}}}2{{{{{\rm{O}}}}}}}(r)$ towards a constant value, ${r}_{{{{{{\rm{bulk}}}}}}}$ was selected using a threshold of change of <0.1% in ${x}_{{{{{{\rm{H}}}}}}2{{{{{\rm{O}}}}}}}(r)$. This was done for all simulations in which the water concentration in the bulk phase ${x}_{{{{{{\rm{H}}}}}}2{{{{{\rm{O}}}}}}}({{{{{\rm{bulk}}}}}})$ was calculated. An example of a radial concentration profile is shown in Supplementary Fig. S2 for an MD simulation with 4000 CPME molecules, 1400 water molecules and 24 sodium ions surrounding a single dimeric *Bs*PAD enzyme structure. The same methodology was employed to find the initial equilibrium state (no FA/4VG) in which both the CPME solvent phase and the enzyme surface (pseudo-phase) are fully water saturated. Here, the total amount of water molecules was gradually increased, the system equilibrated and the respective increase of water in the bulk phase evaluated. The results are illustrated in Supplementary Fig. S3 and show an initially hesitant increase of bulk phase water (first two points), followed by a roughly linear dependency (up to ${N}_{H2O}$ = 1000), a levelling off and subsequent fluctuations. This trend is explained by the initial predominant coordination of water to the most polar amino acids on the enzyme surface, followed by a more balanced competition between enzyme and CPME and an eventual oversaturation in which excess water forms droplets, causing the observed fluctuations. Based on this, the MD system showed water saturation at ${N}_{H2O}$ = 1400.

### Intensified biocatalytic decarboxylation

The phenolic acid substrate (FA, *p*CA, CA) was suspended in the respective amount of wet CPME (equilibrated over water at 25 °C, final dissolved water content ~0.7 wt%) to give a nominal substrate loading of 0.1–2.0 M. Next, up to 14 mL additional H_2_O as well as up to 30 g moist ECR8415F beads were added per litre of the reaction mixture. Both these additions sustain water activity under intensified reaction conditions (>0.1 M) and were accordingly increased with substrate loading. To start the reaction, immobilized *Bs*PAD-8415F preparation (5–40 g·L^−1^) was added and the reaction suspension subjected to 30 °C and 1000 rpm on a benchtop Thermoshaker. Samples were taken periodically, quenched, and diluted in water/acetonitrile (1/1), vortexed, centrifuged (13 200 rpm, 2 min), and subjected to HPLC analysis. Herein, the immobilized enzyme concentration (g L^−1^) refers to the grams of the beads in litre of total reaction volume. 8415F carrier material showed 95% immobilization yield for *Bs*PAD using the target protein loading of 50 mg protein per g wet carrier and the crude cell-free extract applied (protein content of 40 wt%).

### Intensified chemocatalytic acylation

Following complete decarboxylation of phenolic acids (FA, *p*CA, CA) in wet CPME by heterogeneous *Bs*PAD-8415F catalyst, the reaction mixture consists of a homogeneous solution of the respective hydroxystyrenes in moist CPME and the solid biocatalyst beads. From this, the beads were removed, 2 eq. of carboxylic anhydride as well as 0.05 eq. of sodium carbonate were added, and the reaction mixture was subjected to 90 °C and 1000 rpm on a benchtop Thermoshaker. Samples were taken periodically, quenched, and diluted in water/acetonitrile (1/1), vortexed, centrifuged (13,200 rpm, 2 min), and subjected to HPLC analysis.

### High-performance liquid chromatography

Quantitative analyses were performed with a *Shimadzu* HPLC system (dwell volume determined to 0.33 mL) consisting of a SCL-10Avp system controller, a DGU-14A degasser, two LC-10ADvp pumps, a SIL-10ADvp autoinjector, a CTO-10ASvp column oven, and an SPD-M10Avp detector, all controlled by LabSolutions software (*Shimadzu*). The sample injection volume was 10 μL, the column oven temperature 35 ± 0.5 °C, the total flow rate 0.7 mL·min^−1^. Water and acetonitrile (MeCN) mixed with 0.1 vol% formic acid were used as eluents in two different methods for the model cascade FA–4VG–AMS (method A) and product screening (method B). Method A (isocratic): 0–10 min (55% MeCN), on a Kinetex® 5 μm C18 100 Å, 250 × 4.6 mm column (*Phenomenex*, 00G-4601-E0) protected by a SecurityGuard ULTRA C18 guard column. Method B (gradient): 0–10 min (13% to 98% MeCN), 10–18 min (98% MeCN), 18 min (step to 13% MeCN), 18–22 min (13% MeCN), on a Kinetex® 5 μm C8 100 Å, 150 × 4.6 mm column (*Phenomenex*, 00F-4608-E0) protected by a SecurityGuard ULTRA C8 guard column. The 300 nm channel of the diode array detector was used for calibration and quantification of ferulic acid, *p*-coumaric acid and caffeic acid. For conversion control using substrate-to-product ratios, the 254 nm channel was used. Calibrations and example chromatograms showing the separation of substrates from all their showcased products are provided in the Supplementary Note in Figures S4–S7.

### Characterization of hydroxystyrenes

As full conversion of the biocatalytic decarboxylation was confirmed by HPLC, the reaction solution was transferred to a centrifuge tube, dried over Na_2_SO_4_, and centrifuged (2 min, 13 200 rpm). The supernatant was transferred to a round-bottom flask and removal of the solvent under reduced pressure afforded hydroxystyrenes of high quality:

4-vinylphenol [4VP]: colourless crystals. purity: >99% (^1^H-NMR). TLC (silica gel 60, cyclohexane/EtOAc = 9:1): *R*_f_ = 0.18. ^1^H-NMR (400 MHz, CD_3_OD): δ [ppm] = 5.02 (1H, dd, *J*_1_ = 10.9 Hz, *J*_2_ = 1.1 Hz, *cis*-CH = CH_2_), 5.55 (1H, dd, *J*_1_ = 17.6 Hz, *J*_2_ = 1.1 Hz, *trans*-CH = CH_2_), 6.62 (1H, dd, *J*_1_ = 17.6 Hz, *J*_2_ = 10.9 Hz, –CH = CH_2_), 6.70–6.75 (2H, m, ar-H), 7.22–7.27 (2H, m, ar-H). ^13^C-NMR (400 MHz, CD_3_OD): δ [ppm] = 110.7, 116.2 (2 C), 128.4 (2 C), 130.7, 137.8, 158.4.

4-vinylguaiacol [4VG]: clear oil. purity: 99% (^1^H-NMR, impurity: grease). TLC (silica gel 60, cyclohexane/EtOAc = 4:1): *R*_f_ = 0.38. ^1^H-NMR (400 MHz, CDCl_3_): δ [ppm] = 3.92 (3H, s, –OCH_3_), 5.13 (1H, dd, *J*_1_ = 10.8 Hz, *J*_2_ = 0.8 Hz, *cis*-CH = CH_2_), 5.59 (1H, dd, *J*_1_ = 17.6 Hz, *J*_2_ = 0.8 Hz, *trans*-CH = CH_2_), 5.64 (1H, s, –OH), 6.64 (1H, dd, *J*_1_ = 17.6 Hz, *J*_2_ = 10.9 Hz, –CH = CH_2_), 6.87 (1H, d, *J* = 8.0 Hz, ar-H), 6.92 (1H, dd, *J*_1_ = 8.1 Hz, *J*_2_ = 1.8 Hz, ar-H), 6.94 (1H, d, *J* = 1.8 Hz, ar-H). ^13^C-NMR (400 MHz, CDCl_3_): δ [ppm] = 56.1, 108.2, 111.6, 114.5, 120.2, 130.4, 136.8, 145.8, 146.7.

4-vinylcatechol [4VC]: clear oil. purity: 95% (^1^H-NMR, impurity: residual CPME). TLC (silica gel 60, cyclohexane/EtOAc = 4:1): *R*_f_ = 0.16. ^1^H-NMR (400 MHz, CD_3_OD): δ [ppm] = 5.00 (1H, dd, *J*_1_ = 10.9 Hz, *J*_2_ = 1.1 Hz, *cis*-CH = CH_2_), 5.51 (1H, dd, *J*_1_ = 17.6 Hz, *J*_2_ = 1.2 Hz, *trans*-CH = CH_2_), 6.55 (1H, dd, *J*_1_ = 17.6 Hz, *J*_2_ = 10.9 Hz, –CH = CH_2_), 6.70 (1H, d, *J* = 8.1 Hz, ar-H), 6.73 (1H, dd, *J*_1_ = 8.2 Hz, *J*_2_ = 1.9 Hz, ar-H), 6.88 (1H, d, *J* = 1.9 Hz, ar-H). ^13^C-NMR (400 MHz, CD_3_OD): δ [ppm] = 110.7, 113.6, 116.2, 119.7, 131.4, 138.1, 146.3, 146.5.

### Characterization of acylated hydroxystyrenes

As reaction control *via* HPLC confirmed near-complete conversion by chemocatalytic acylation, reactions were quenched by addition of water and stoichiometric amounts of Na_2_CO_3_. The resulting phases were separated. For lauroyloxy-decorated styrenes, a clean phase separation was facilitated by dispersing the obtained emulsion (1.9 mL) in 100 mL H_2_O/CPME (1/1). The organic phases were washed with sat. aq. NaCl solution, the aqueous phases removed, the organic phases dried over Na_2_SO_4_, the supernatants transferred to round-bottom flasks, and the solvent removed on a rotary evaporator. This general procedure afforded crude products of varying quality as reported below.

4-acetoxystyrene [AS]: 14.5 mg (89%) clear oil. purity: >99% (HPLC, 254 nm), 99% (^1^H-NMR, impurity: CPME). TLC (silica gel 60, cyclohexane/EtOAc = 9:1): *R*_f_ = 0.41. ^1^H-NMR (400 MHz, CDCl_3_): δ [ppm] = 2.30 (3H, s, –CO–CH_3_), 5.24 (1H, dd, *J*_1_ = 10.9 Hz, *J*_2_ = 0.7 Hz, *cis*-CH = CH_2_), 5.70 (1H, dd, *J*_1_ = 17.6 Hz, *J*_2_ = 0.7 Hz, *trans*-CH = CH_2_), 6.70 (1H, dd, *J*_1_ = 17.6 Hz, *J*_2_ = 10.9 Hz, –CH = CH_2_), 7.03–7.07 (2H, m, ar-H), 7.40–7.43 (2H, m, ar-H). ^13^C-NMR (400 MHz, CDCl_3_): δ [ppm] = 21.3, 114.2, 121.8 (2 C), 127.4 (2 C), 135.6, 136.1, 150.4, 169.7. HRMS calcd for C_10_H_11_O_2_^+^ [M + H^+^]: 163.0759, found: 163.0758.

4-isobutanoyloxystyrene [IBS]: 16.5 mg (87%) clear, yellowish oil. purity: 97.0% (HPLC, 254 nm), 85% (^1^H-NMR, impurities: grease, CPME). TLC (silica gel 60, cyclohexane/EtOAc = 9:1): *R*_f_ = 0.69. ^1^H-NMR (400 MHz, CDCl_3_): δ [ppm] = 1.32 (6H, d, *J* = 7.0 Hz, –CH_3_), 2.80 (1H, sept, *J* = 7.0 Hz, –CO–CH(–CH_3_)_2_), 5.24 (1H, dd, *J*_1_ = 10.9 Hz, *J*_2_ = 0.7 Hz, *cis*-CH = CH_2_), 5.70 (1H, dd, *J*_1_ = 17.6 Hz, *J*_2_ = 0.7 Hz, *trans*-CH = CH_2_), 6.70 (1H, dd, *J*_1_ = 17.6 Hz, *J*_2_ = 10.9 Hz, –CH = CH_2_), 7.01–7.06 (2H, m, ar-H), 7.39–7.43 (2H, m, ar-H). ^13^C-NMR (400 MHz, CDCl_3_): δ [ppm] = 19.1 (2 C), 34.4, 114.1, 121.7 (2 C), 127.3 (2 C), 135.4, 136.1, 150.6, 175.8. HRMS calcd for C_12_H_15_O_2_^+^ [M + H^+^]: 191.1072, found: 191.1072.

4-hexanoyloxystyrene [HS]: 20.5 mg (94%) clear, yellow oil. purity: 96.9% (HPLC, 254 nm), 82% (^1^H-NMR, impurities: grease, hexanoic acid, CPME). TLC (silica gel 60, cyclohexane/EtOAc = 9:1): *R*_f_ = 0.73. ^1^H-NMR (400 MHz, CDCl_3_): δ [ppm] = 0.93 (3H, t, *J* = 7.1 Hz, –CH_2_–CH_3_), 1.39 (4H, m, –CH_2_–), 1.76 (2H, qu, *J* = 7.4 Hz, –CO–CH_2_–CH_2_–), 2.55 (2H, t, *J* = 7.5 Hz, –CO–CH_2_–), 5.24 (1H, d, *J* = 10.8 Hz, *cis*-CH = CH_2_), 5.70 (1H, d, *J* = 17.6 Hz, *trans*-CH = CH_2_), 6.70 (1H, dd, *J*_1_ = 17.6 Hz, *J*_2_ = 10.9 Hz, –CH = CH_2_), 7.01–7.07 (2H, m, ar-H), 7.38–7.44 (2H, m, ar-H). ^13^C-NMR (400 MHz, CDCl_3_): δ [ppm] = 14.1, 22.5, 24.8, 31.4, 34.5, 114.1, 121.8 (2 C), 127.3 (2 C), 135.4, 136.1, 150.5, 172.5. HRMS calcd for C_14_H_19_O_2_^+^ [M + H^+^]: 219.1385, found: 219.1385.

4-(2-ethyl-hexanoyloxy)styrene [EHS]: 51.6 mg (150%) clear, yellowish oil. purity: 92.8% (HPLC, 254 nm), 49% (^1^H-NMR, impurities: 2-ethylhexanoic acid, grease). TLC (silica gel 60, cyclohexane/EtOAc = 9:1): *R*_f_ = 0.80. ^1^H-NMR (400 MHz, CDCl_3_): δ [ppm] = 0.93 (3H, t, *J* = 7.2 Hz, –CH_3_), 1.02 (3H, t, *J* = 7.4 Hz, –CH_3_), 1.31–1.40 (4H, m, –CH_2_–CH_2_–CH_3_), 1.58–1.63 (2H, m, –CH_2_–), 1.73–1.81 (2H, m, –CH_2_–), 2.47–2.54 (1H, m, –CH–), 5.23 (1H, dd, *J*_1_ = 10.9 Hz, *J*_2_ = 0.7 Hz, *cis*-CH = CH_2_), 5.70 (1H, dd, *J*_1_ = 17.6 Hz, *J*_2_ = 0.8 Hz, *trans*-CH = CH_2_), 6.70 (1H, dd, *J*_1_ = 17.6 Hz, *J*_2_ = 10.9 Hz, –CH = CH_2_), 7.01–7.04 (2H, m, ar-H), 7.39–7.43 (2H, m, ar-H). ^13^C-NMR (400 MHz, CDCl_3_): δ [ppm] = 12.1, 14.2, 22.8, 25.7, 29.8, 32.0, 47.6, 114.1, 121.9 (2 C), 127.3 (2 C), 135.4, 136.1, 150.6, 175.0. HRMS calcd for C_16_H_23_O_2_^+^ [M + H^+^]: 247.1698, found: 247.1704.

4-lauroyloxystyrene [LS]: 61.1 mg (144%) clear, yellowish oil. purity: 94.8% (HPLC, 254 nm), 65% (^1^H-NMR, impurities: lauric acid, grease). TLC (silica gel 60, cyclohexane/EtOAc = 9:1): *R*_f_ = 0.83. ^1^H-NMR (400 MHz, CDCl_3_): δ [ppm] = 0.88 (3H, t, *J* = 6.7 Hz, –CH_3_), 1.27 (16H, m, –CH_2_–), 1.75 (2H, p, *J* = 7.5 Hz, –CH_2_–CH_2_–CO–), 2.55 (2H, t, *J* = 7.5 Hz, –CH_2_–CO–), 5.24 (1H, dd, *J*_1_ = 10.9 Hz, *J*_2_ = 0.7 Hz, *cis*-CH = CH_2_), 5.70 (1H, dd, *J*_1_ = 17.6 Hz, *J*_2_ = 0.8 Hz, *trans*-CH = CH_2_), 6.70 (1H, dd, *J*_1_ = 17.6 Hz, *J*_2_ = 10.9 Hz, –CH = CH_2_), 7.02–7.05 (2H, m, ar-H), 7.39–7.42 (2H, m, ar-H). ^13^C-NMR (400 MHz, CDCl_3_): δ [ppm] = 14.1, 22.9, 25.1, 29.3, 29.4, 29.5, 29.6, 29.8 (2 C), 32.1, 34.6, 114.1, 121.8 (2 C), 127.3 (2 C), 135.4, 136.1, 150.5, 172.5. HRMS calcd for C_20_H_31_O_2_^+^ [M + H^+^]: 303.2324, found: 303.2340.

4-acetoxy-3-methoxystyrene [AMS]: 17.8 mg (93%) clear, yellowish oil. purity: 99.4% (HPLC, 254 nm), 98% (^1^H-NMR, impurity: CPME). TLC (silica gel 60, cyclohexane/EtOAc = 4:1): *R*_f_ = 0.44. ^1^H-NMR (400 MHz, CDCl_3_): δ [ppm] = 2.31 (3H, s, –CO–CH_3_), 3.85 (3H, s, –OCH_3_), 5.25 (1H, dd, *J*_1_ = 10.9 Hz, *J*_2_ = 0.6 Hz, *cis*-CH = CH_2_), 5.70 (1H, dd, *J*_1_ = 17.5 Hz, *J*_2_ = 0.7 Hz, *trans*-CH = CH_2_), 6.68 (1H, dd, *J*_1_ = 17.5 Hz, *J*_2_ = 10.9 Hz, –CH = CH_2_), 6.97–7.02 (3H, m, ar-H). ^13^C-NMR (400 MHz, CDCl_3_): δ [ppm] = 20.8, 56.0, 110.0, 114.3, 119.1, 122.9, 136.4, 136.8, 139.6, 151.2, 169.3. HRMS calcd for C_11_H_13_O_3_^+^ [M + H^+^]: 193.0865, found: 193.0866.

4-isobutanoyloxy-3-methoxystyrene [IBMS]: 18.6 mg (84%) yellow oil. purity: 98.3% (HPLC, 254 nm), 85% (^1^H-NMR, impurities: grease, CPME). TLC (silica gel 60, cyclohexane/EtOAc = 4:1): *R*_f_ = 0.61. ^1^H-NMR (400 MHz, CDCl_3_): δ [ppm] = 1.33 (6H, d, *J* = 7.0 Hz, –CH_3_), 2.81 (1H, sept, *J* = 7.0 Hz, –CO–CH(–CH_3_)_2_), 3.84 (3H, s, –OCH_3_), 5.24 (1H, dd, *J*_1_ = 10.9 Hz, *J*_2_ = 0.7 Hz, *cis*-CH = CH_2_), 5.69 (1H, dd, *J*_1_ = 17.6 Hz, *J*_2_ = 0.7 Hz, *trans*-CH = CH_2_), 6.68 (1H, dd, *J*_1_ = 17.6 Hz, *J*_2_ = 10.9 Hz, –CH = CH_2_), 6.97–7.01 (3H, m, ar-H). ^13^C-NMR (400 MHz, CDCl_3_): δ [ppm] = 19.2, 34.2, 56.0, 110.1, 114.1, 119.1, 122.9, 136.5, 136.6, 139.9, 151.3, 175.4. HRMS calcd for C_13_H_16_O_3_Na^+^ [M+Na^+^]: 243.0997, found: 243.1003.

4-hexanoyloxy-3-methoxystyrene [HMS]: 24.2 mg (97%) yellowish oil. purity: 99.7% (HPLC, 254 nm), 72% (^1^H-NMR, impurities: CPME, grease, hexanoic acid). TLC (silica gel 60, cyclohexane/EtOAc = 4:1): *R*_f_ = 0.67. ^1^H-NMR (400 MHz, CDCl_3_): δ [ppm] = 0.93 (3H, t, *J* = 7.1 Hz, –CH_3_), 1.40 (4H, m, –CH_2_–), 1.77 (2H, qu, *J* = 7.5 Hz, –CO–CH_2_–CH_2_–), 2.57 (2H, t, *J* = 7.5 Hz, –CO–CH_2_–), 3.84 (3H, s, –OCH_3_), 5.24 (1H, dd, *J*_1_ = 10.9 Hz, *J*_2_ = 0.6 Hz, *cis*-CH = CH_2_), 5.69 (1H, dd, *J*_1_ = 17.6 Hz, *J*_2_ = 0.7 Hz, *trans*-CH = CH_2_), 6.68 (1H, dd, *J*_1_ = 17.6 Hz, *J*_2_ = 10.9 Hz, –CH = CH_2_), 6.97–7.01 (3H, m, ar-H). ^13^C-NMR (400 MHz, CDCl_3_): δ [ppm] = 14.1, 22.5, 24.9, 31.4, 34.2, 56.0, 110.0, 114.2, 119.1, 123.0, 136.5, 136.7, 139.7, 151.3, 172.1. HRMS calcd for C_15_H_21_O_3_^+^ [M + H^+^]: 249.1491, found: 249.1495.

4-(2-ethyl-hexanoyloxy)−3-methoxystyrene [EHMS]: 56.6 mg (146%) yellowish oil. purity: 95.8% (HPLC, 254 nm), 64% (^1^H-NMR, impurities: 2-ethylhexanoic acid, grease). TLC (silica gel 60, cyclohexane/EtOAc = 4:1): *R*_f_ = 0.79. ^1^H-NMR (400 MHz, CDCl_3_): δ [ppm] = 0.93 (3H, t, *J* = 7.1 Hz, –CH_3_), 1.04 (3H, t, *J* = 7.4 Hz, –CH_3_), 1.37–1.43 (4H, m, –CH_2_–CH_2_–CH_3_), 1.61–1.68 (2H, m, –CH_2_–) 1.73–1.83 (2H, m, –CH_2_–), 2.50–2.57 (1H, m, –CH–), 3.83 (3H, s, –OCH_3_), 5.24 (1H, dd, *J*_1_ = 10.9 Hz, *J*_2_ = 0.6 Hz, *cis*-CH = CH_2_), 5.69 (1H, dd, *J*_1_ = 17.6 Hz, *J*_2_ = 0.7 Hz, *trans*-CH = CH_2_), 6.68 (1H, dd, *J*_1_ = 17.6 Hz, *J*_2_ = 10.9 Hz, –CH = CH_2_), 6.93–7.00 (3H, m, ar-H). ^13^C-NMR (400 MHz, CDCl_3_): δ [ppm] = 11.9, 14.2, 22.9, 25.8, 29.7, 32.1, 47.5, 55.8, 110.0, 114.1, 119.1, 123.0, 136.5, 136.6, 139.8, 151.4, 174.5. HRMS calcd for C_17_H_24_O_3_Na^+^ [M+Na^+^]: 299.1623, found: 299.1622.

4-lauroyloxy-3-methoxystyrene [LMS]: 66.8 mg (144%) yellowish oil. purity: 97.0% (HPLC, 254 nm), 17% (^1^H-NMR, impurities: mainly lauric acid, grease). TLC (silica gel 60, cyclohexane/EtOAc = 4:1): *R*_f_ = 0.87. ^1^H-NMR (400 MHz, CDCl_3_): δ [ppm] = 0.88 (3H, t, *J* = 6.9 Hz, –CH_3_), 1.26 (16H, m, –CH_2_–), 1.76 (2H, p, *J* = 7.5 Hz, –CH_2_–CH_2_–CO–), 2.57 (2H, t, *J* = 7.5 Hz, –CH_2_–CO–), 3.84 (3H, s, –OCH_3_), 5.24 (1H, dd, *J*_1_ = 10.9 Hz, *J*_2_ = 0.5 Hz, *cis*-CH = CH_2_), 5.69 (1H, dd, *J*_1_ = 17.6 Hz, *J*_2_ = 0.6 Hz, *trans*-CH = CH_2_), 6.68 (1H, dd, *J*_1_ = 17.6 Hz, *J*_2_ = 10.9 Hz, –CH = CH_2_), 6.97–7.00 (3H, m, ar-H). ^13^C-NMR (400 MHz, CDCl_3_): δ [ppm] = 14.3, 22.9, 25.2, 29.2, 29.5 (2 C), 29.7, 29.8 (2 C), 32.1, 34.3, 56.0, 110.0, 114.2, 119.1, 123.0, 136.5, 136.7, 139.7, 151.3, 172.1. HRMS calcd for C_21_H_32_O_3_Na^+^ [M+Na^+^]: 355.2249, found: 355.2248.

3,4-diacetoxystyrene [DAS]: 28.1 mg (75%) yellowish oil. purity: 99.5% (HPLC, 254 nm), 85% (^1^H-NMR, impurities: grease, CPME). TLC (silica gel 60, cyclohexane/EtOAc = 9:1): *R*_f_ = 0.15. ^1^H-NMR (400 MHz, CDCl_3_): δ [ppm] = 2.28 (3H, s, –CO–CH_3_), 2.29 (3H, s, –CO–CH_3_), 5.28 (1H, d, *J* = 11.1 Hz, *cis*-CH = CH_2_), 5.70 (1H, d, *J* = 17.5 Hz, *trans*-CH = CH_2_), 6.66 (1H, dd, *J*_1_ = 17.6 Hz, *J*_2_ = 10.9 Hz, –CH = CH_2_), 7.14 (1H, d, *J* = 8.4 Hz, ar-H), 7.22 (1H, d, *J* = 2.0 Hz, ar-H), 7.27 (1H, dd, *J*_1_ = 8.4 Hz, *J*_2_ = 2.0 Hz, ar-H). ^13^C-NMR (400 MHz, CDCl_3_): δ [ppm] = 20.8 (2 C), 115.2, 121.0, 123.5, 124.6, 135.4, 136.8, 141.7, 142.3, 168.4 (2 C). HRMS calcd for C_12_H_12_O_4_Na^+^ [M+Na^+^]: 243.0633, found: 243.0635.

3,4-diisobutanoyloxystyrene [DIBS]: 28.0 mg (59%) yellowish oil. purity: 96.9% (HPLC, 254 nm), 89% (^1^H-NMR, impurities: grease, CPME). TLC (silica gel 60, cyclohexane/EtOAc = 9:1): *R*_f_ = 0.44. ^1^H-NMR (400 MHz, CDCl_3_): δ [ppm] = 1.30 (6H, d, *J* = 7.0 Hz, –CH_3_), 1.31 (6H, d, *J* = 7.0 Hz, –CH_3_), 2.78 (1H, sept, *J* = 7.0 Hz, –CO–CH(–CH_3_)_2_), 2.79 (1H, sept, *J* = 7.0 Hz, –CO–CH(–CH_3_)_2_), 5.27 (1H, dd, *J*_1_ = 10.9 Hz, *J*_2_ = 0.5 Hz, *cis*-CH = CH_2_), 5.70 (1H, dd, *J*_1_ = 17.6 Hz, *J*_2_ = 0.5 Hz, *trans*-CH = CH_2_), 6.66 (1H, dd, *J*_1_ = 17.6 Hz, *J*_2_ = 10.9 Hz, –CH = CH_2_), 7.12 (1H, d, *J* = 8.4 Hz, ar-H), 7.19 (1H, d, *J* = 2.0 Hz, ar-H), 7.26 (1H, dd, *J*_1_ = 8.4 Hz, *J*_2_ = 2.0 Hz, ar-H). ^13^C-NMR (400 MHz, CDCl_3_): δ [ppm] = 19.1 (4 C), 34.2 (2 C), 115.0, 121.0, 123.5, 124.4, 135.5, 136.6, 141.9, 142.5, 174.5 (2 C). HRMS calcd for C_16_H_20_O_4_Na^+^ [M+Na^+^]: 299.1259, found: 299.1253.

3,4-dihexanoyloxystyrene [DHS]: 39.5 mg (70%) yellowish oil. purity: 98.0% (HPLC, 254 nm), 89% (^1^H-NMR, impurities: grease, hexanoic acid, CPME). TLC (silica gel 60, cyclohexane/EtOAc = 9:1): *R*_f_ = 0.60. ^1^H-NMR (400 MHz, CDCl_3_): δ [ppm] = 0.93 (6H, m, –CH_2_–CH_3_), 1.38 (8H, m, –CH_2_–), 1.74 (4H, m, –CO–CH_2_–CH_2_–), 2.53 (4H, m, –CO–CH_2_–), 5.27 (1H, dd, *J*_1_ = 10.8 Hz, *J*_2_ = 0.4 Hz, *cis*-CH = CH_2_), 5.69 (1H, dd, *J*_1_ = 17.5 Hz, *J*_2_ = 0.4 Hz, *trans*-CH = CH_2_), 6.66 (1H, dd, *J*_1_ = 17.6 Hz, *J*_2_ = 10.9 Hz, –CH = CH_2_), 7.13 (1H, d, *J* = 8.4 Hz, ar-H), 7.21 (1H, d, *J* = 2.0 Hz, ar-H), 7.26 (1H, dd, *J*_1_ = 8.4 Hz, *J*_2_ = 2.0 Hz, ar-H). ^13^C-NMR (400 MHz, CDCl_3_): δ [ppm] = 14.1 (2 C), 22.5 (2 C), 24.8 (2 C), 31.4 (2 C), 34.2 (2 C), 115.1, 121.1, 123.6, 124.5, 135.5, 136.6, 141.8, 142.4, 171.3 (2 C). HRMS calcd for C_20_H_28_O_4_Na^+^ [M+Na^+^]: 355.1885, found: 355.1881.

3,4-bis(2-ethyl-hexanoyloxy)styrene [DEHS]: 79.9 mg (149%) yellowish oil. purity: 88.0% (HPLC, 254 nm), 39% (^1^H-NMR, impurities: 2-ethylhexanoic acid, grease). TLC (silica gel 60, cyclohexane/EtOAc = 9:1): *R*_f_ = 0.76. ^1^H-NMR (400 MHz, CDCl_3_): δ [ppm] = 0.96 (6H, t, *J* = 7.5 Hz, –CH_3_), 1.01 (3H, t, *J* = 7.4 Hz, –CH_3_), 1.02 (3H, t, *J* = 7.5 Hz, –CH_3_), 1.30–1.41 (8H, m, –CH_2_–CH_2_–CH_3_), 1.49–1.82 (8H, m, –CH_2_–), 2.45–2.53 (2H, m, –CH–), 5.26 (1H, dd, *J*_1_ = 10.8 Hz, *J*_2_ = 0.5 Hz, *cis*-CH = CH_2_), 5.69 (1H, dd, *J*_1_ = 17.5 Hz, *J*_2_ = 0.5 Hz, *trans*-CH = CH_2_), 6.66 (1H, dd, *J*_1_ = 17.6 Hz, *J*_2_ = 10.9 Hz, –CH = CH_2_), 7.12 (1H, d, *J* = 8.4 Hz, ar-H), 7.17 (1H, d, *J* = 2.0 Hz, ar-H), 7.26 (1H, dd, *J*_1_ = 8.4 Hz, *J*_2_ = 2.0 Hz, ar-H). ^13^C-NMR (400 MHz, CDCl_3_): δ [ppm] = 12.0 (2 C), 14.1 (2 C), 22.9 (2 C), 25.3 (2 C), 29.7, 29.8, 31.5 (2 C), 47.0, 47.1, 115.0, 121.3, 123.7, 124.2, 135.6, 136.4, 141.9, 142.5, 174.0, 174.1. HRMS calcd for C_24_H_36_O_4_Na^+^ [M+Na^+^]: 411.2511, found: 411.2508.

3,4-dilauroyloxystyrene [DLS]: 9.6 mg (14%) oily residue. purity: 77.0% (HPLC, 254 nm), 63% (^1^H-NMR, impurities: lauric acid, acetone). TLC (silica gel 60, cyclohexane/EtOAc = 9:1): *R*_f_ = 0.74. ^1^H-NMR (400 MHz, CDCl_3_): δ [ppm] = 0.88 (3H, t, *J* = 6.8 Hz, –CH_3_), 1.27 (32H, m, –CH_2_–), 1.73 (2H, p, *J* = 7.5 Hz, –CH_2_–CH_2_–CO–), 1.73 (2H, p, *J* = 7.5 Hz, –CH_2_–CH_2_–CO–), 2.52 (2H, t, *J* = 7.6 Hz, –CH_2_–CO–), 2.53 (2H, t, *J* = 7.5 Hz, –CH_2_–CO–), 5.28 (1H, d, *J* = 11.0 Hz, *cis*-CH = CH_2_), 5.69 (1H, d, *J* = 17.6 Hz, *trans*-CH = CH_2_), 6.65 (1H, d, *J*_1_ = 17.6 Hz, *J*_2_ = 10.9 Hz, –CH = CH_2_), 7.13 (1H, d, *J* = 8.4 Hz, ar-H), 7.20 (1H, d, *J* = 2.0 Hz, ar-H), 7.26 (1H, dd, *J*_1_ = 8.3 Hz, *J*_2_ = 2.0 Hz, ar-H). ^13^C-NMR (400 MHz, CDCl_3_): δ [ppm] = 14.3 (2 C), 22.9 (2 C), 25.2 (2 C), 29.4 (2 C), 29.5 (2 C), 29.5 (2 C), 29.7 (2 C), 29.8 (4 C), 32.1 (2 C), 34.3 (2 C), 115.1, 121.1, 123.6, 124.5, 135.5, 136.6, 141.8, 142.4, 171.3 (2 C). HRMS calcd for C_32_H_52_O_4_Na^+^ [M+Na^+^]: 523.3763, found: 523.3778.

### Reporting summary

Further information on research design is available in the Nature Portfolio Reporting Summary linked to this article.

### Supplementary information

Supplementary Information

Description of Additional Supplementary Files

Supplementary Data 1

Reporting Summary

## Data Availability

Supplementary methods, discussions, and notes are provided in the Supplementary Information, whereas all NMRs are reported as Supplementary Data. Additionally, the detailed datasets generated during the current study, including both experimental and computational data, are publicly available in the Zenodo open data repository, https://zenodo.org/records/10545172.
