# Supplementary material for: Integrated preservation of water activity as key to intensified chemoenzymatic synthesis of bio-based styrene derivatives
Source: Commun Chem. 2024 Mar 14;7:57. doi: 10.1038/s42004-024-01138-x (PMC10940287; doi:10.1038/s42004-024-01138-x)
Supplement: Supplementary file 1 — Supplementary Information [file 42004_2024_1138_MOESM1_ESM.pdf]

Communications Chemistry

Supplementary Information (SI)

Integrated Preservation of Water Activity as Key to Intensified Chemoenzymatic Synthesis of Bio-Based Styrene Derivatives

Philipp Petermeier[1], Jan Philipp Bittner[2], Tobias Jonsson[3], Pablo Domínguez de María[4], Emil Byström[5], and Selin Kara[1,6*]

[1] Department of Biological and Chemical Engineering, Aarhus University, 8000 Aarhus C, Denmark.

[2] Institute of Thermal Separation Processes, Hamburg University of Technology, 21073 Hamburg, Germany.

[3] Diduco AB, Tvistevägen 48C, 90736 Umeå, Sweden.

[4] Sustainable Momentum SL, Av. Ansite 3, 4-6, 35011 Las Palmas de Gran Canaria, Canary Islands, Spain.

[5] SpinChem AB, Tvistevägen 48C, 90736 Umeå, Sweden.

[6] Institute of Technical Chemistry, Leibniz University Hannover, 30167 Hannover, Germany.

email: selin.kara@bce.au.dk

Contents:

*Bs*PAD: expression in *E. coli* BL21-Gold(DE3) 2
*Bs*PAD: protein assay 2
*Bs*PAD: covalent immobilization on amino-functionalized carriers 2
*Bs*PAD: activity assay in aqueous media 3
*Bs*PAD: activity assay in CPME 3
Process modularity: screening of acylation conditions 4
MD simulations: illustrations for methodology 6
High-performance liquid chromatography 7

Supplementary Methods

*Bs*PAD: expression in *E. coli* BL21-Gold(DE3)

The plasmid encoding for the phenolic acid decarboxylase was provided by Prof. Robert Kourist from TU Graz. Competent cells of *E. coli* BL21-Gold(DE3) (Art. No. 230132, *Agilent Technologies*) were transformed with the plasmid according to the cell supplier's instructions. A single colony from an agar plate was used to inoculate LB medium (20 mL, 50 mg·L^{-1} Kanamycin) and incubated at 37 °C and 150 rpm overnight. Glycerol stocks were prepared by diluting 700 µL of this overnight culture (ONC) with 300 µL of 60% (m/m) aqueous glycerol. The stocks were kept at room temperature for 15 min, then flash-frozen in liquid nitrogen and stored at -20 °C (work stock) and -80 °C (backup stock). For enzyme expression, LB medium (20 mL, 50 mg·L^{-1} Kanamycin) was inoculated with 50 µL of cell suspension from a thawed glycerol stock and incubated at 37 °C and 150 rpm for 16-18 h in a 100 mL baffled Erlenmeyer flask. 10 mL of ONC were used to inoculate TB medium (1 L, 50 mg·L^{-1} Kanamycin) and the resulting culture was incubated at 37 °C and 80 rpm in a 5 L baffled Erlenmeyer flask. Samples (1 mL) were taken at regular intervals and analysed for their optical density at 600 nm (OD_{600}). When the OD_{600} reached a value of about 1.5 (approx. after 3 h), protein expression was induced by addition of IPTG (1 mL, 1 mol·L^{-1} in H_2O) and incubation was continued at 20 °C and 80 rpm for about 21 h. For harvesting, cultures were centrifuged (8,000 rpm, 4 °C, 20 min) to yield wet cell pellets. The supernatant was treated with Virkon® and discarded. The pelleted cells were resuspended in KPi buffer (50 mM, pH 6.0, 6 mL per gram wet cell pellet), cell suspensions (in fractions of 50 mL) transferred to round-bottom flask (250 mL), flash-frozen in liquid nitrogen and lyophilized overnight to obtained lyophilized whole-cell preparations (typically 2.3-3.2 g·L^{-1}), which were stored at -20 °C until use. For the preparation of cell-free extracts (CFE), lyophilized whole-cells (1 g) were transferred to 50 mL plastic tubes and resuspended in Milli-Q water (25 mL). The cell suspensions were cooled on wet ice for 1 h before ultrasonic cell disruption (MS73 probe, 20%, 2 s ON, 4 s OFF, 2 min per cycle, 4 cycles). Next, the suspensions were transferred to 50 mL centrifuge tubes and centrifuged (14,000 rpm, 4 °C, 20 min) to remove cell debris. The supernatants were transferred to a round-bottom flask (250 mL), flash-frozen in liquid nitrogen and lyophilized overnight to obtain lyophilized cell-free extract preparations, which were stored at -20 °C until use.

*Bs*PAD: protein assay

Protein concentrations of crude *Bs*PAD were determined with the Bradford protein assay. Calibrations were based on BSA standards in the range of 100−500 mg·L^{-1} in aq. 0.15 M NaCl solution. The same NaCl solution was used as blank (0 mg·L^{-1} protein) and to dilute samples when necessary. Samples and standards were measured analogously as follows: 20 µL of sample volume were transferred to a PS cuvette and mixed with 980 µL of ready-to-use Bradford assay solution. The mixture was incubated at room temperature (5 min) and absorbance measured at 595 nm using a U-1900 HITACHI spectrophotometer (*Hitachi*, Japan). The calibration was done new for every measurement series with an R^2 > 0.995.

*Bs*PAD: covalent immobilization on amino-functionalized carriers

Prior to enzyme immobilization, Lifetech™ ECR 8415F beads were washed three times with KPi buffer (50 mM, pH 6.0) using a resin/buffer ratio of 1/1 (w/v). Next, the beads were incubated with glutaraldehyde buffer (2 vol% in KPi buffer, 50 mM, pH 6.0) in a resin/buffer ratio of 1/4 (w/v). The slurry was mixed for 1 h at room temperature on a self-made end-over-end mixer (60 rpm) before the supernatant was removed and

the beads washed 4 times with KPi buffer (50 mM, pH 6.0) at a resin/buffer ratio of 1/1 (w/v). A solution of 25 mg·L^{-1} crude CFE (protein content approx. 40%) was prepared in immobilization buffer (KPi, 50 mM, pH 6.0). Washed carrier beads were mixed with the crude enzyme solution in a resin/buffer ratio of 1/4 (w/v). For 250 mg beads this step was conducted in 2 mL microcentrifuge tubes, whereas for 10 g beads 50 mL Falcon tubes were used. The slurries were gently mixed for 18 h at room temperature on the end-over-end mixer (60 rpm). Lastly, the mixtures were spun down (2 mL tubes: 13 200 rpm, 1 min; 50 mL tubes: 4 000 rpm, 1 min), the supernatant removed, the beads washed twice with KPi buffer (50 mM, pH 6.0) at a resin/buffer ratio of 1/2 (w/v) each, and all supernatants (original and both washings) combined. The protein content in the collected liquid phase was determined by Bradford protein assay and used to calculate immobilization yield. Wet immobilized enzyme preparations were stored in closed vessels at +4 °C.

***Bs*PAD: activity assay in aqueous media**

This standard assay was performed in KPi buffer (50 mM, pH 6.0) using the model substrate ferulic acid at an initial concentration of 10 mM ensuring zero-order kinetics. The assay was used for both free and immobilized enzyme. For the former, 900 µL KPi buffer were mixed with 50 µL FA stock (200 mM in DMSO) and tempered to 30 °C on a Thermoshaker. To start the reaction, 50 µL resuspended enzyme preparation (1 mg·mL^{-1} whole-cells or CFE in KPi buffer) were added and the mixture subjected to 30 °C and 1000 rpm. For immobilized enzyme, roughly 5 mg enzyme preparation were resuspended in 1425 µL KPi buffer and tempered to 30 °C. To start the reaction, 75 µL FA stock (200 mM in DMSO) were added and the mixture subjected to 30 °C and 1000 rpm. Samples of 100 µL were quenched and diluted by addition to 900 µL water/acetonitrile (1/1), vortexed, centrifuged (13 200 rpm, 2 min), and subjected to HPLC analysis. Standard sampling times for whole-cell and CFE preparations were 120, 160, 200, 240, and 285 s, whereas for immobilized enzyme preparations they were 2, 5, 10, 15, 20, 30, 45, 60, and 90 min. The derived kinetics data was based on a progressive curve analysis of substrate depletion and used to control the enzymatic activity employed in or recovered from experiments.

***Bs*PAD: activity assay in CPME**

This standard assay was performed in wet CPME (equilibrated over water at room temperature) using the model substrate ferulic acid at an initial concentration of 100 mM. The assay was used for both free and immobilized enzyme. For the former, 19 mg FA were dissolved in 900 µL of wet CPME and tempered to 30 °C on a Thermoshaker. To start the reaction, 100 µL of resuspended enzyme preparation (1 mg·mL^{-1} in wet CPME) were added and the mixture subjected to 30 °C and 1000 rpm. For immobilized enzyme, roughly 5 mg enzyme preparation were charged with 1.5 mL of FA solution (100 mM in wet CPME, tempered to 30 °C) and the mixture subjected to 30 °C and 1000 rpm. Samples of 10 µL were quenched and diluted by addition to 990 µL water/acetonitrile (1/1), vortexed, centrifuged (13 200 rpm, 2 min), and subjected to HPLC analysis. Standard sampling times for whole-cell and CFE preparations were within 2–80 min, whereas for immobilized enzyme preparations they were 2, 5, 10, 15, 20, 30, 45, 60, and 90 min. The derived kinetics data was based on a progressive curve analysis of substrate depletion and used to control the enzymatic activity employed in or recovered from experiments.

Supplementary Discussion

Process modularity: screening of acylation conditions

In order to find promising alternative acylation conditions, *i.e.*, different acyl donors, catalysts, and pairings thereof, a range of options were tested in the acylation of 4-vinylguaiacol (4VG). The results are compiled below.

Table S 1. Screening of acylation conditions for 4VG in CPME. All results are based on experimental duplicates and reaction progress was judged by product peak area share in the combined substrate and product peak area total (HPLC, 254 nm). Reaction conditions: 100 mM 4VG in wet CPME, 0.9 mL total volume, 1000 rpm, 90 min, 90 °C (for CalB entries: 60 °C). Abbreviations: DMAP, 4-dimethylaminopyridine; AMS, 4-acetoxy-3-methoxystyrene; HMS, 4-hexanoyloxy-3-methoxystyrene; BMS, 4-benzoyloxy-3-methoxystyrene.

acyl donor	catalyst	product	reaction progress
2 eq. Ac_2O	0.05 eq. NaOAc	AMS	95%
2 eq. Ac_2O	0.05 eq. K_2CO_3	AMS	78%
2 eq. Ac_2O	0.05 eq. Na_2CO_3	AMS	95%
2 eq. Ac_2O	10 wt% CalB immo Plus^{TM}	AMS	4%
2 eq. acetyl chloride	0.05 eq. Na_2CO_3	AMS	1%
2 eq. acetyl chloride	2 eq. Na_2CO_3	AMS	2%
2 eq. acetyl chloride	0.05 eq. DMAP, 1 eq. Na_2CO_3	AMS	2%
2 eq. isopropenyl acetate	0.05 eq. Na_2CO_3	AMS	<1%
2 eq. isopropenyl acetate	10 wt% CalB immo Plus^{TM}	AMS	<1%
2 eq. vinyl acetate	0.05 eq. Na_2CO_3	AMS	<1%
2 eq. vinyl acetate	10 wt% CalB immo Plus^{TM}	AMS	<1%
2 eq. hexanoyl chloride	0.05 eq. Na_2CO_3	HMS	6%
2 eq. hexanoyl chloride	2 eq. Na_2CO_3	HMS	9%
2 eq. hexanoyl chloride	0.05 eq. DMAP	HMS	7%
2 eq. hexanoyl chloride	0.05 eq. DMAP, 1 eq. Na_2CO_3	HMS	14%
2 eq. benzoyl chloride	0.05 eq. Na_2CO_3	BMS	8%
2 eq. benzoyl chloride	2 eq. Na_2CO_3	BMS	23%
2 eq. benzoyl chloride	0.05 eq. DMAP	BMS	7%
2 eq. benzoyl chloride	0.05 eq. DMAP, 1 eq. Na_2CO_3	BMS	18%

To assess whether these findings can be translated to related hydroxystyrene substrates, a comparative assay was performed with 4-vinylphenol (4VP). This substrate lacks the methoxy residue in *ortho*-position to the hydroxy group and thus does not form an intramolecular hydrogen bond, which might influence its nucleophilic reactivity. The experiments were conducted analogously to 4VG, and the results are depicted in Figure S 1. No significant differences between the two substrates were found. Not the substrates but the reaction conditions control conversion and give comparable results. This substantiates the notion that earlier findings (Table S 1) are applicable to a broader range of substrates. Therefore, the high modularity of the chemocatalytic step in terms of carboxylic anhydride choice $(RCO)_2O$ can be combined with the range of substrates accepted by the biocatalytic step (FA, *p*CA, CA), rendering the overall cascade a modular platform with a broad product scope, depending on industrial needs.

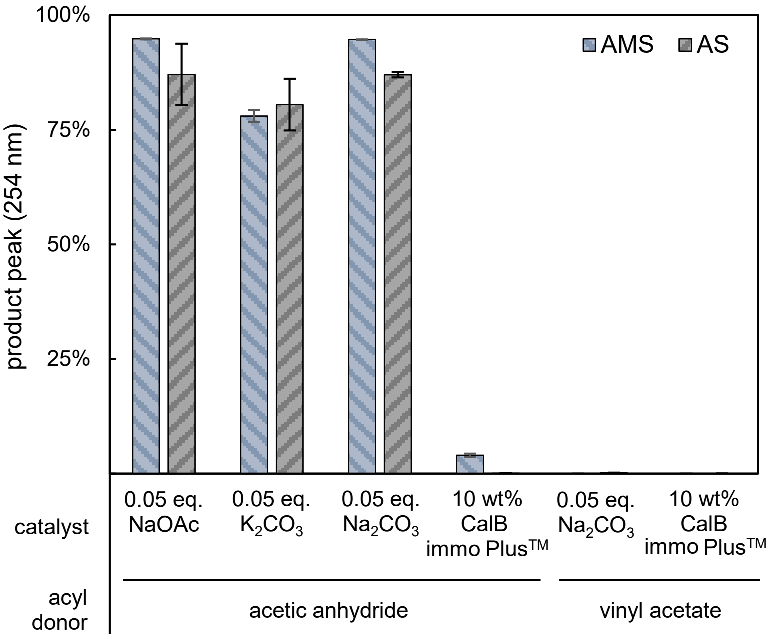

Figure S 1. Comparison of the two hydroxystyrenes 4-vinylguaiacol (4VG) and 4-vinylphenol (4VP) in their acetylation to 4-acetoxy-3-methoxystyrene (AMS) and 4-acetoxystyrene (AS), respectively. Results are based on experimental duplicates and relative product peak areas (HPLC, 254 nm) with error bars representing standard deviations. Reaction conditions: 100 mM hydroxystyrene in wet CPME, 2 eq. acyl donor, 0.9 mL total volume, 1000 rpm, 90 °C (for CalB entries: 60 °C).

Supplementary Note

MD simulations: illustrations for methodology

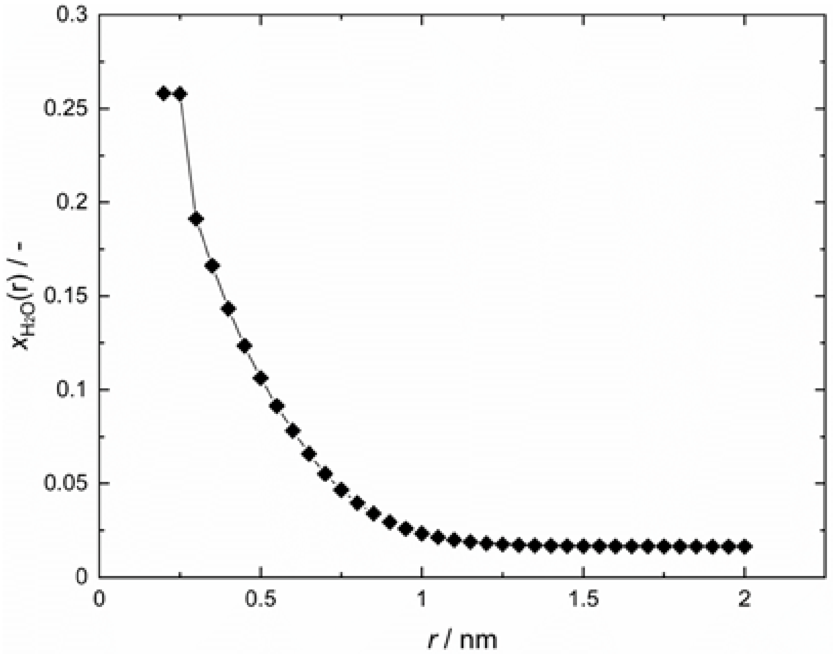

Figure S 2. Radial concentration profile of water from the enzyme surface in the surrounding bulk phase, for a system of 1 dimeric *Bs*PAD enzyme, 4000 CPME and 1400 water molecules, and 24 sodium ions.

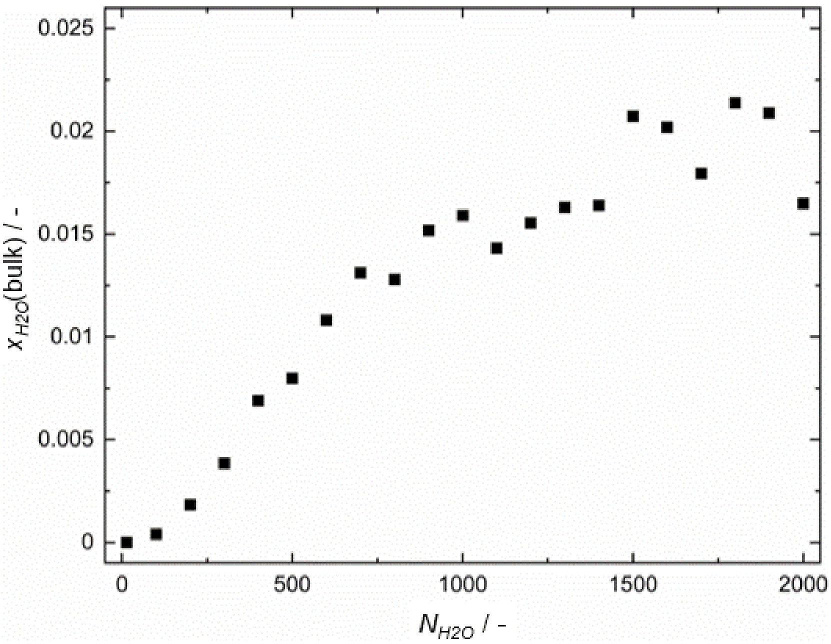

Figure S 3. Dependency of bulk phase water concentration x_{H2O}(bulk) on total available water N_{H2O} in our CPME/*Bs*PAD system.

High-performance liquid chromatography

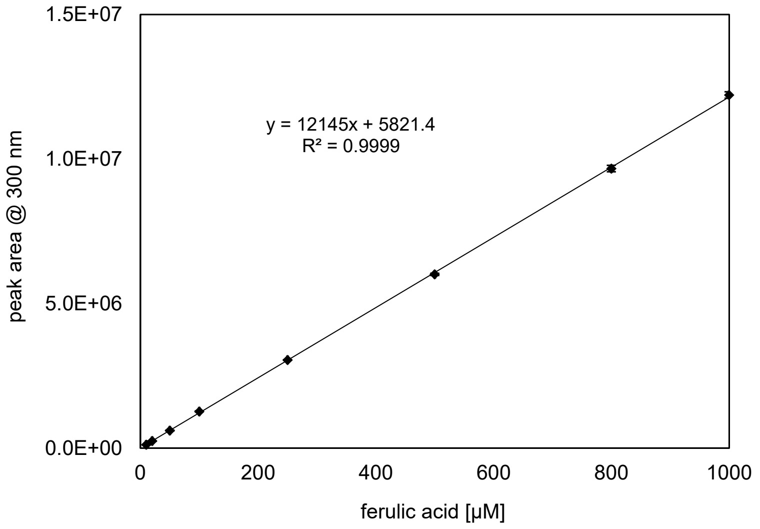

Figure S 4. Calibration for ferulic acid. (triplicates in water/MeCN 1/1; 10 µL injection volume; λ = 300 nm)

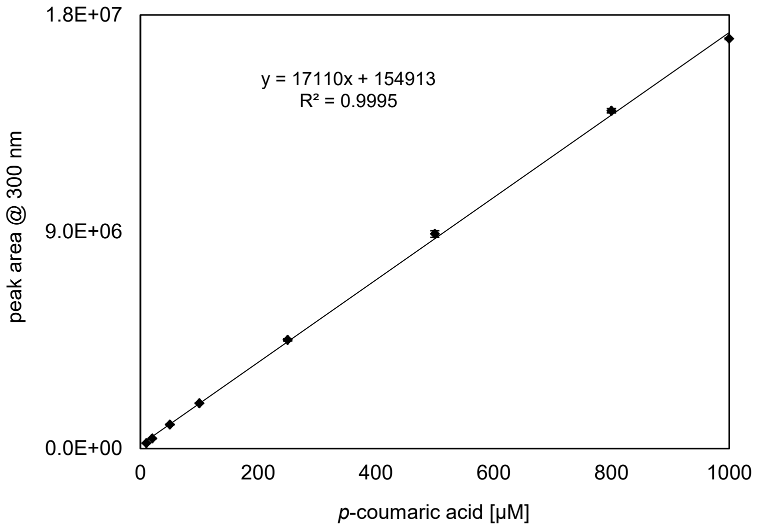

Figure S 5. Calibration for *p*-coumaric acid. (triplicates in H_2O/MeCN 1/1; 10 µL injection volume; λ = 300 nm)

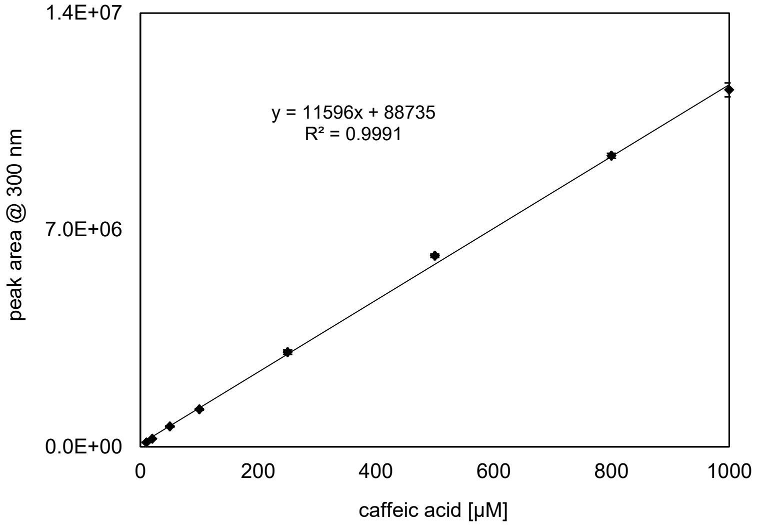

Figure S 6. Calibration for caffeic acid. (triplicates in water/MeCN 1/1; 10 µL injection volume; λ = 300 nm)

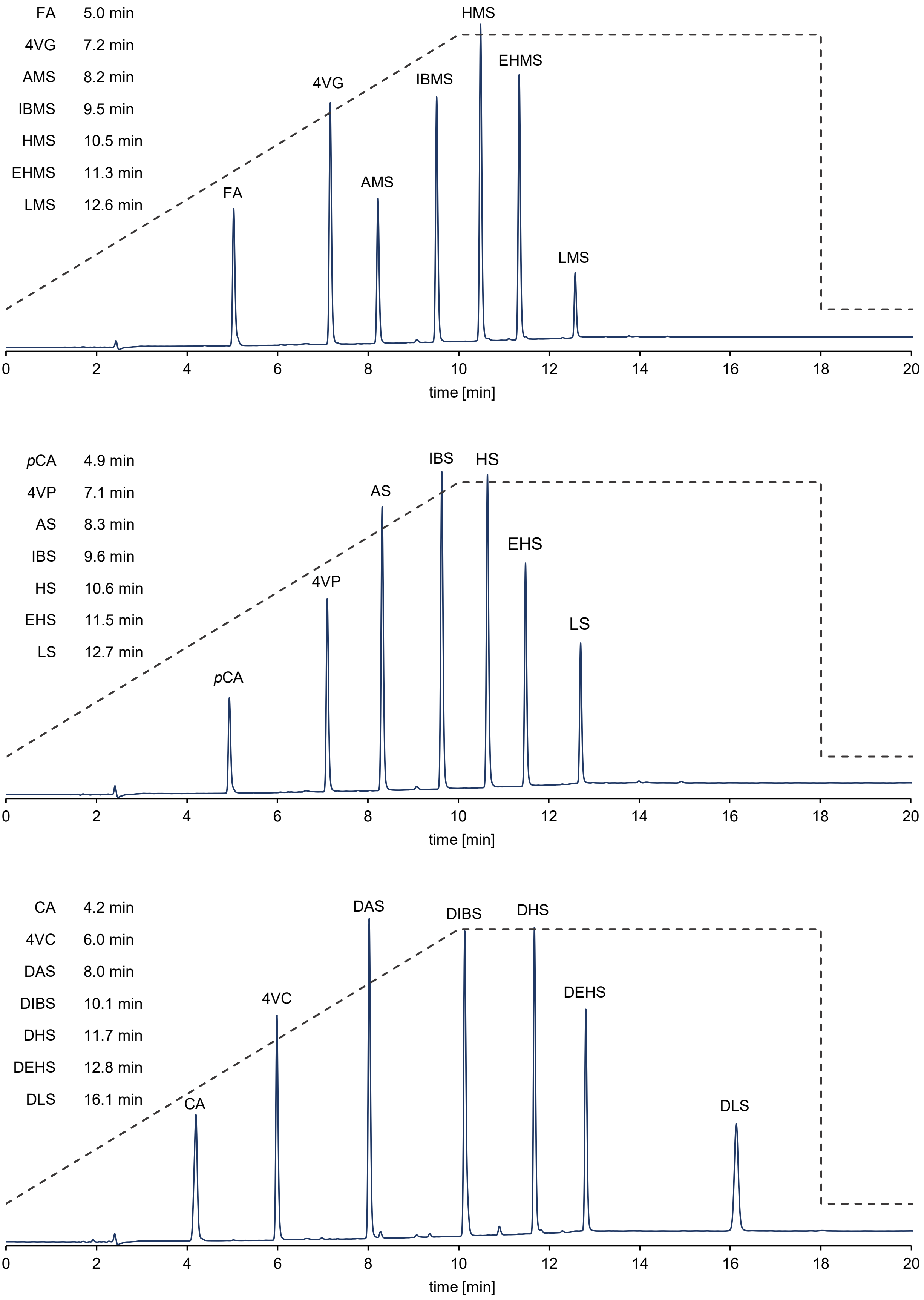

Figure S 7. Chromatographic separation of substrates (FA / *p*CA / CA), decarboxylated derivatives (4VG / 4VP / 4VC), and exemplary acylated hydroxystyrenes using HPLC gradient method B (13–98% MeCN as signified by grey dashed line) on a C8 column.
