# Supplementary material for: Integrated preservation of water activity as key to intensified chemoenzymatic synthesis of bio-based styrene derivatives
Source: Commun Chem. 2024 Mar 14;7:57. doi: 10.1038/s42004-024-01138-x (PMC10940287; doi:10.1038/s42004-024-01138-x)
Supplement: Supplementary file 2 — Description of Additional Supplementary Files [file 42004_2024_1138_MOESM2_ESM.pdf]

Description of Additional Supplementary Files

File name: Supplementary Data

Description: NMR Spectra
