# Supplementary material for: Integrated preservation of water activity as key to intensified chemoenzymatic synthesis of bio-based styrene derivatives
Source: Commun Chem. 2024 Mar 14;7:57. doi: 10.1038/s42004-024-01138-x (PMC10940287; doi:10.1038/s42004-024-01138-x)
Supplement: Supplementary file 3 — Supplementary Data 1 [file 42004_2024_1138_MOESM3_ESM.pdf]

Communications Chemistry

Supplementary Data: NMR

Integrated Preservation of Water Activity as Key to Intensified Chemoenzymatic Synthesis of Bio-Based Styrene Derivatives

Philipp Petermeier[1], Jan Philipp Bittner[2], Tobias Jonsson[3], Pablo Domínguez de María[4], Emil Byström[5], and Selin Kara[1,6]*

[1] Department of Biological and Chemical Engineering, Aarhus University, 8000 Aarhus C, Denmark.

[2] Institute of Thermal Separation Processes, Hamburg University of Technology, 21073 Hamburg, Germany.

[3] Diduco AB, Tvistevägen 48C, 90736 Umeå, Sweden.

[4] Sustainable Momentum SL, Av. Ansite 3, 4-6, 35011 Las Palmas de Gran Canaria, Canary Islands, Spain.

[5] SpinChem AB, Tvistevägen 48C, 90736 Umeå, Sweden.

[6] Institute of Technical Chemistry, Leibniz University Hannover, 30167 Hannover, Germany.

email: selin.kara@bce.au.dk

Contents:

NMR Spectra.....2

Supplementary References..... 38

NMR Spectra

All NMR spectra of synthesized hydroxystyrenes and crude acylated hydroxystyrenes are provided herein. Contaminations were identified and reported based on coupling pattern and chemical shifts from literature.[1, 2]

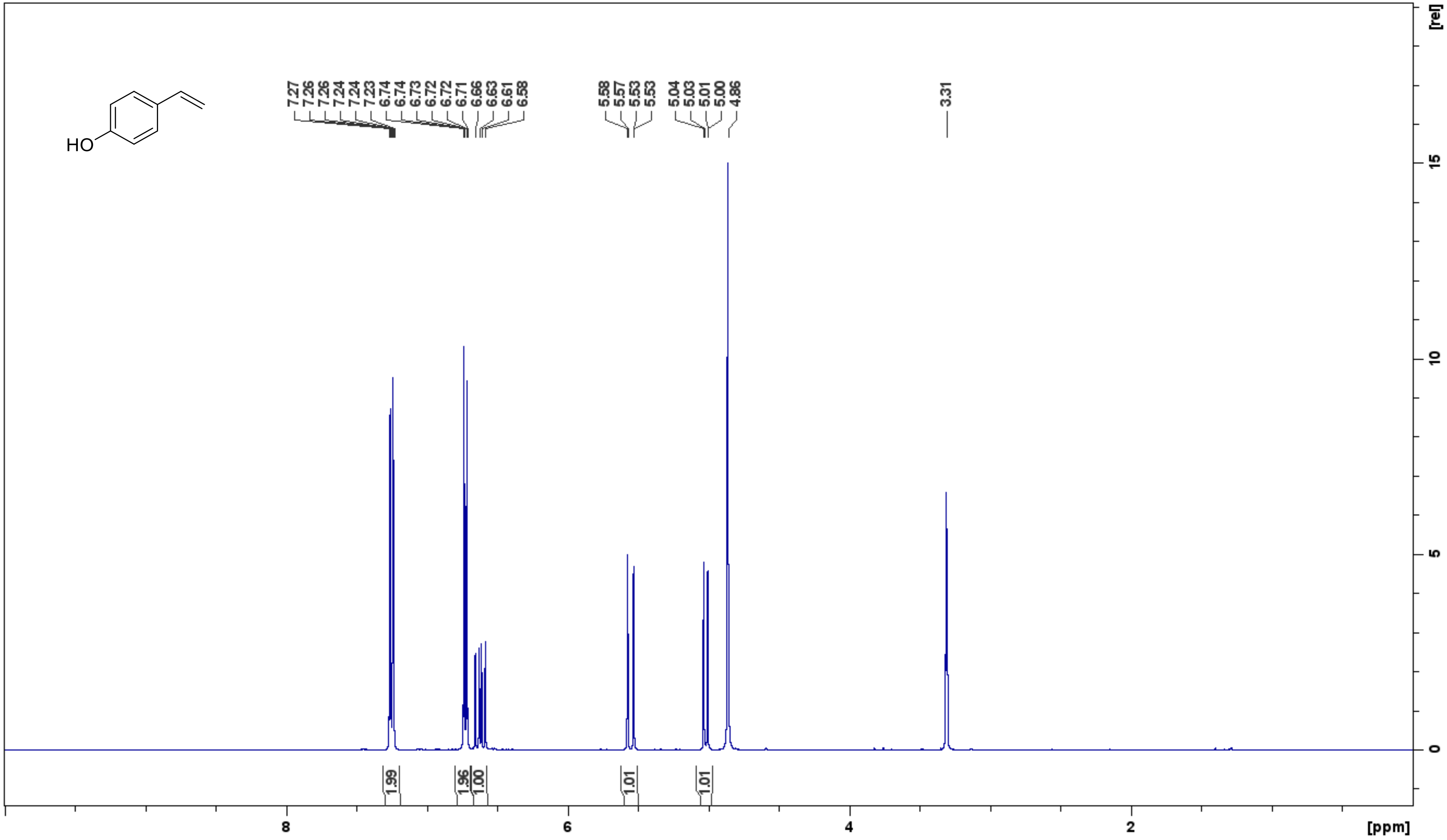

Figure SD 1. ^{1}H-NMR spectrum of 4-vinylphenol (4VP) in CD_3OD at 25 °C. Non-integrated peaks from residual NMR solvent (3.31 ppm) and moisture (4.86 ppm).

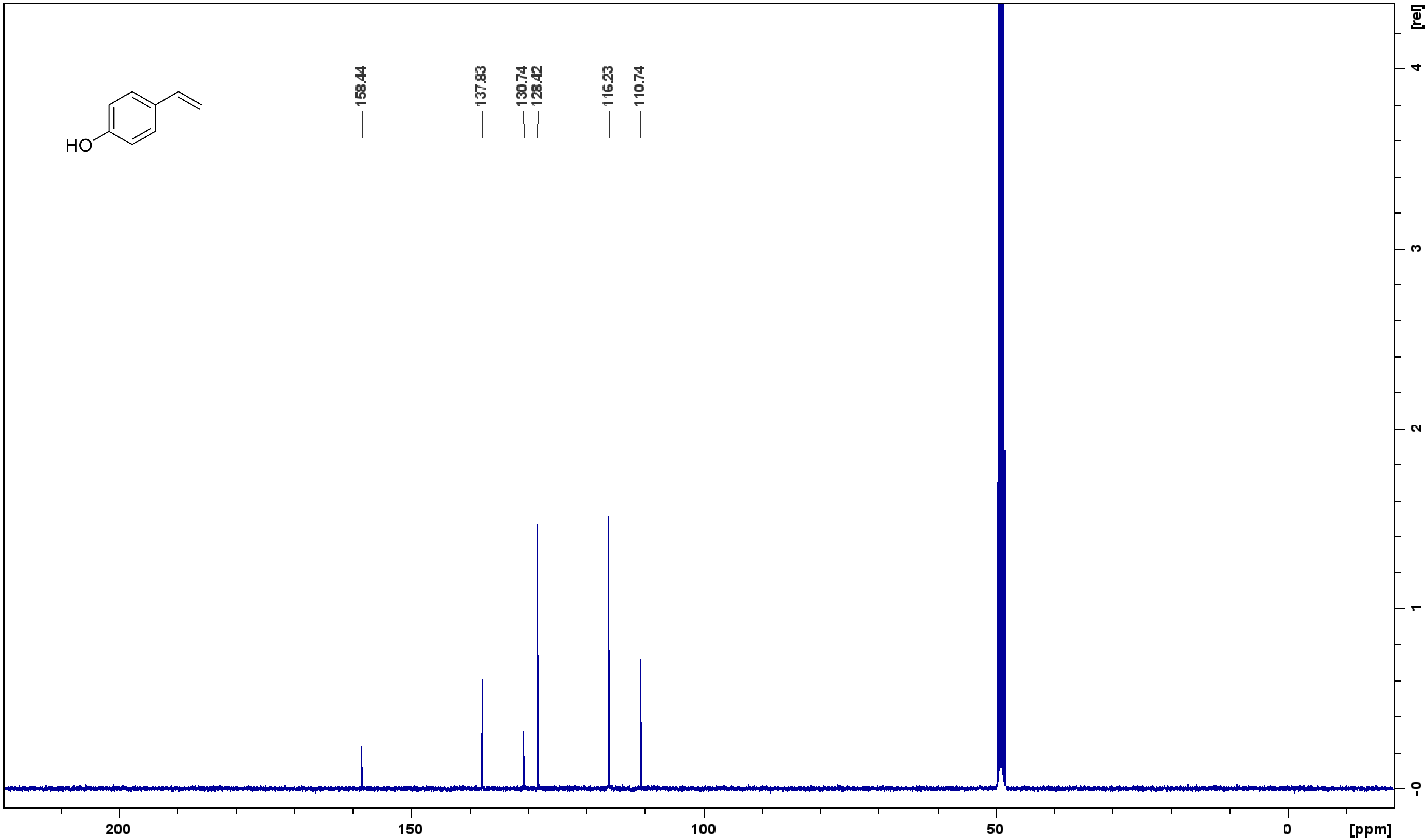

Figure SD 2. ^{13}C-NMR spectrum of 4-vinylphenol (4VP) in CD_3OD at 25 °C.

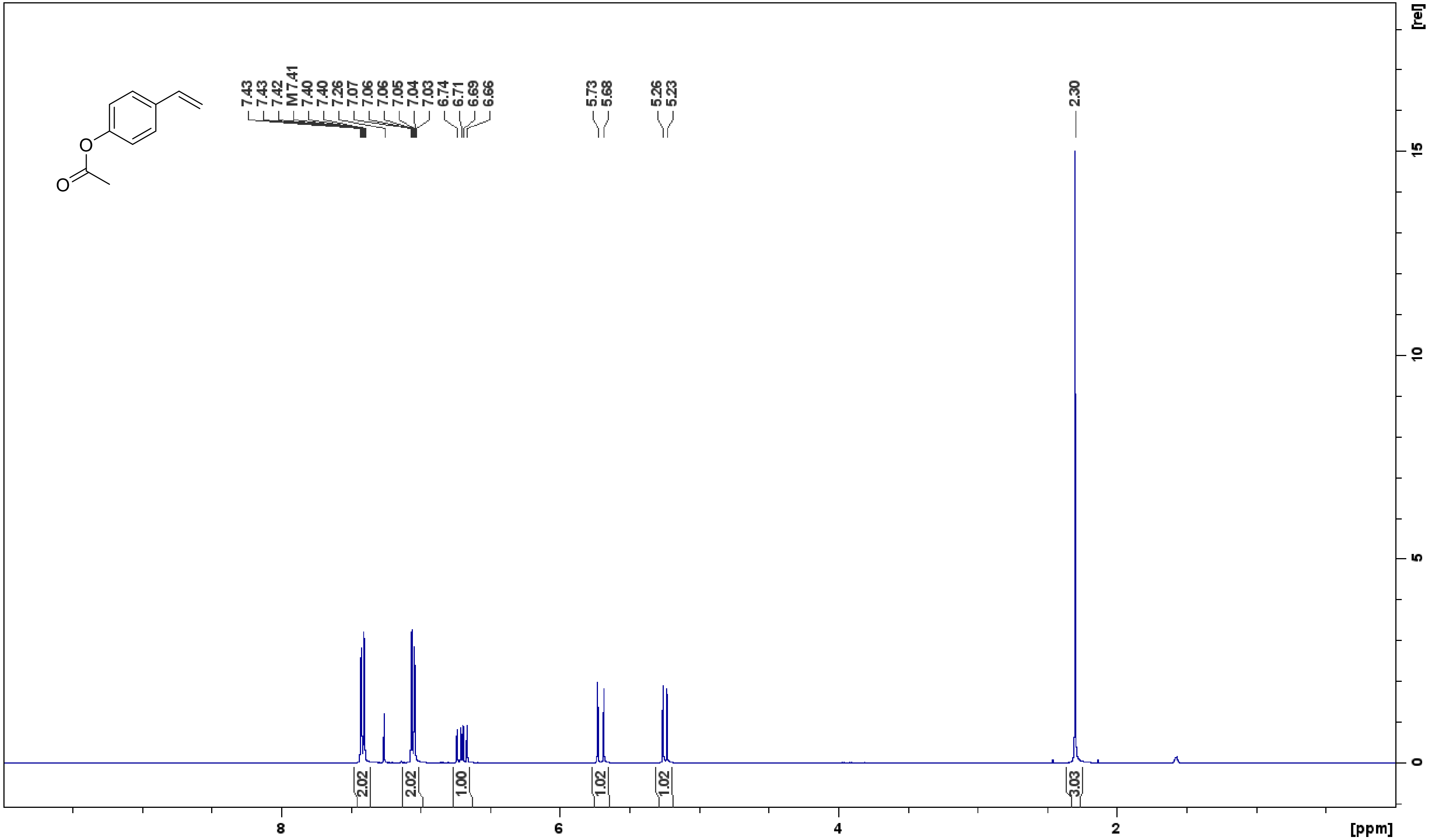

Figure SD 3. ^{1}H-NMR spectrum of 4-acetoxystyrene (AS) in $CDCl_3$ at 25 °C. Non-integrated peaks from residual NMR solvent (7.26 ppm) and moisture (1.56 ppm).

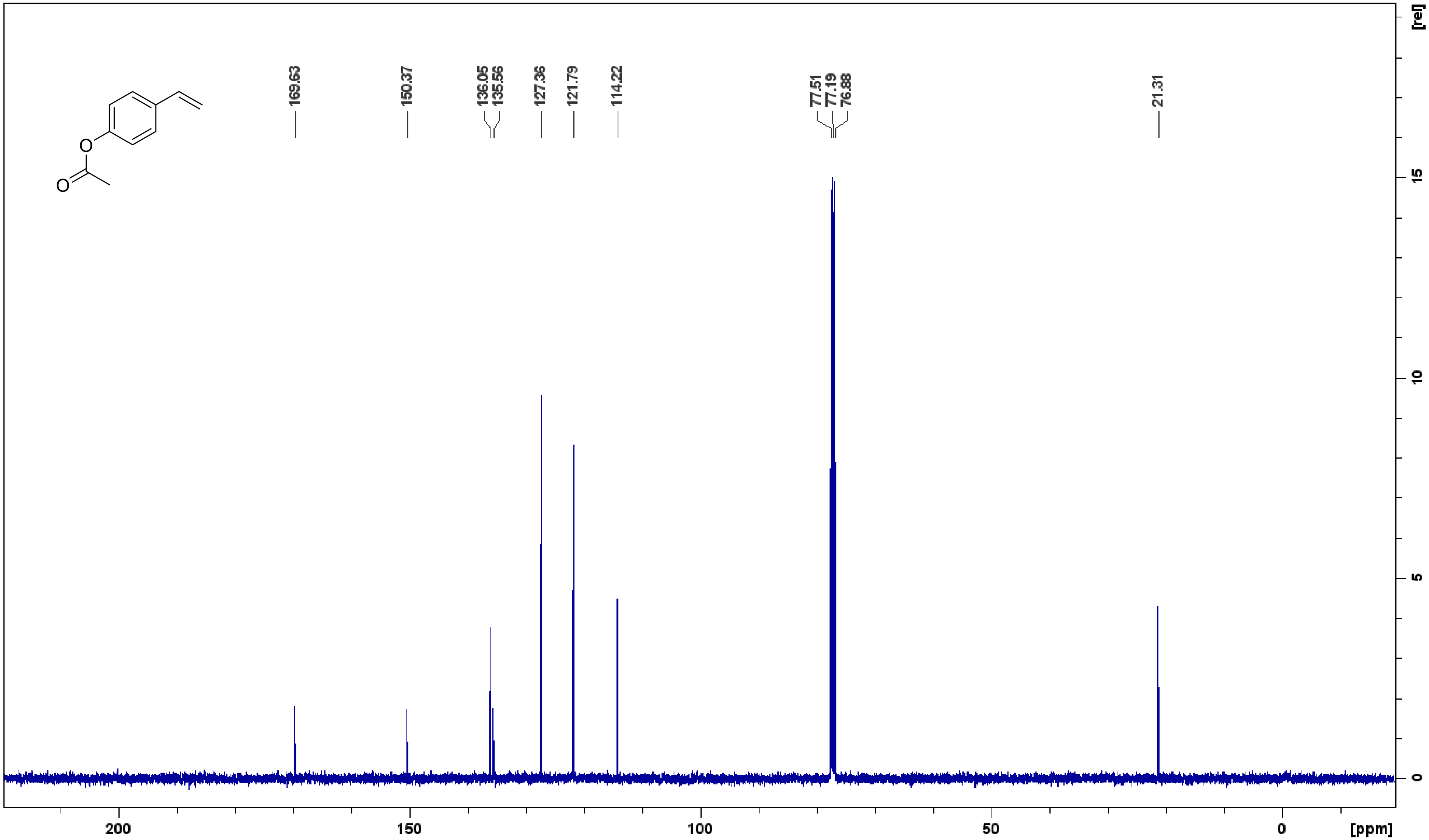

Figure SD 4. ^{13}C-NMR spectrum of 4-acetoxystyrene (AS) in $CDCl_3$ at 25 °C.

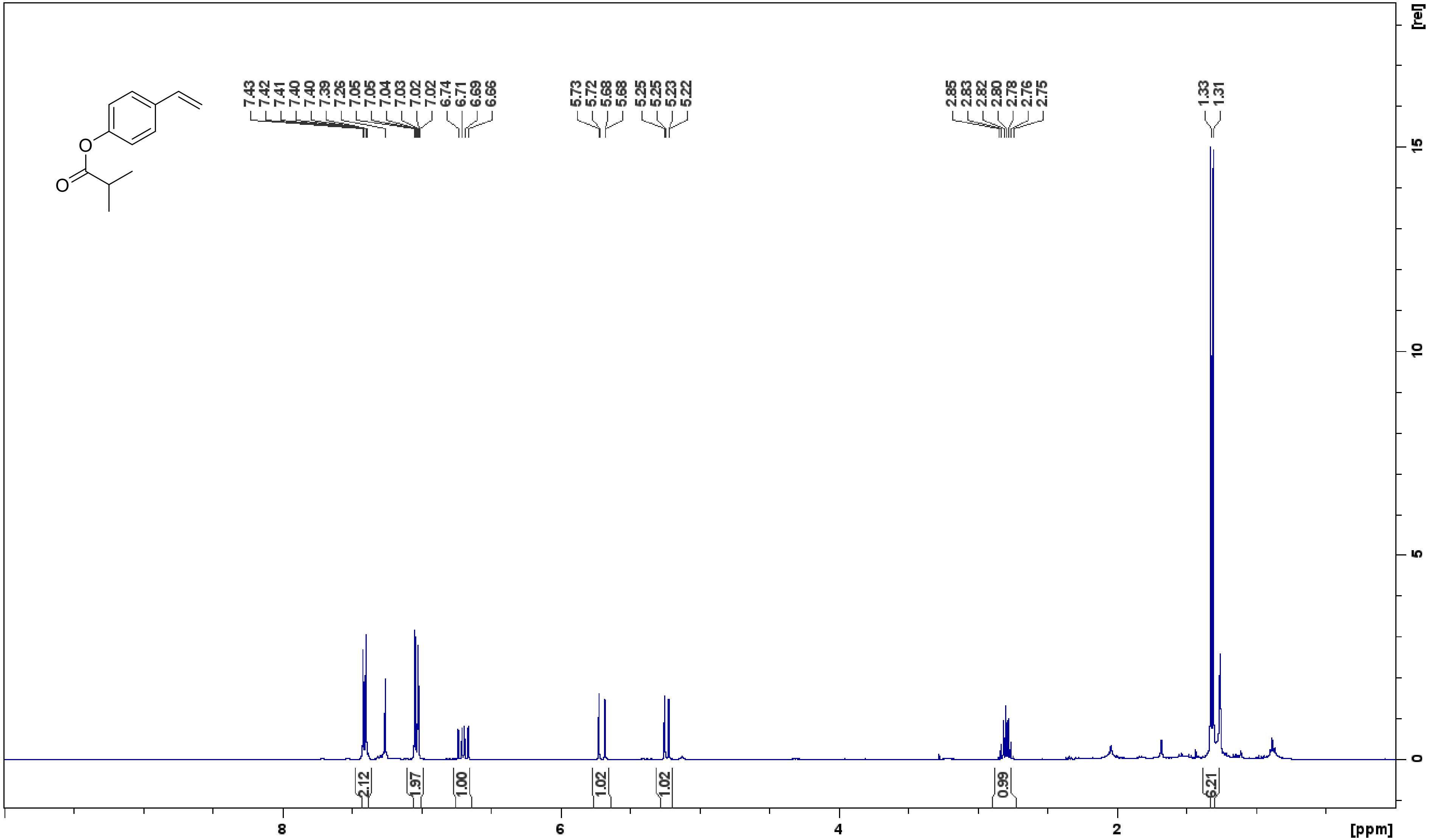

Figure SD 5. ^{1}H-NMR spectrum of 4-isobutanoyloxystyrene (IBS) in $CDCl_3$ at 25 °C. impurities: grease, CPME.

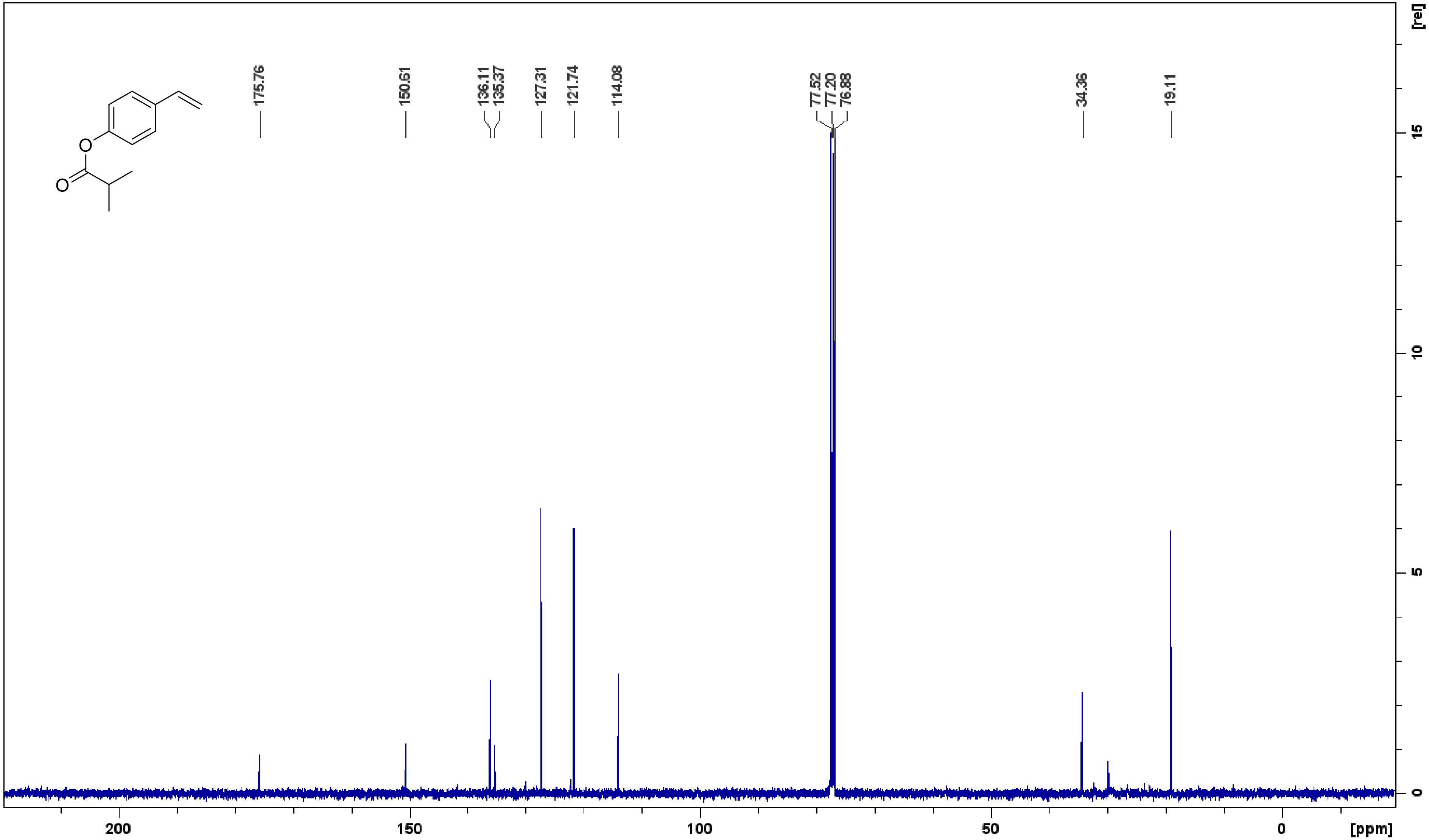

Figure SD 6. ^{13}C-NMR spectrum of 4-isobutanoyloxystyrene (IBS) in $CDCl_3$ at 25 °C. impurity: grease.

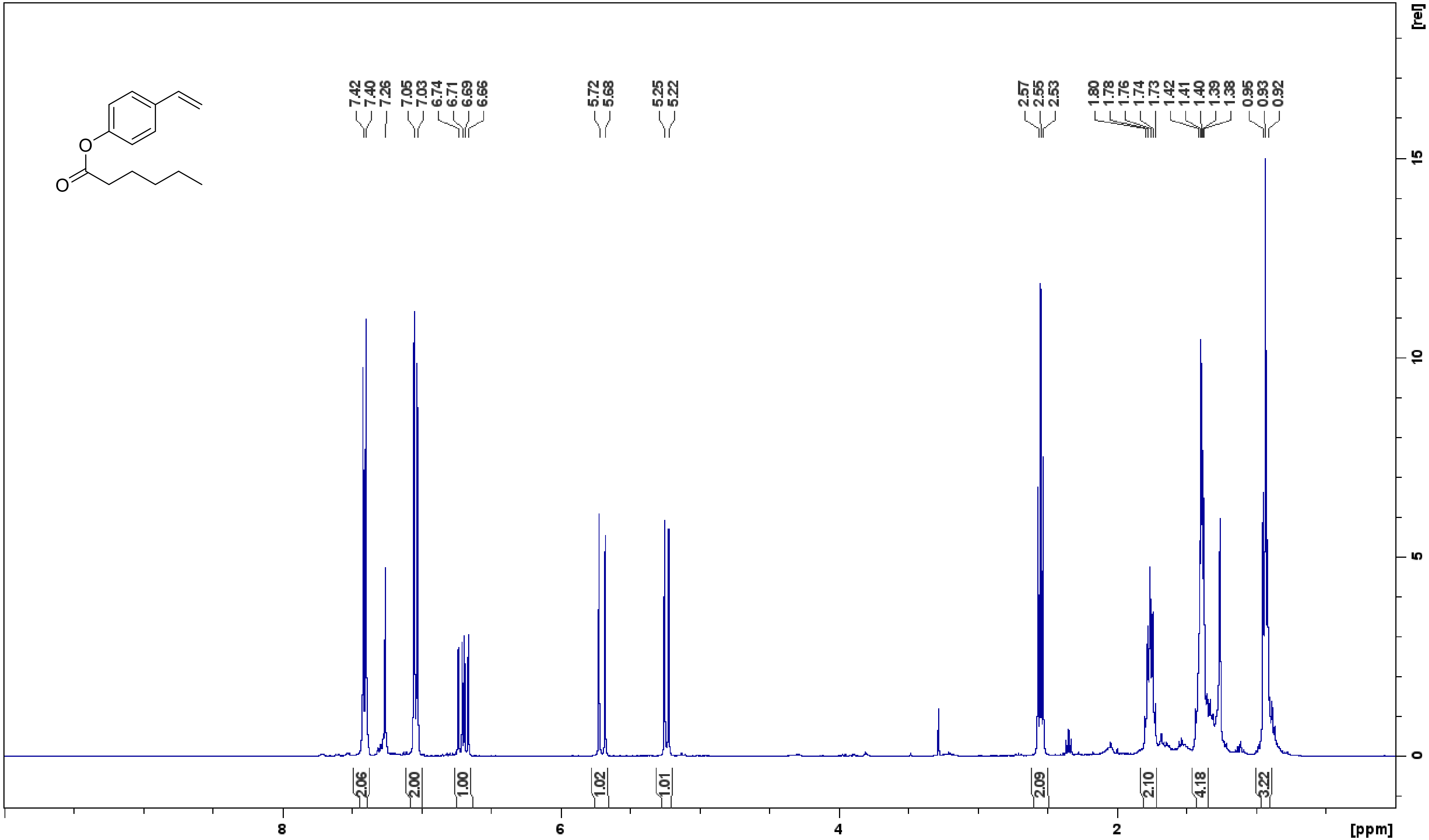

Figure SD 7. ^{1}H-NMR spectrum of 4-hexanoyloxystyrene (HS) in $CDCl_3$ at 25 °C. impurities: grease, hexanoic acid, CPME.

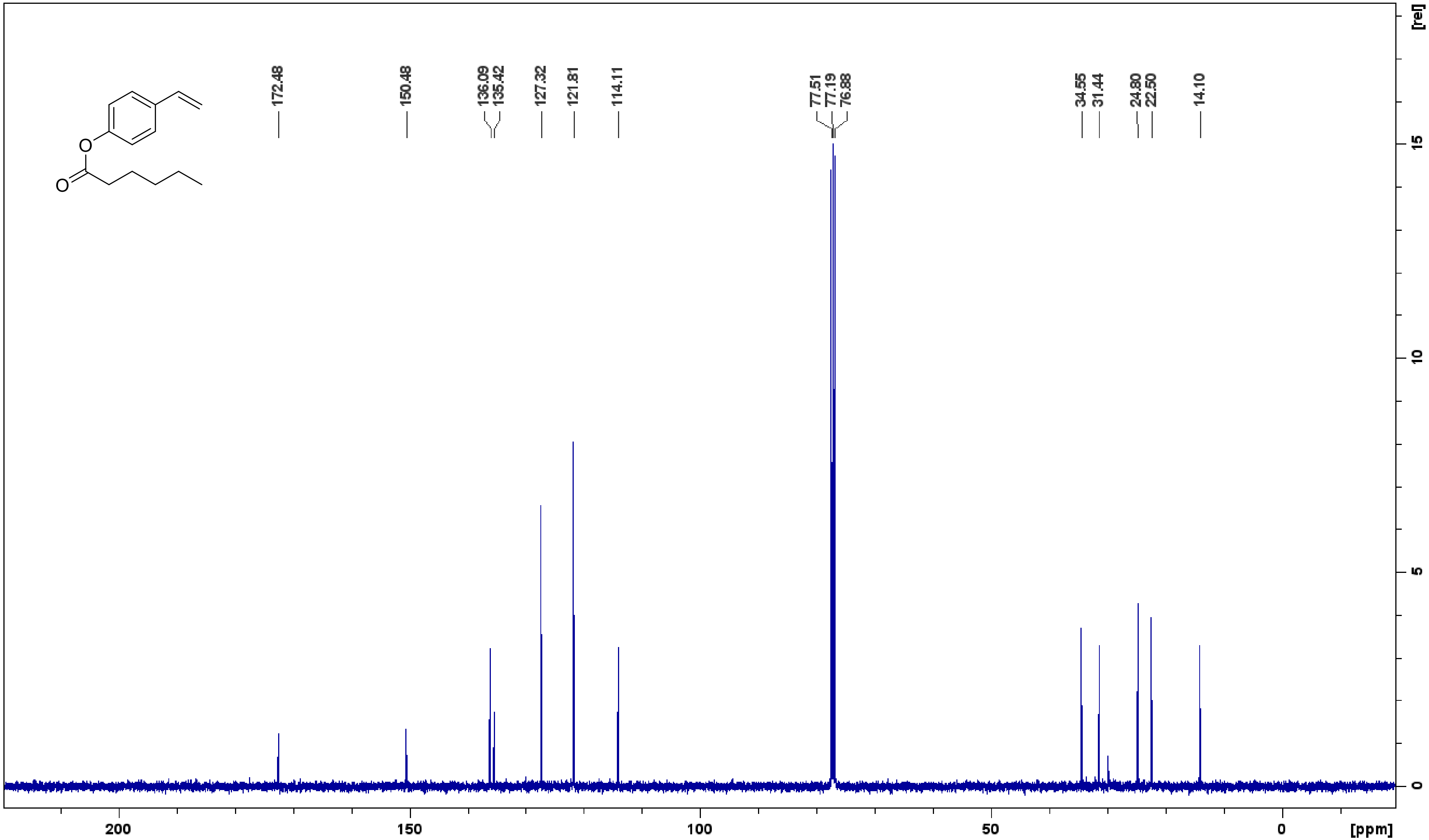

Figure SD 8. ^{13}C-NMR spectrum of 4-hexanoyloxystyrene (HS) in $CDCl_3$ at 25 °C.

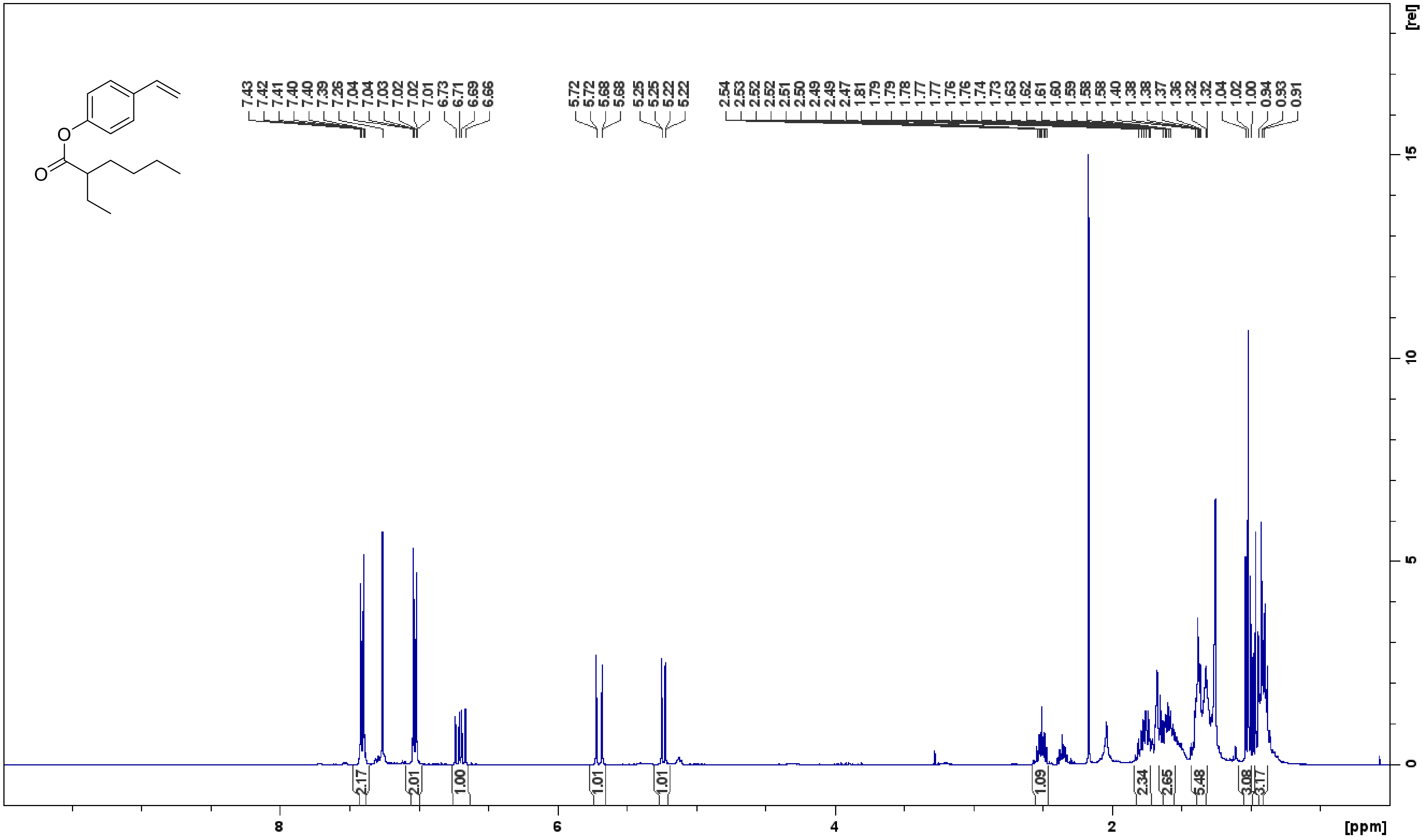

Figure SD 9. ^{1}H-NMR spectrum of 4-(2-ethyl-hexanoyloxy)styrene (EHS) in $CDCl_3$ at 25 °C. impurities: 2-ethylhexanoic acid, acetone, grease.

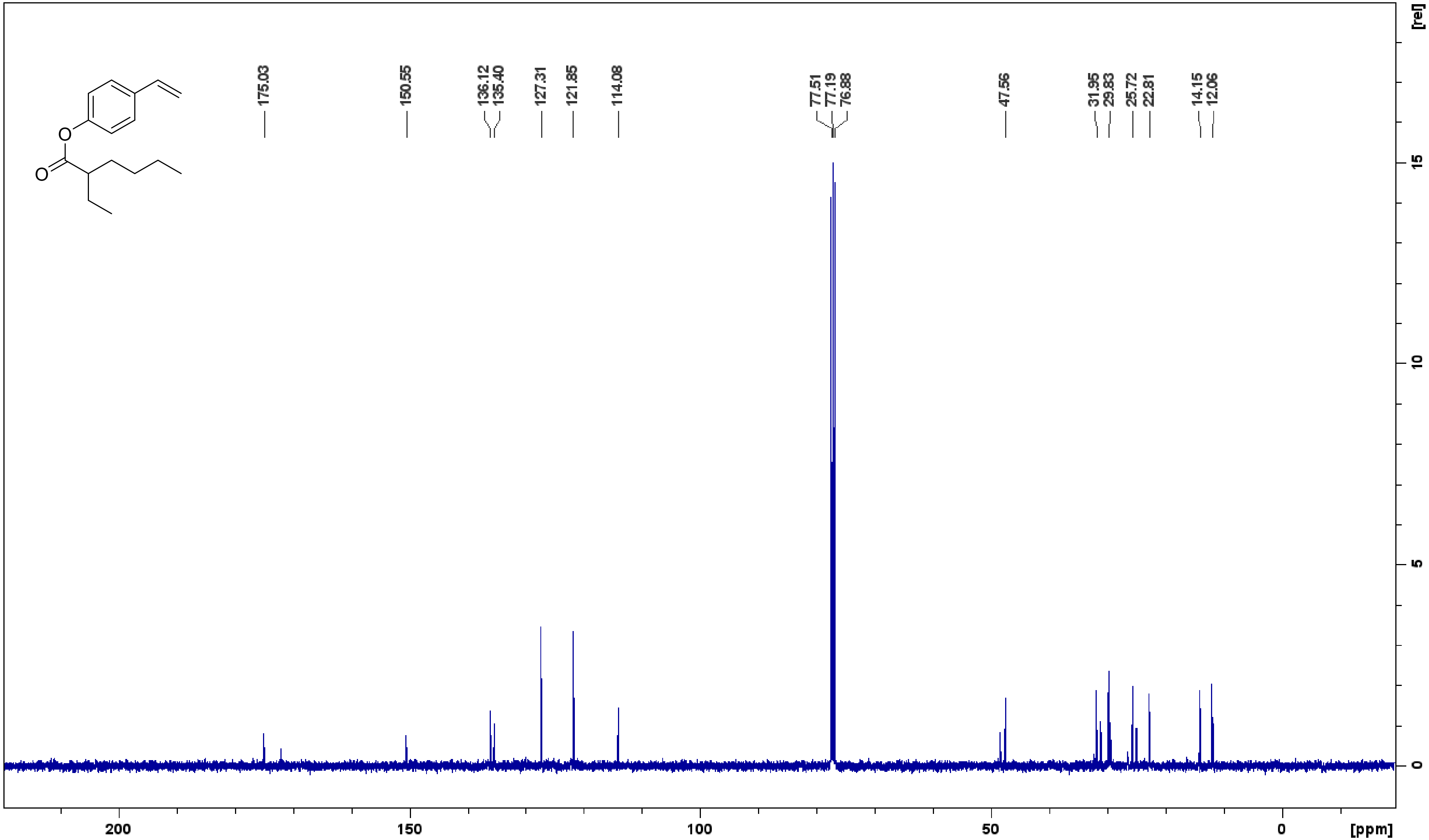

Figure SD 10. ^{13}C-NMR spectrum of 4-(2-ethyl-hexanoyloxy)styrene (EHS) in $CDCl_3$ at 25 °C. impurity: 2-ethylhexanoic acid, grease.

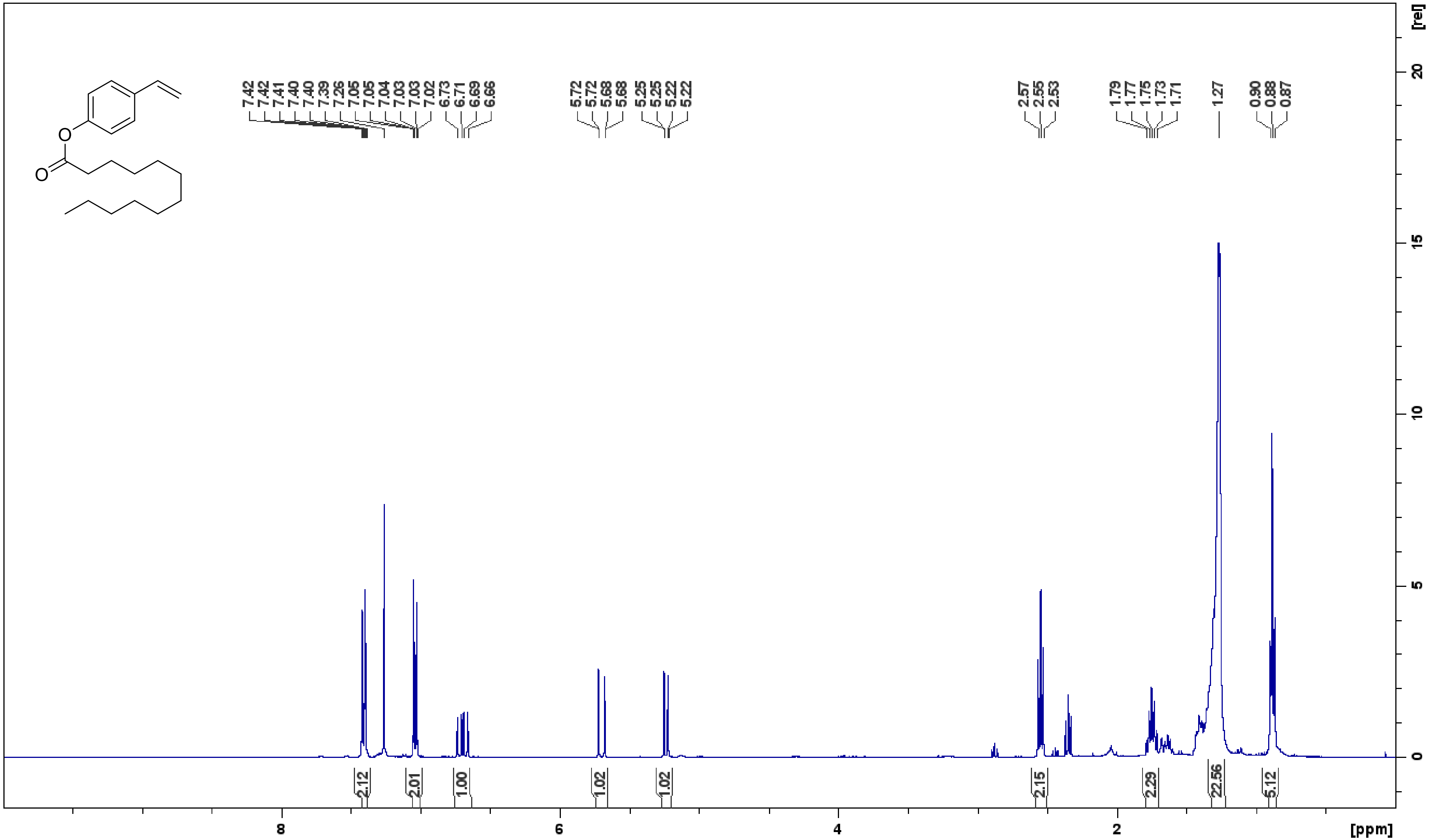

Figure SD 11. ^{1}H-NMR spectrum of 4-lauroyloxystyrene (LS) in $CDCl_3$ at 25 °C. impurities: lauric acid, grease.

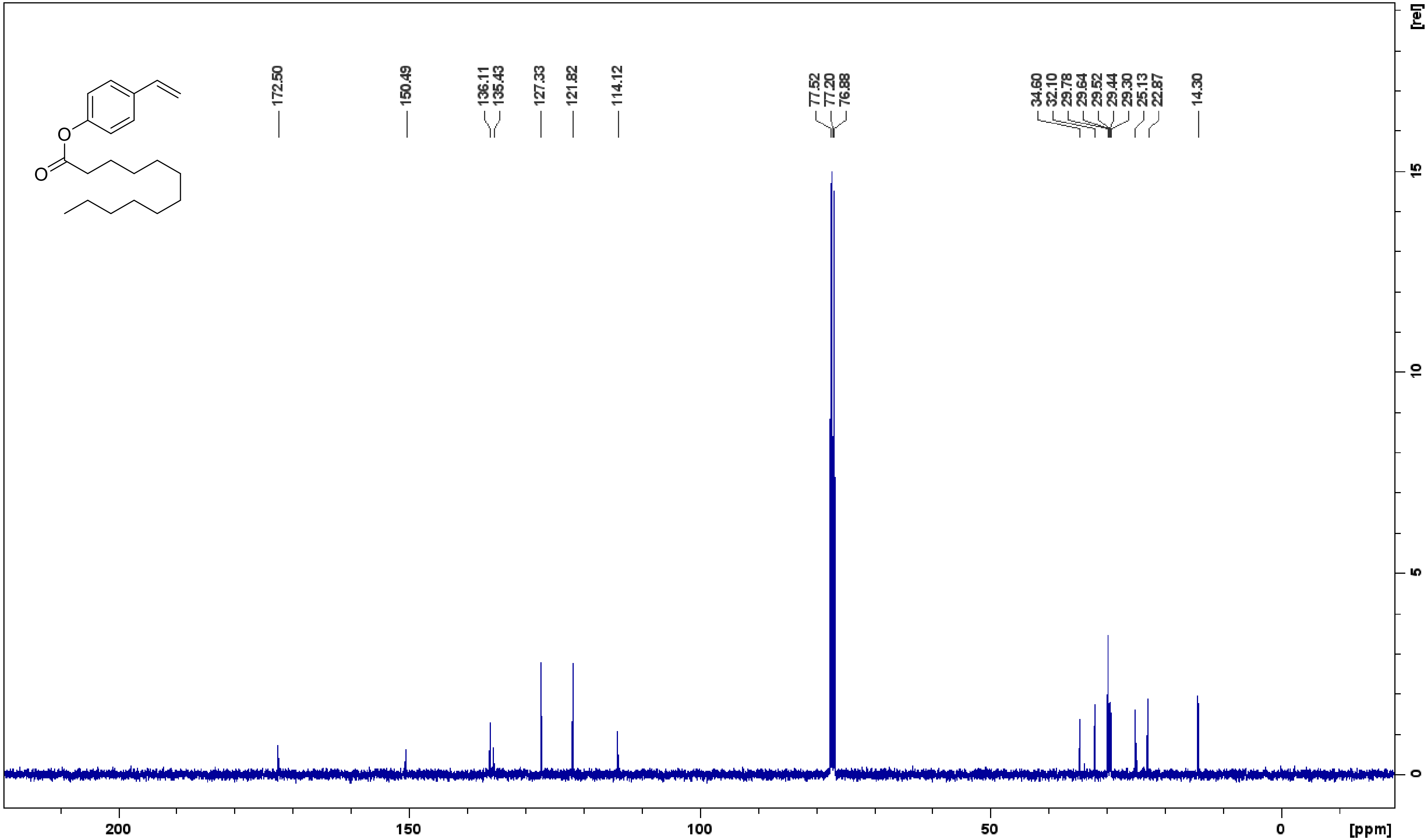

Figure S 12. ^{13}C-NMR spectrum of 4-lauroyloxystyrene (LS) in $CDCl_3$ at 25 °C. impurities: grease.

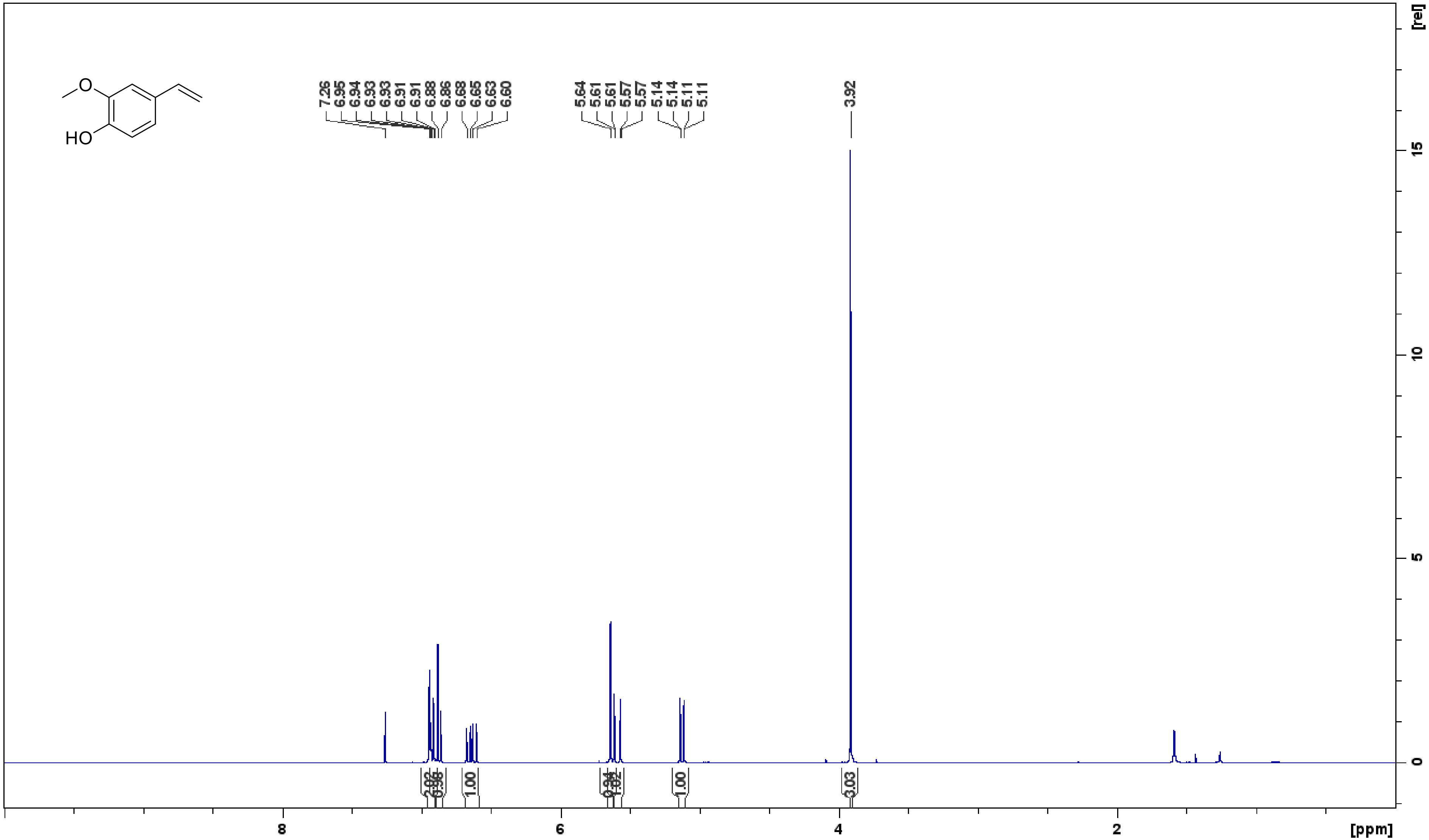

Figure SD 13. ^{1}H-NMR spectrum of 4-vinylguaiacol (4VG) in $CDCl_3$ at 25 °C. Non-integrated peaks arise from residual NMR solvent (7.26 ppm) and moisture (1.56 ppm). minor impurity: grease.

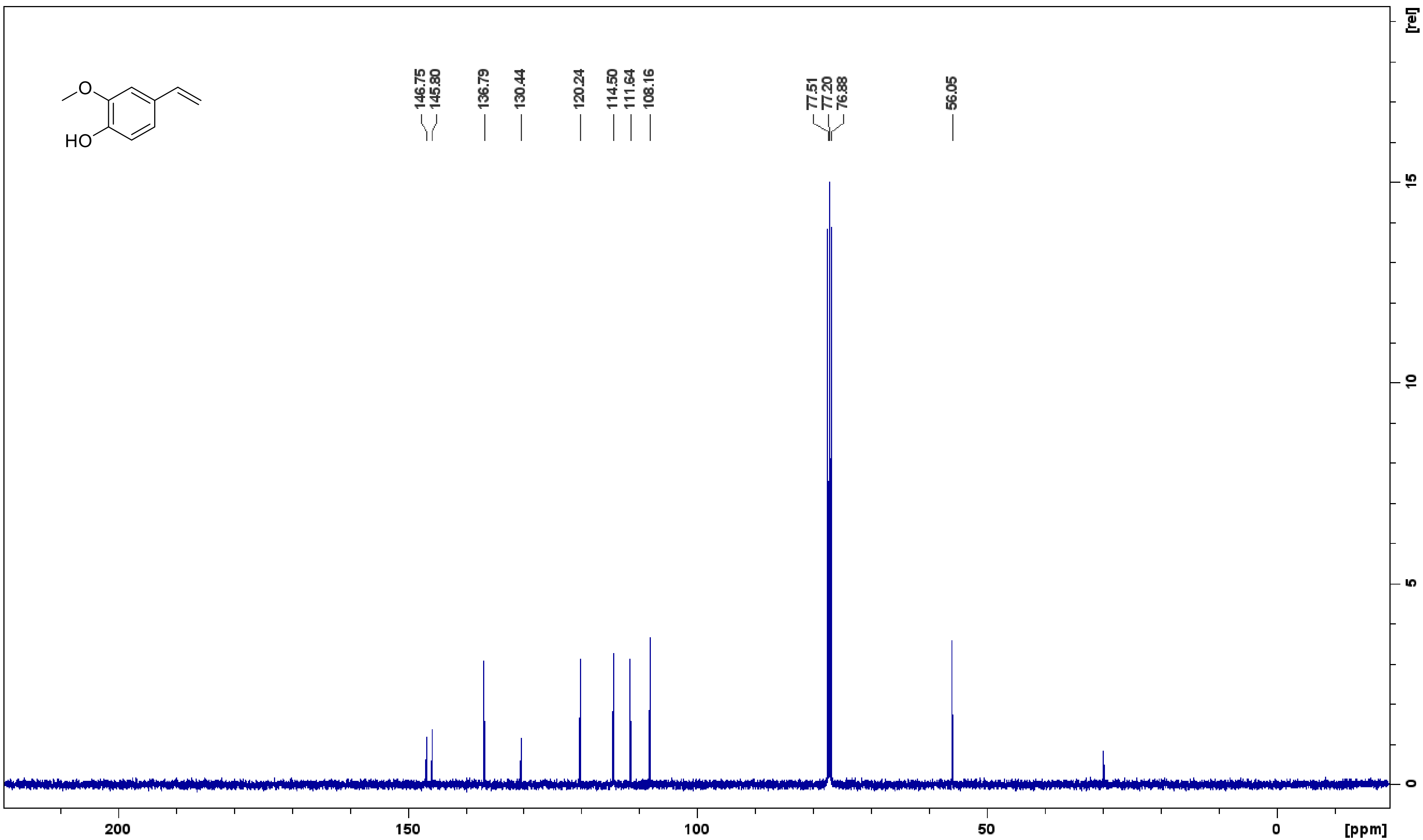

Figure SD 14. ^{13}C-NMR spectrum of 4-vinylguaiacol (4VG) in $CDCl_3$ at 25 °C. minor impurity: grease.

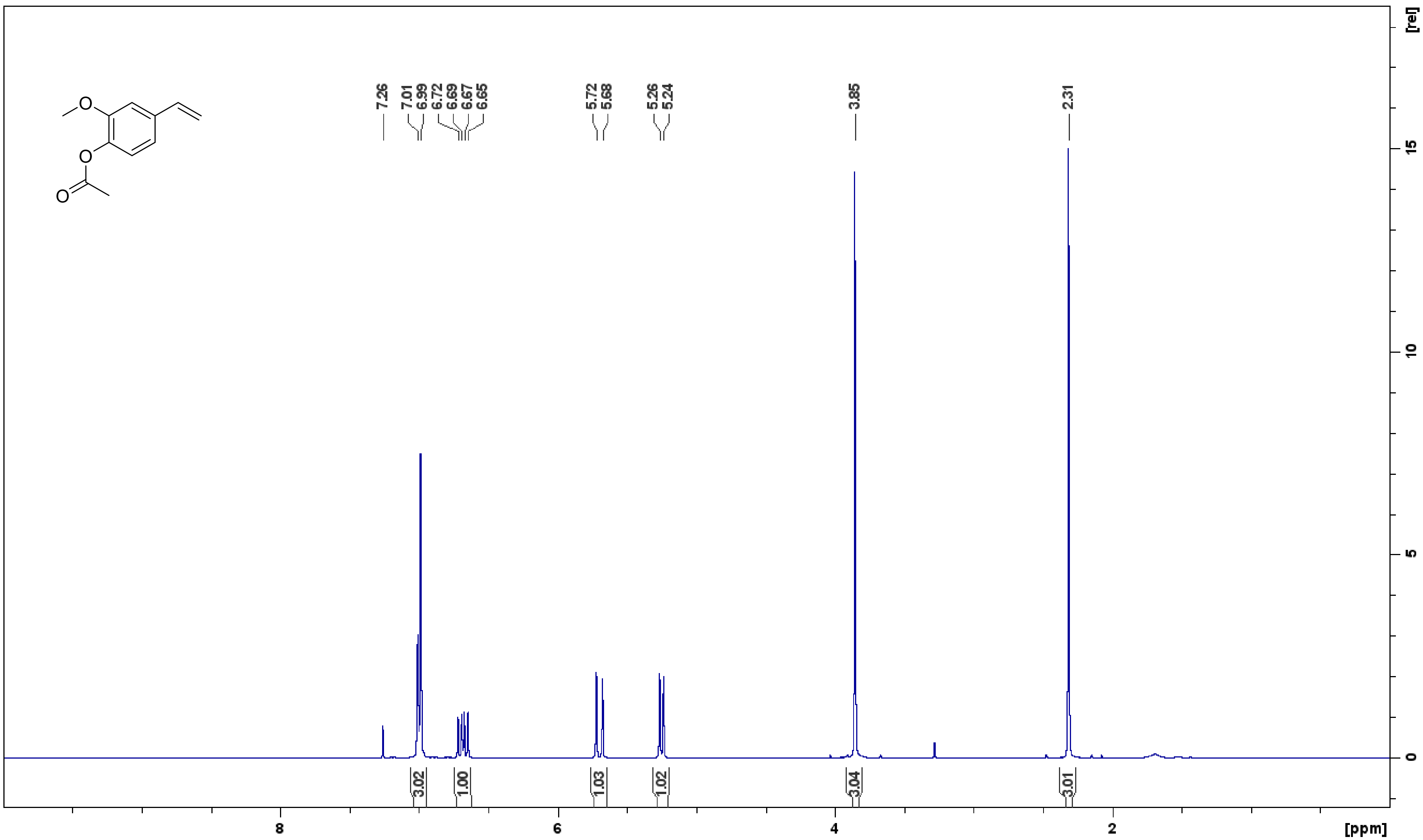

Figure SD 15. ^{1}H-NMR spectrum of 4-acetoxy-3-methoxystyrene (AMS) in $CDCl_3$ at 25 °C. impurity: CPME.

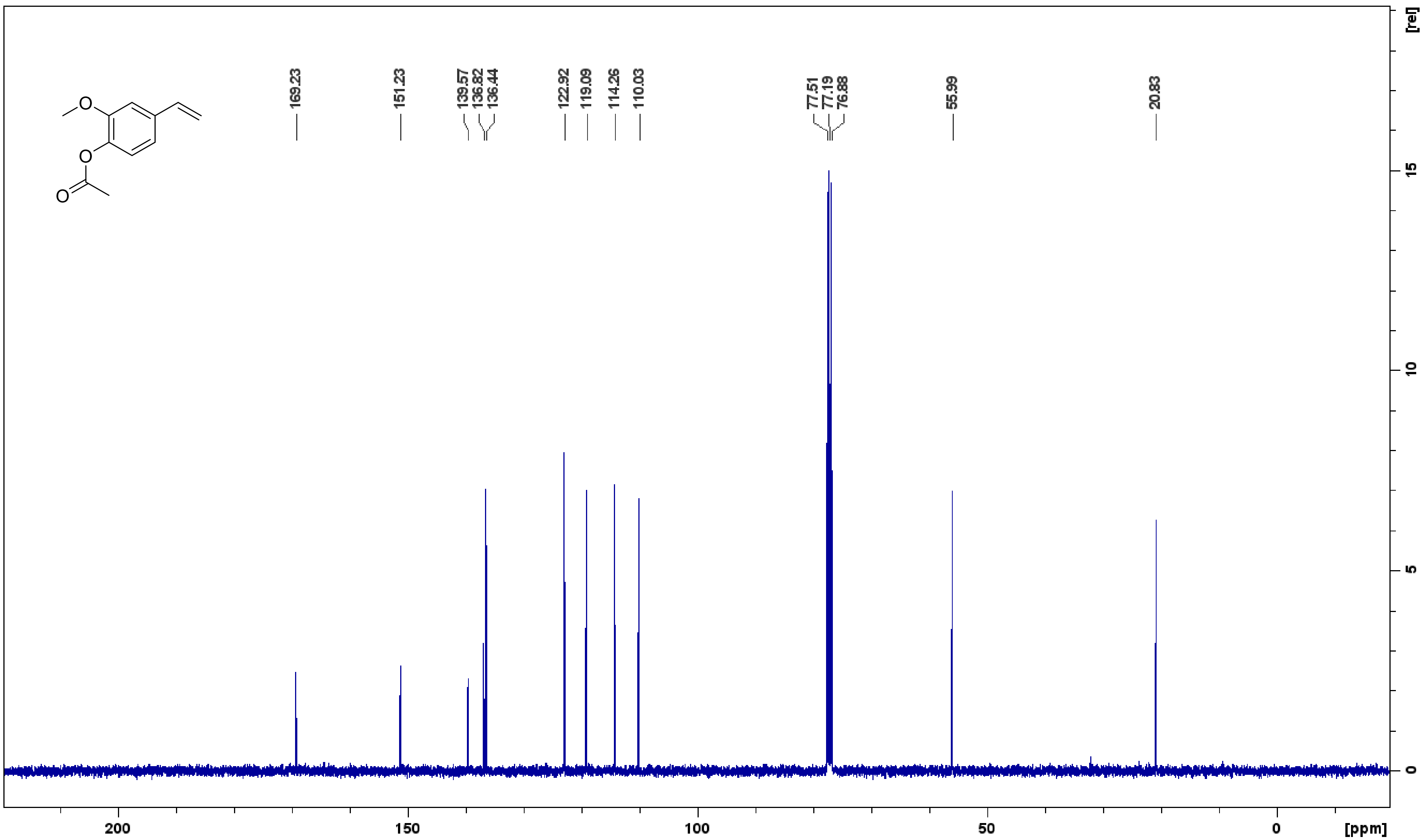

Figure SD 16. ^{13}C-NMR spectrum of 4-acetoxy-3-methoxystyrene (AMS) in $CDCl_3$ at 25 °C.

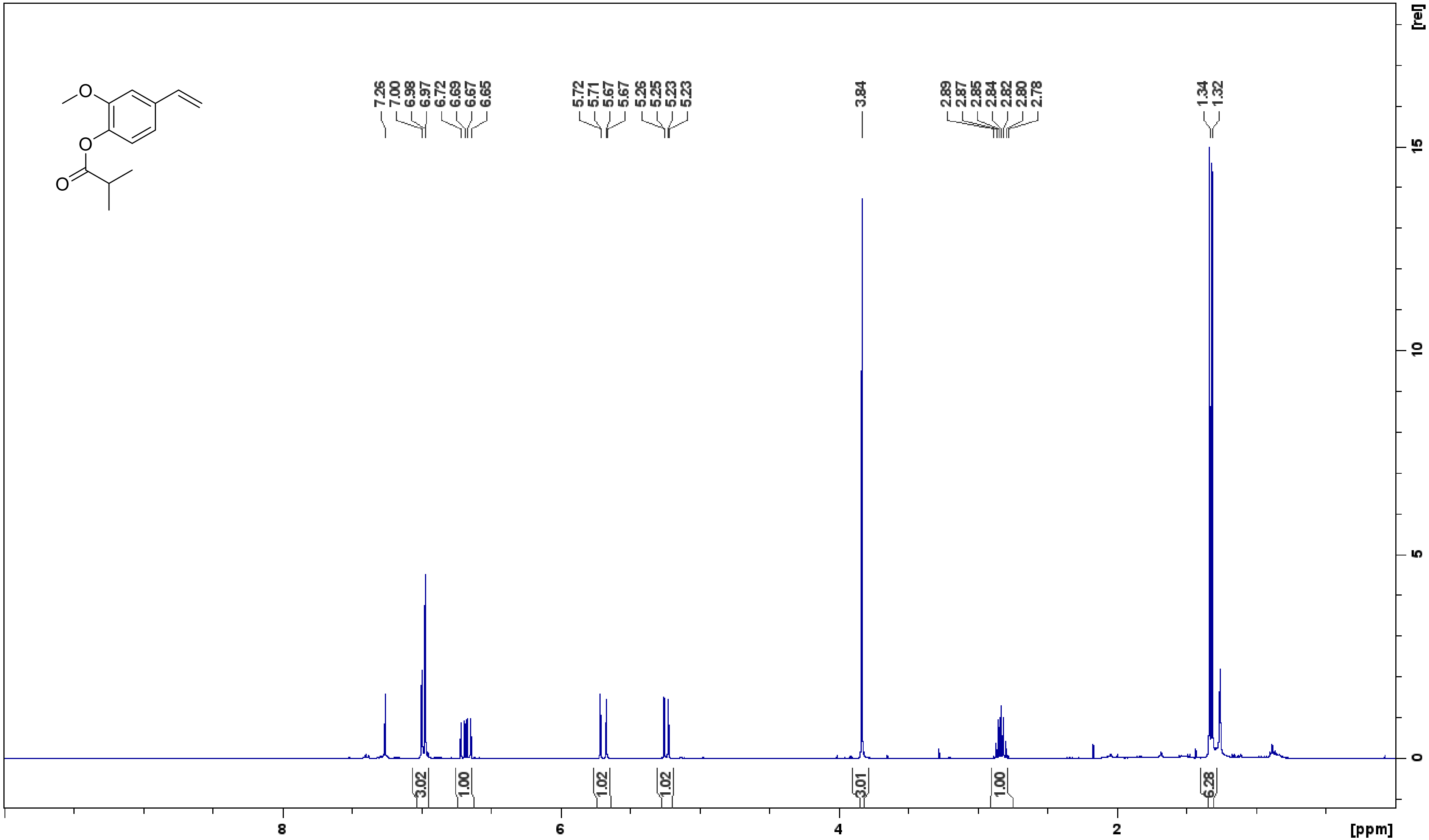

Figure SD 17. ^{1}H-NMR spectrum of 4-isobutanoyloxy-3-methoxystyrene (IBMS) in $CDCl_3$ at 25 °C. impurities: grease, CPME, acetone.

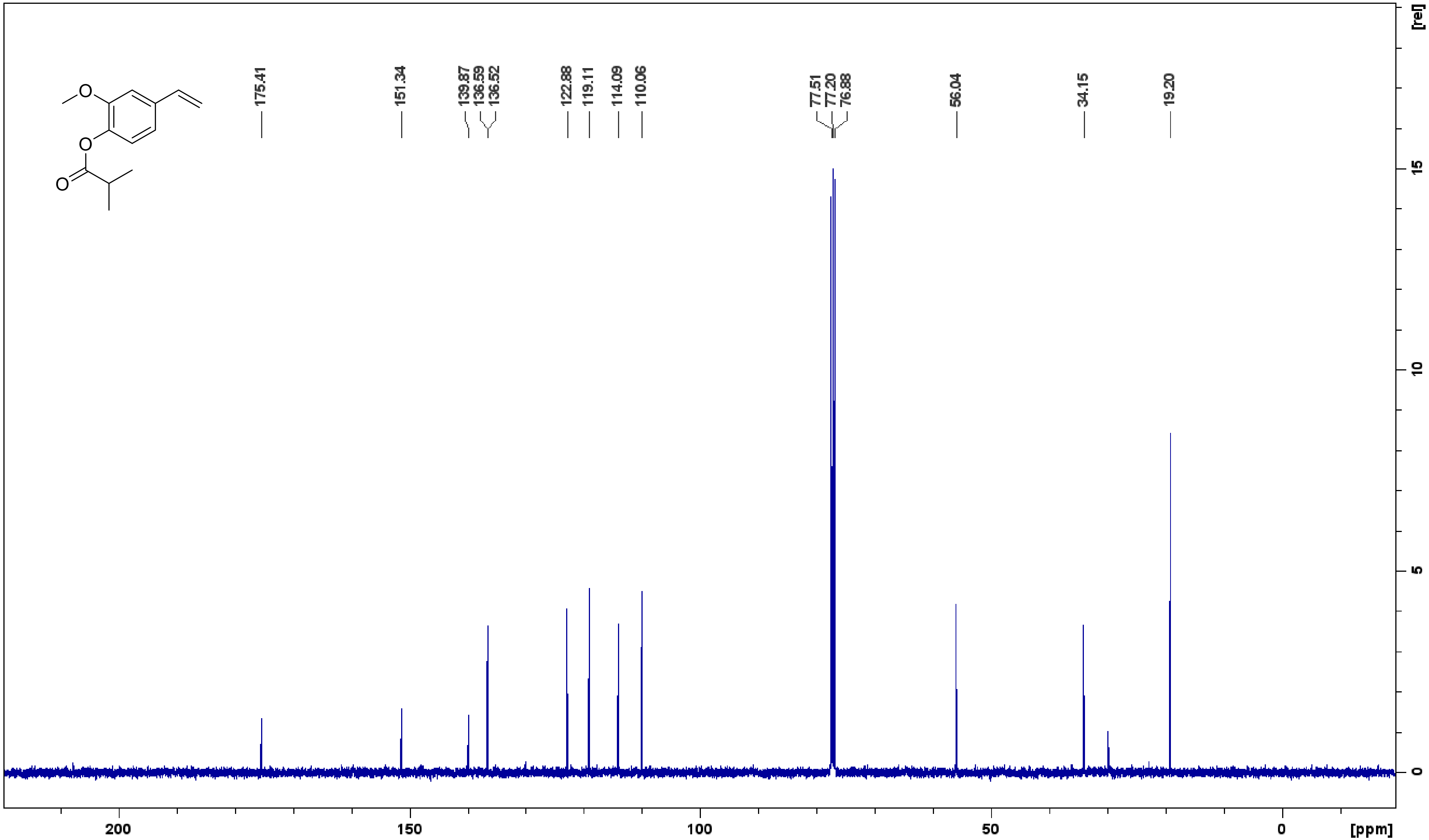

Figure SD 18. ^{13}C-NMR spectrum of 4-isobutanoyloxy-3-methoxystyrene (IBMS) in $CDCl_3$ at 25 °C.

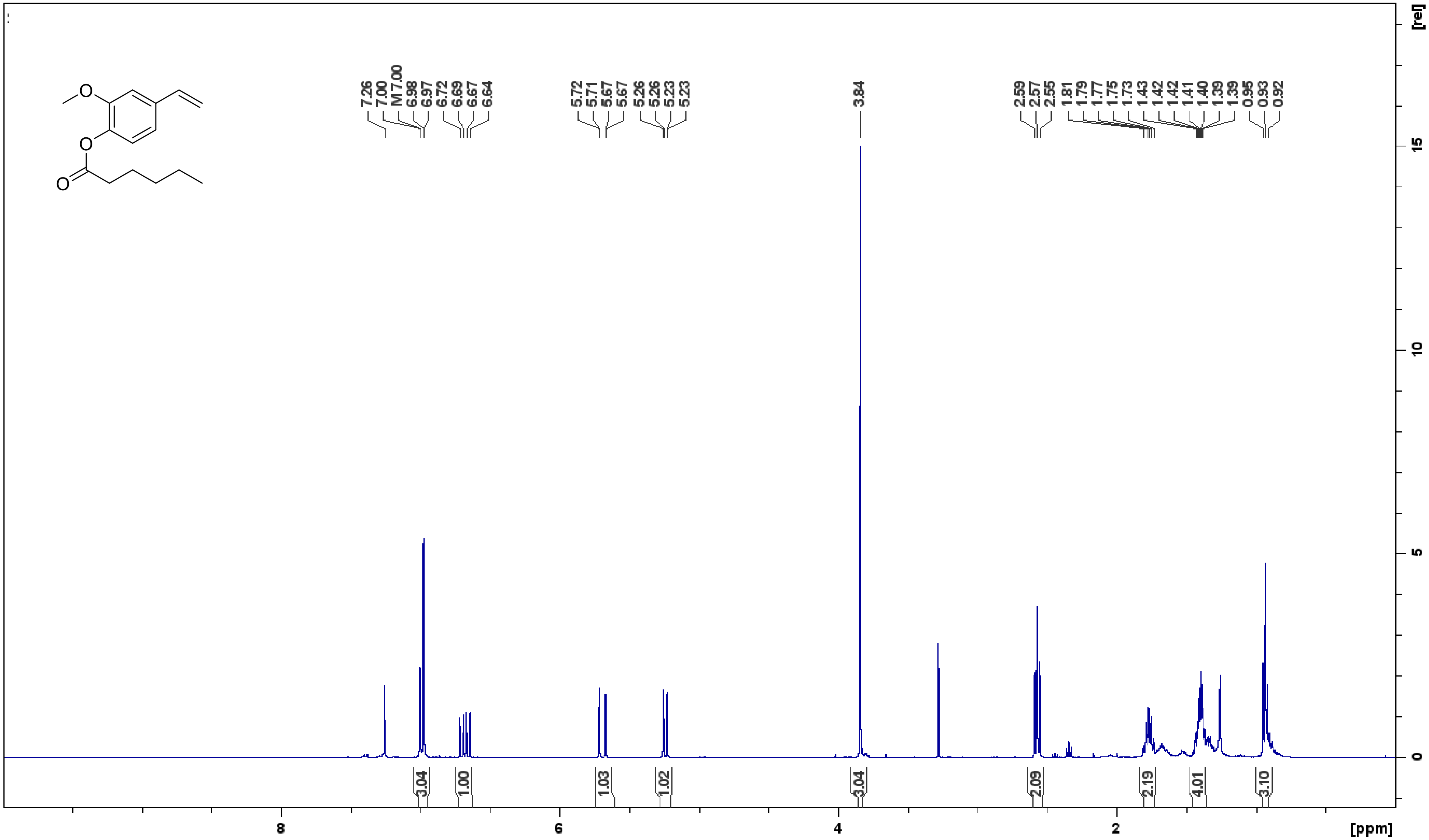

Figure SD 19. ^{1}H-NMR spectrum of 4-hexanoyloxy-3-methoxystyrene (HMS) in $CDCl_3$ at 25 °C. impurities: CPME, grease, hexanoic acid.

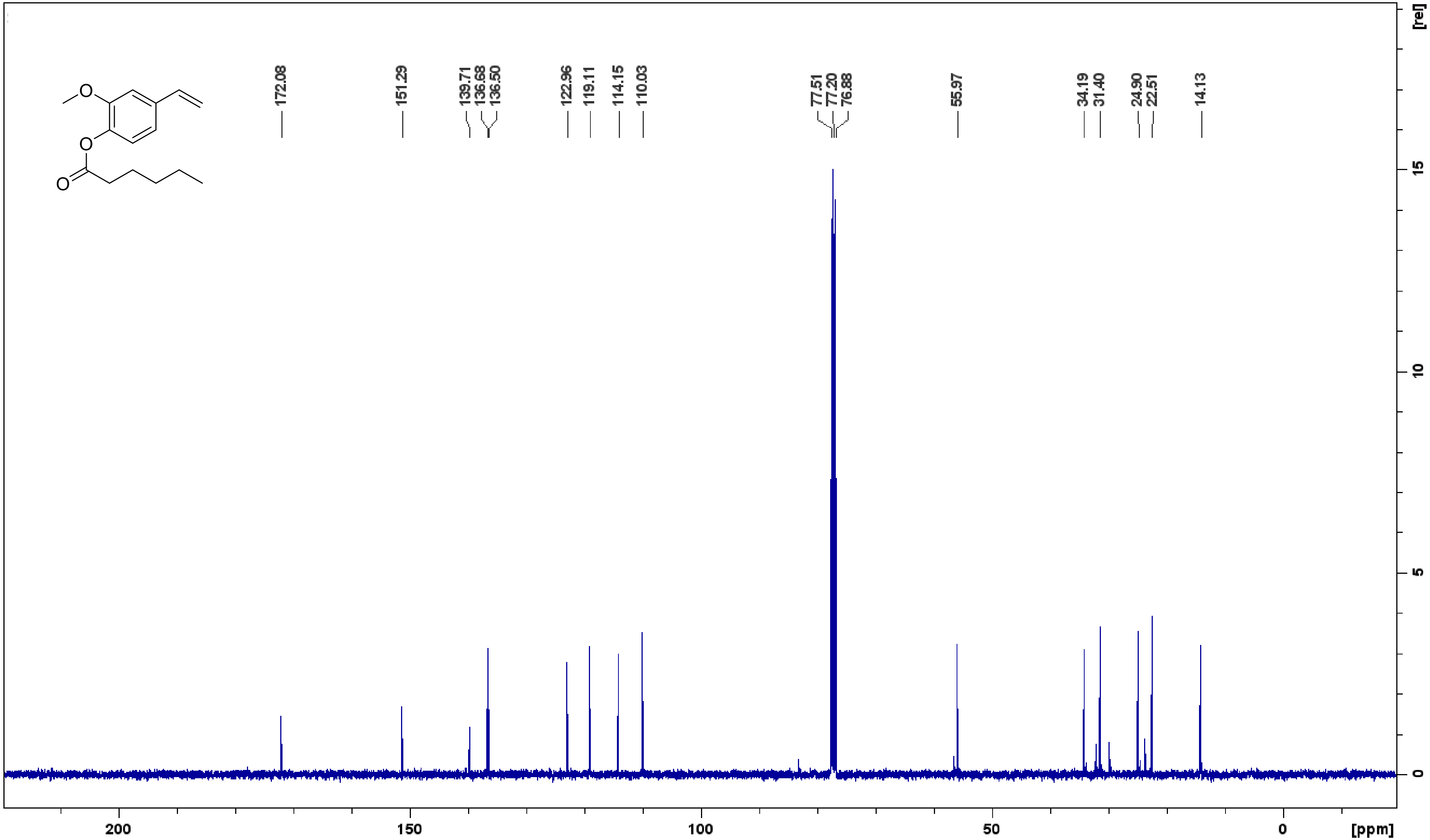

Figure SD 20. ^{13}C-NMR spectrum of 4-hexanoyloxy-3-methoxystyrene (HMS) in $CDCl_3$ at 25 °C. impurities: CPME, grease.

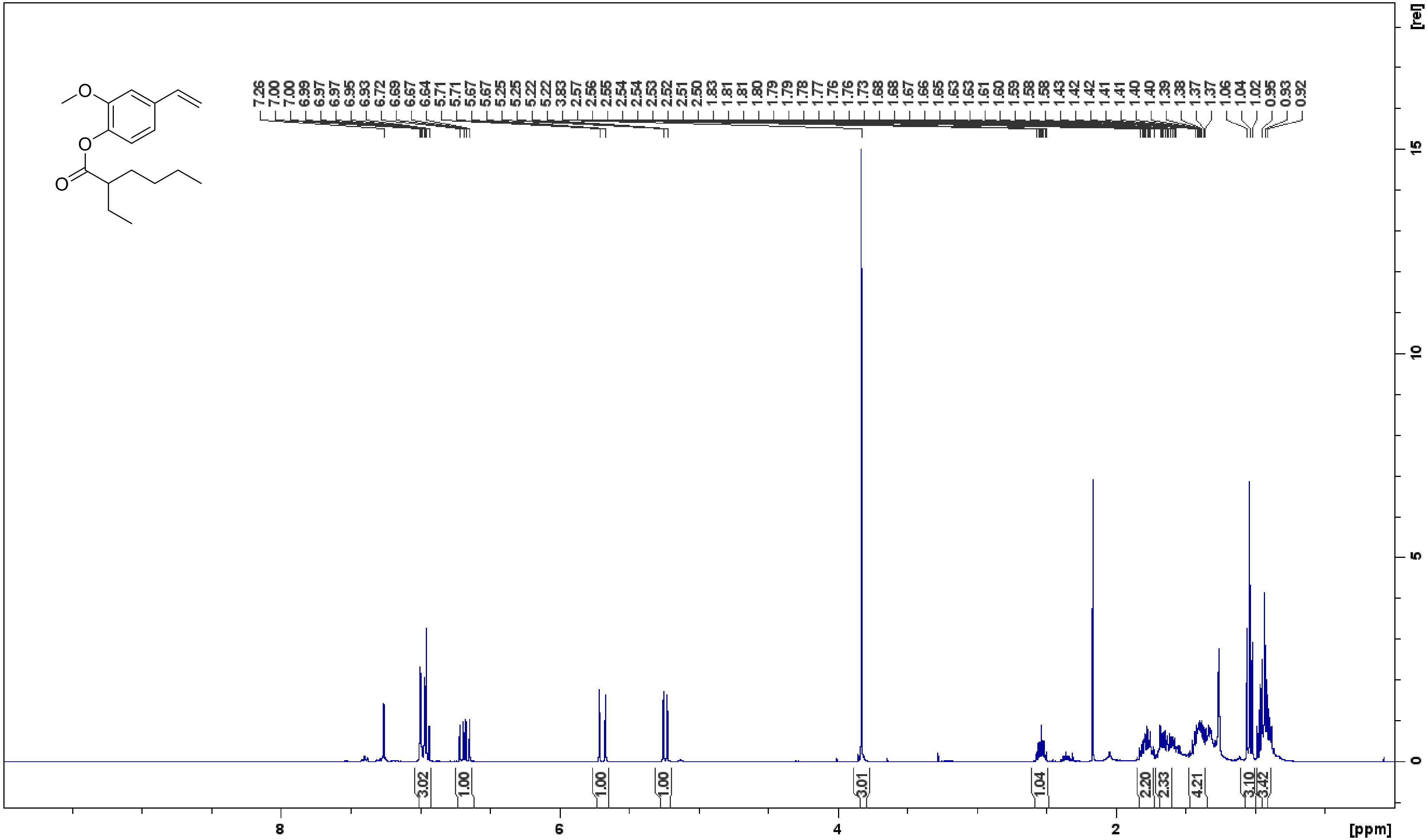

Figure SD 21. ^{1}H-NMR spectrum of 4-(2-ethyl-hexanoyloxy)-3-methoxystyrene (EHMS) in $CDCl_3$ at 25 °C. impurities: 2-ethylhexanoic acid, acetone, grease.

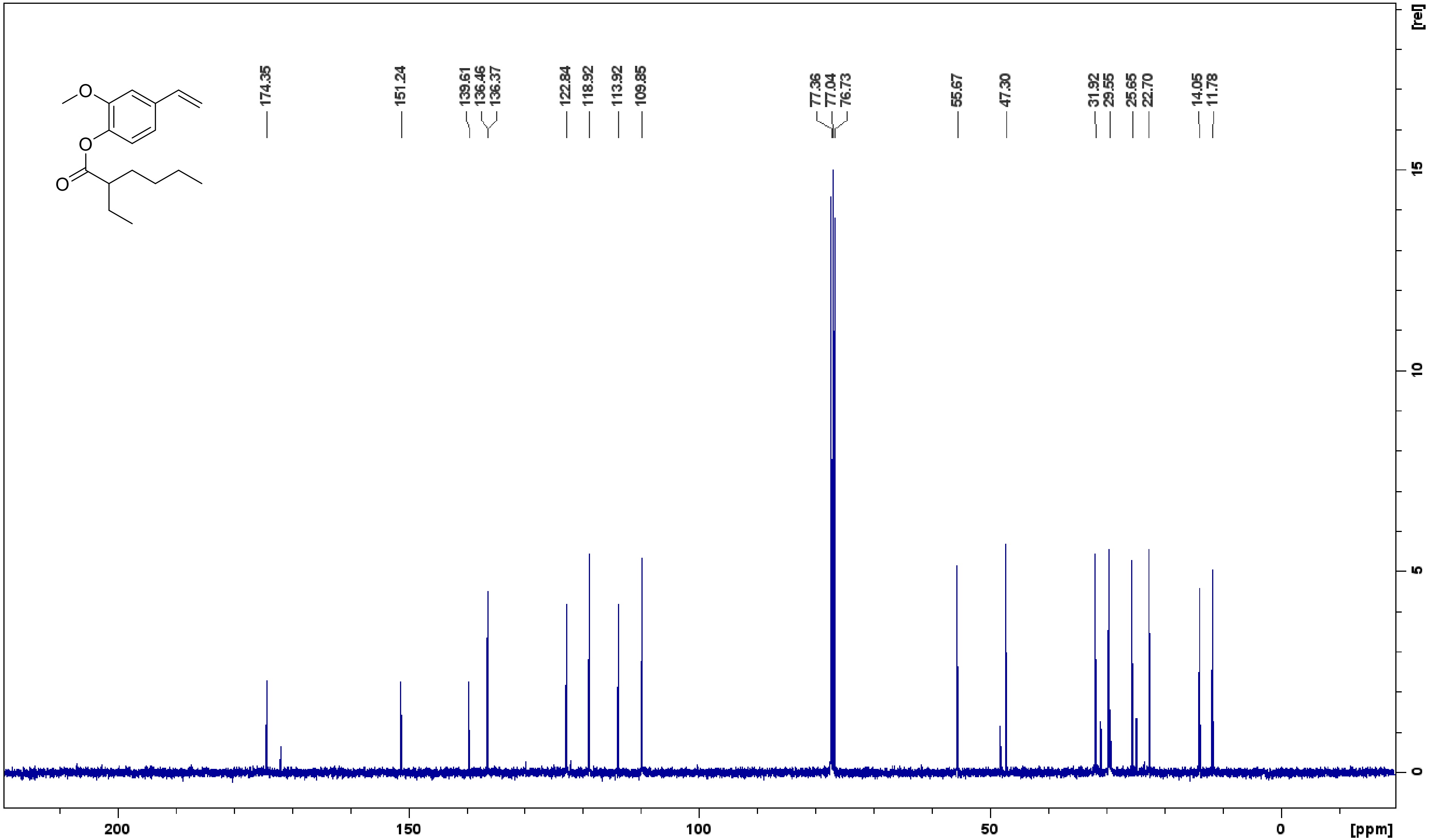

Figure SD 22. ^{13}C-NMR spectrum of 4-(2-ethyl-hexanoyloxy)-3-methoxystyrene (EHMS) in $CDCl_3$ at 25 °C. impurities: 2-ethylhexanoic acid, grease.

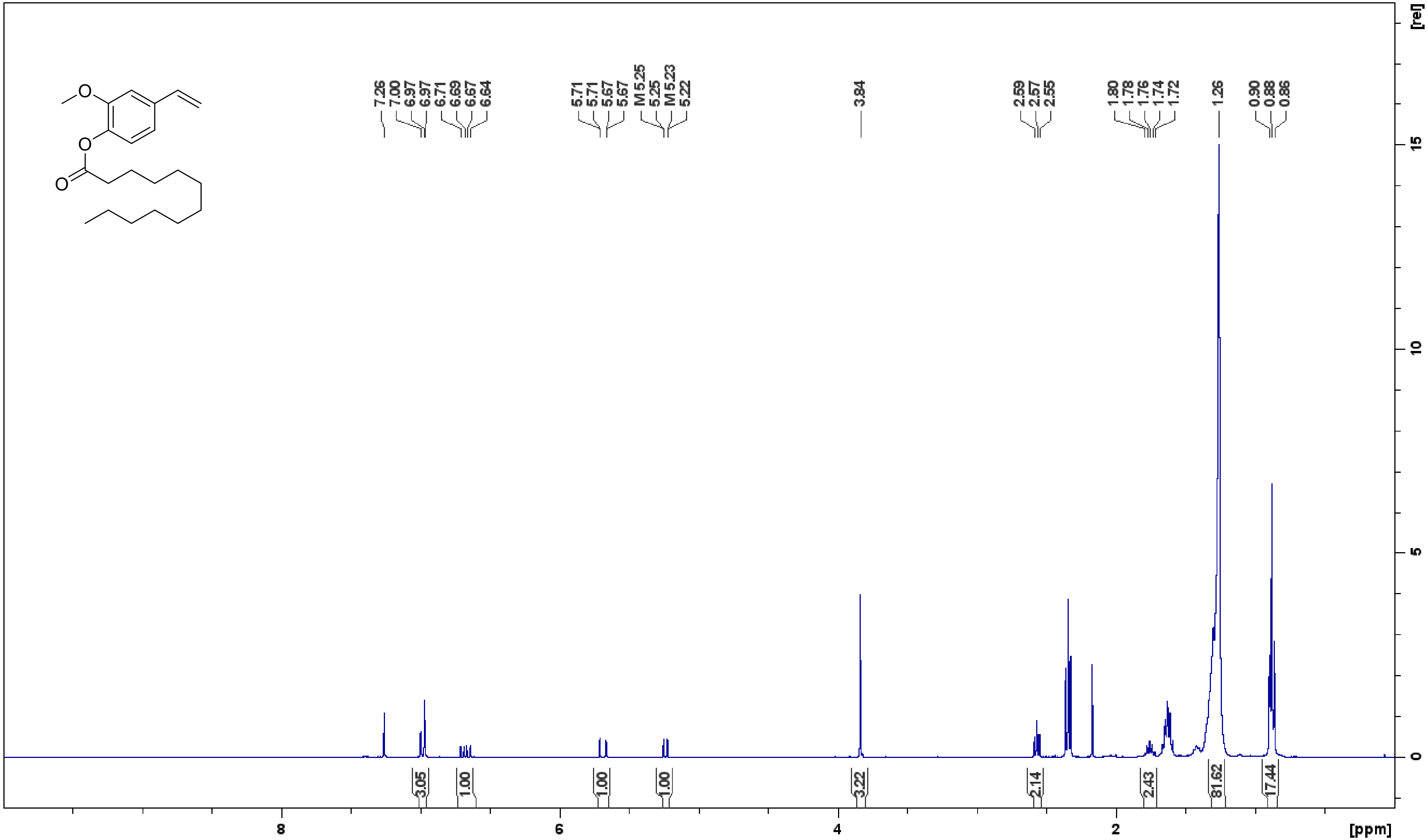

Figure SD 23. ^{1}H-NMR spectrum of 4-lauroyloxy-3-methoxystyrene (LMS) in $CDCl_3$ at 25 °C. impurities: lauric acid, acetone, grease.

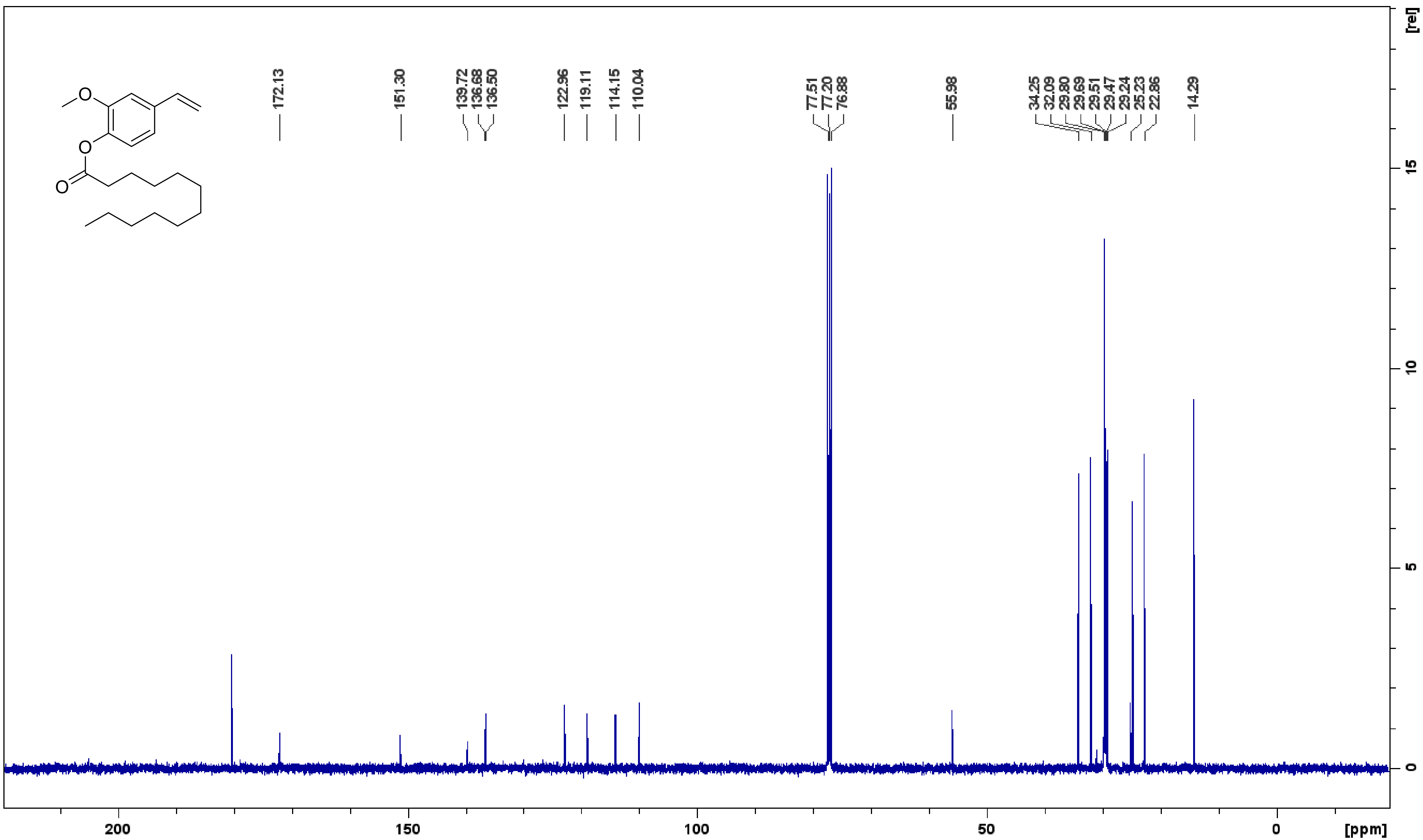

Figure SD 24. ^{13}C-NMR spectrum of 4-lauroyloxy-3-methoxystyrene (LMS) in $CDCl_3$ at 25 °C. impurities: lauric acid, grease.

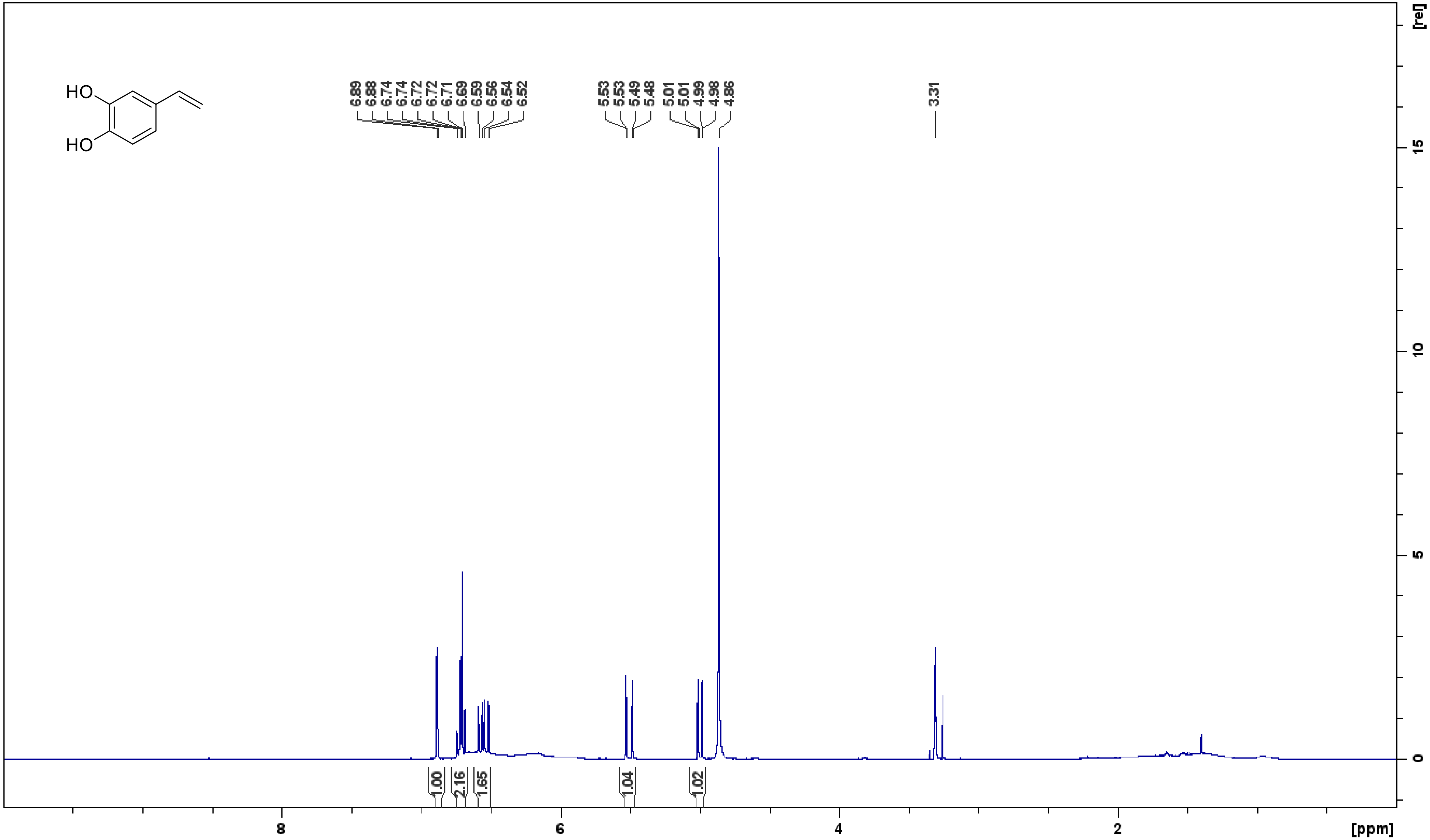

Figure SD 25. ^{1}H-NMR spectrum of 4-vinylcatechol (4VC) in CD_3OD at 25 °C. impurity: CPME.

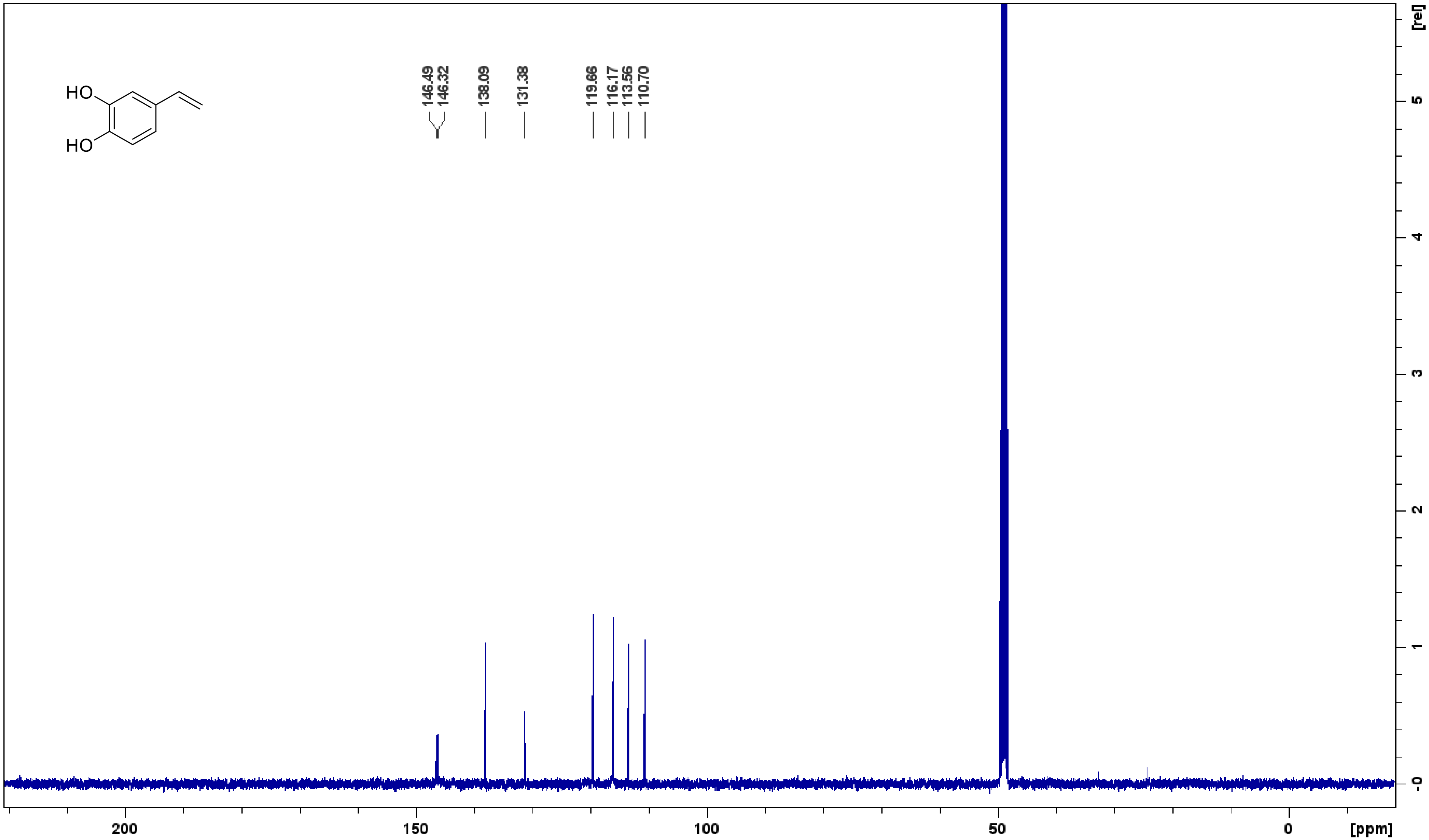

Figure SD 26. ^{13}C-NMR spectrum of 4-vinylcatechol (4VC) in CD_3OD at 25 °C.

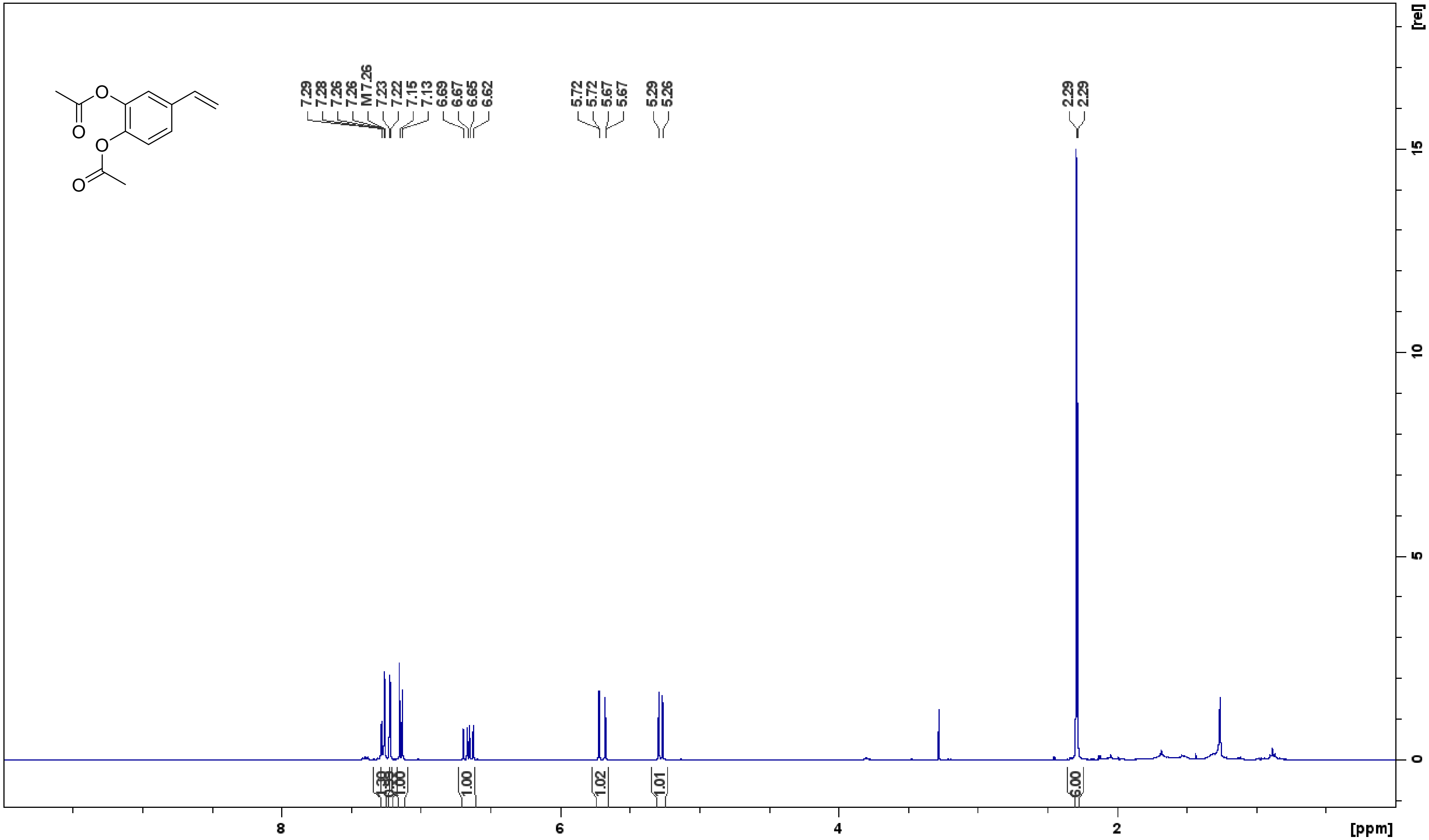

Figure SD 27. ^{1}H-NMR spectrum of 3,4-diacetoxystyrene (DAS) in $CDCl_3$ at 25 °C. impurities: grease, CPME.

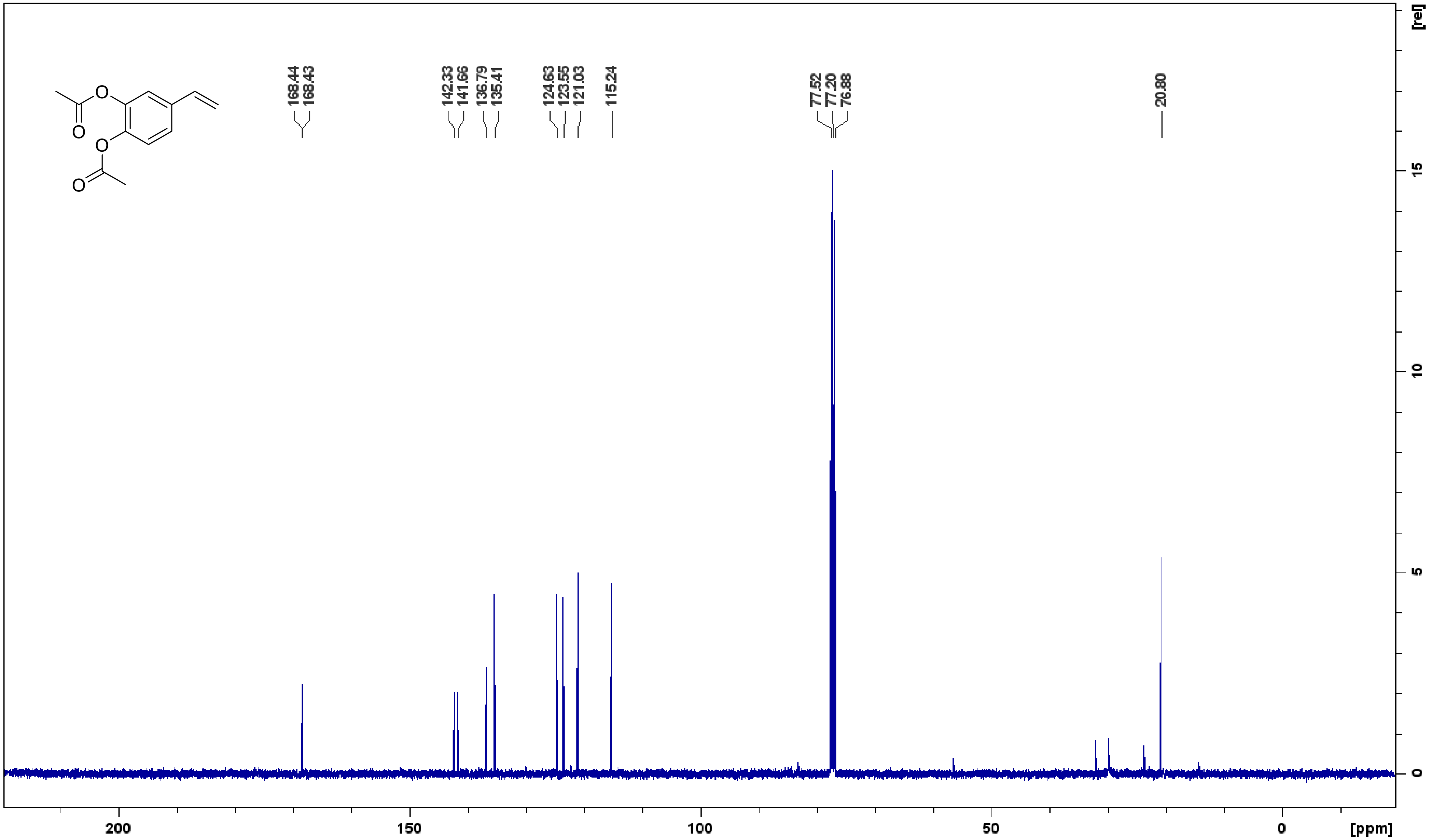

Figure SD 28. ^{13}C-NMR spectrum of 3,4-diacetoxystyrene (DAS) in $CDCl_3$ at 25 °C. impurities: grease, CPME.

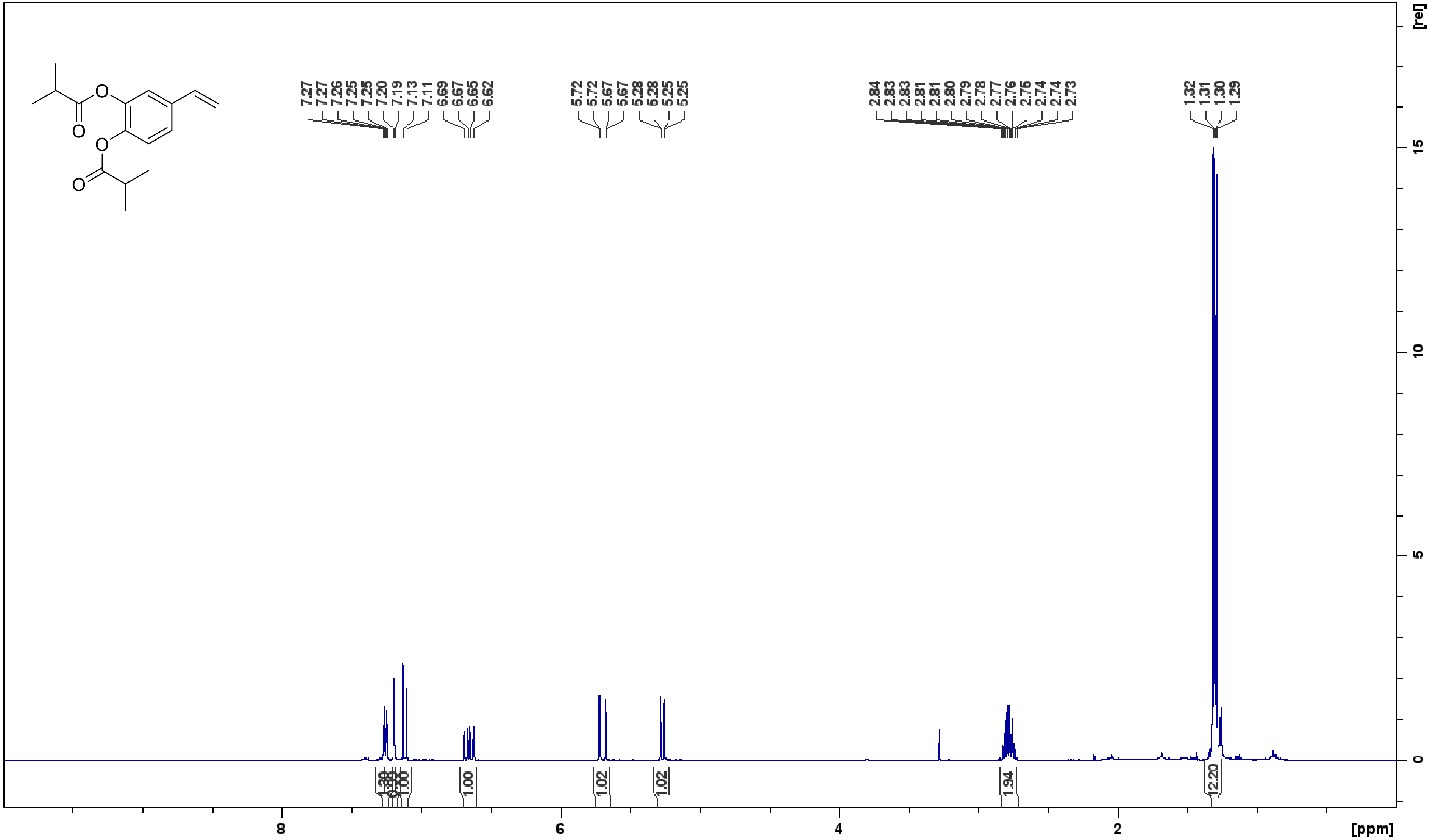

Figure SD 29. ^{1}H-NMR spectrum of 3,4-diisobutanoyloxystyrene (DIBS) in $CDCl_3$ at 25 °C. impurities: grease, CPME.

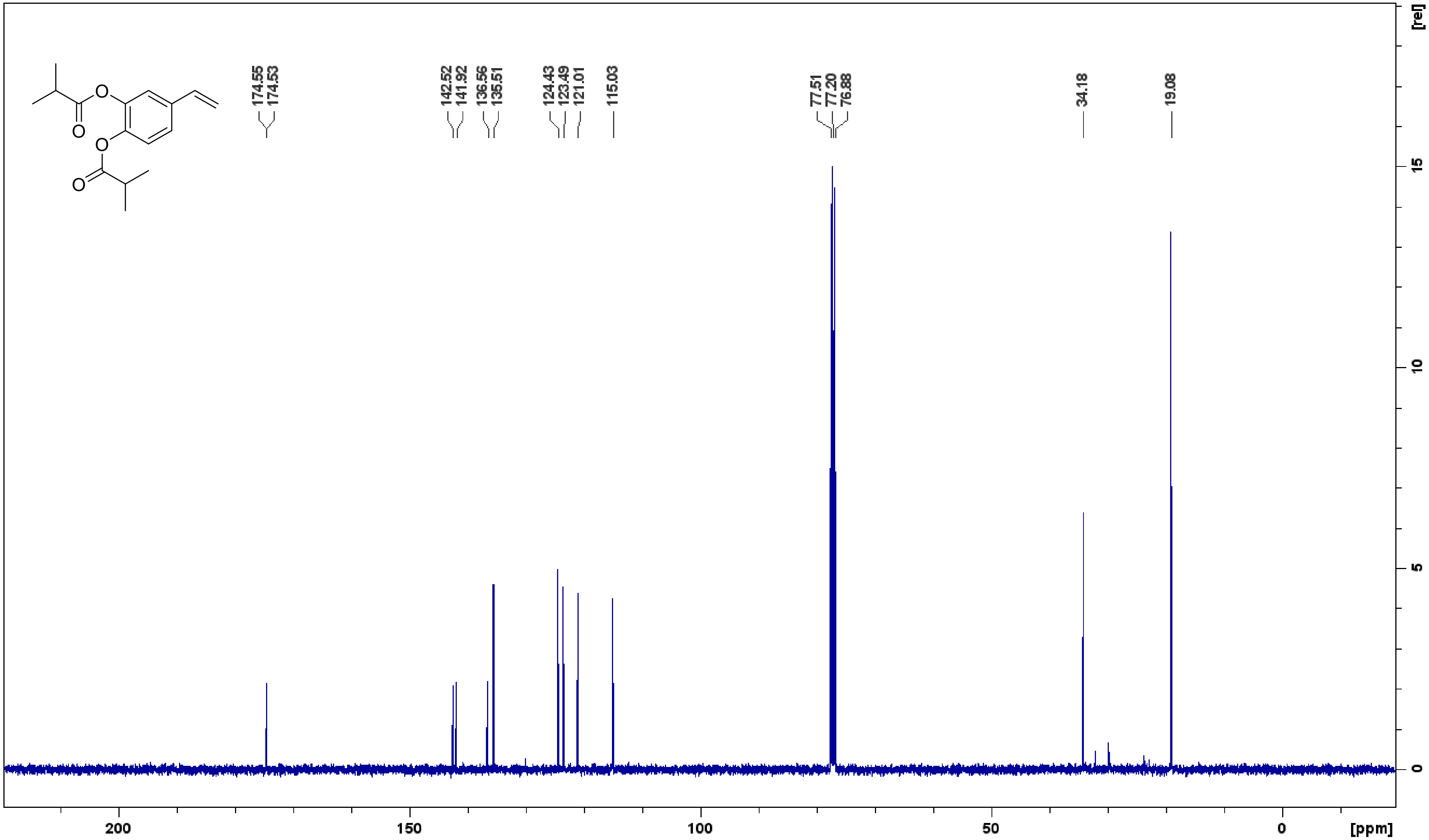

Figure SD 30. ^{13}C-NMR spectrum of 3,4-diisobutanoyloxystyrene (DIBS) in $CDCl_3$ at 25 °C. impurity: grease.

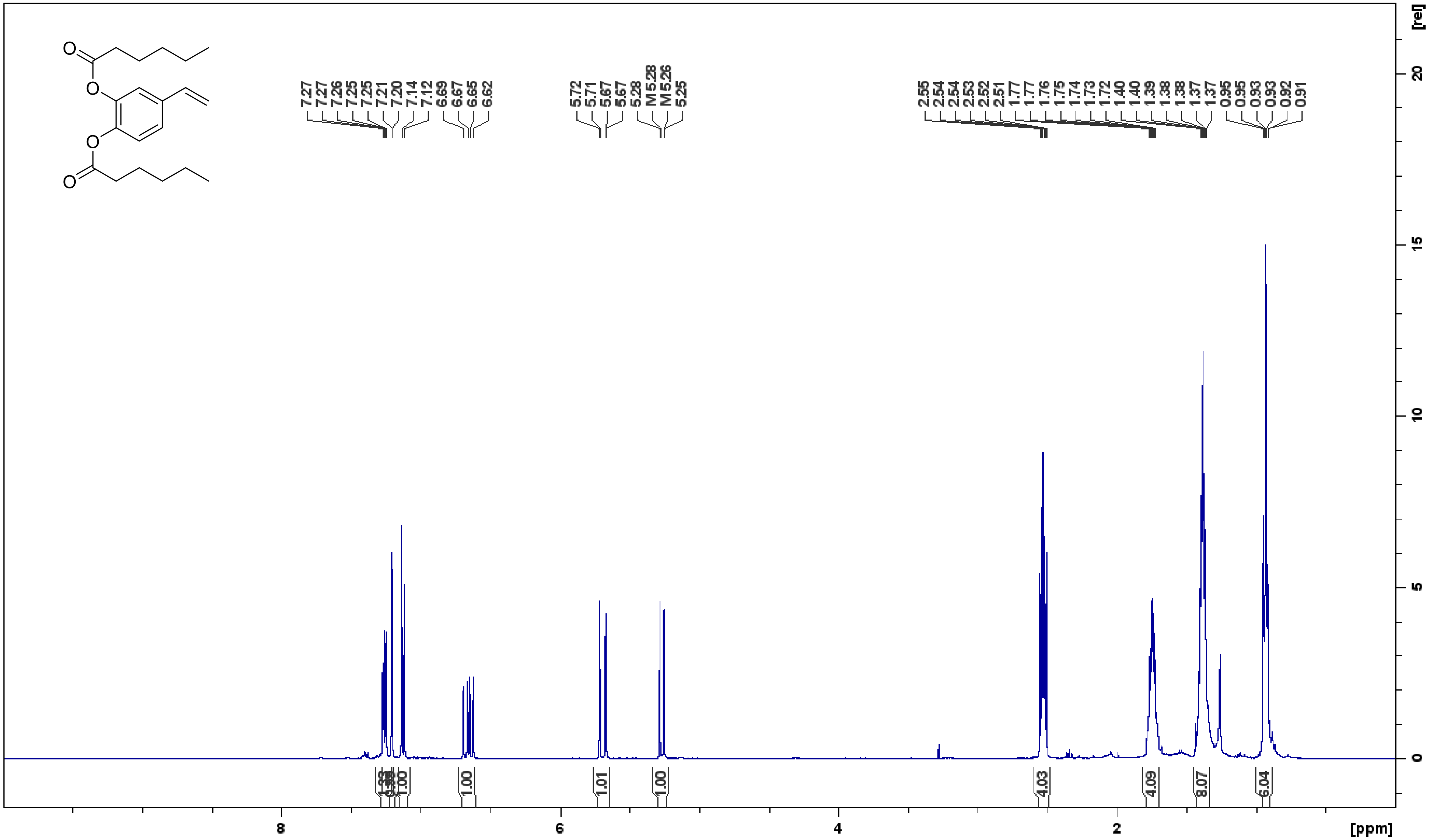

Figure SD 31. ^{1}H-NMR spectrum of 3,4-dihexanoyloxystyrene (DHS) in $CDCl_3$ at 25 °C. impurities: grease, hexanoic acid, CPME.

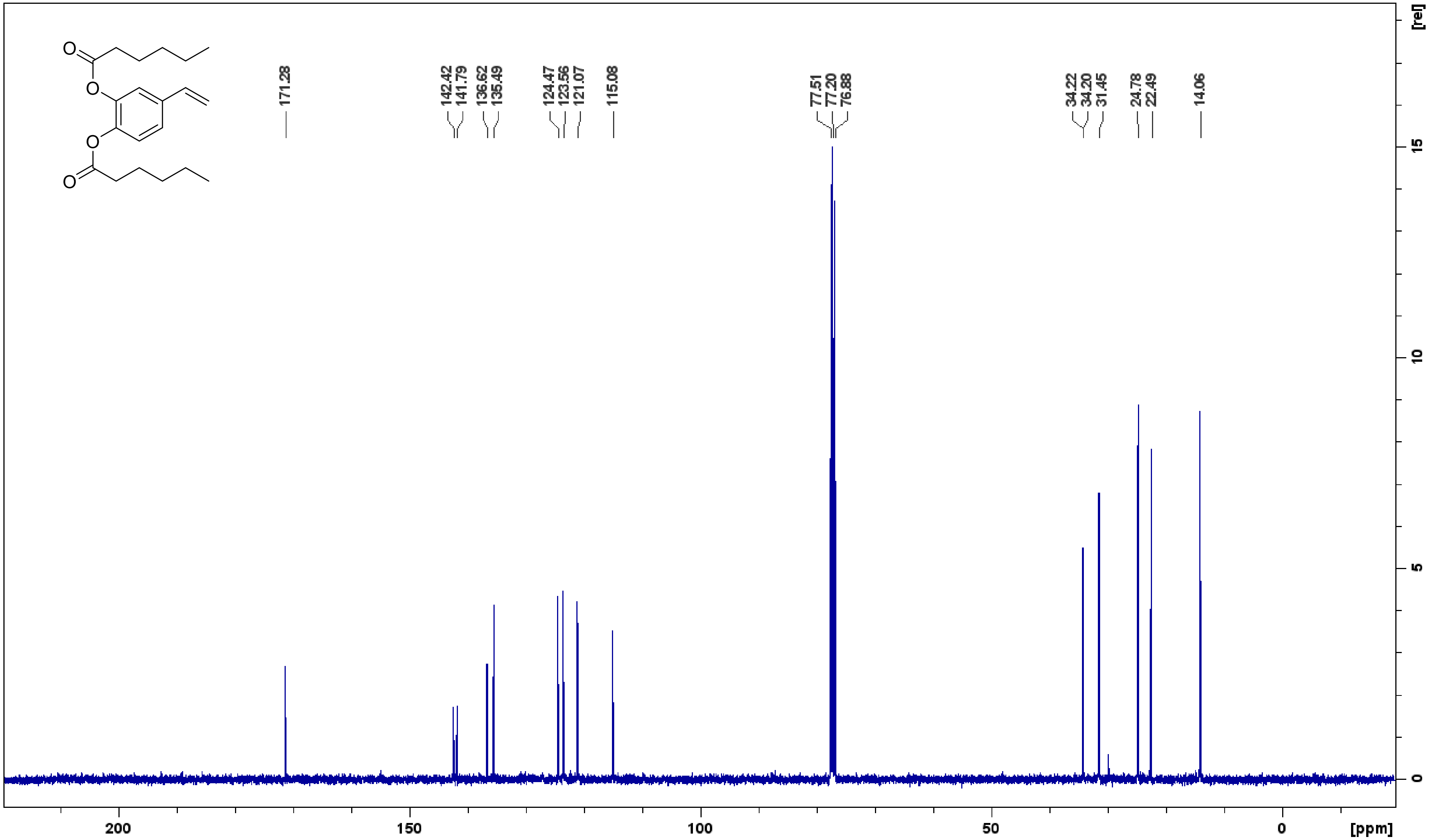

Figure SD 32. ^{13}C-NMR spectrum of 3,4-dihexanoyloxystyrene (DHS) in $CDCl_3$ at 25 °C. impurity: grease.

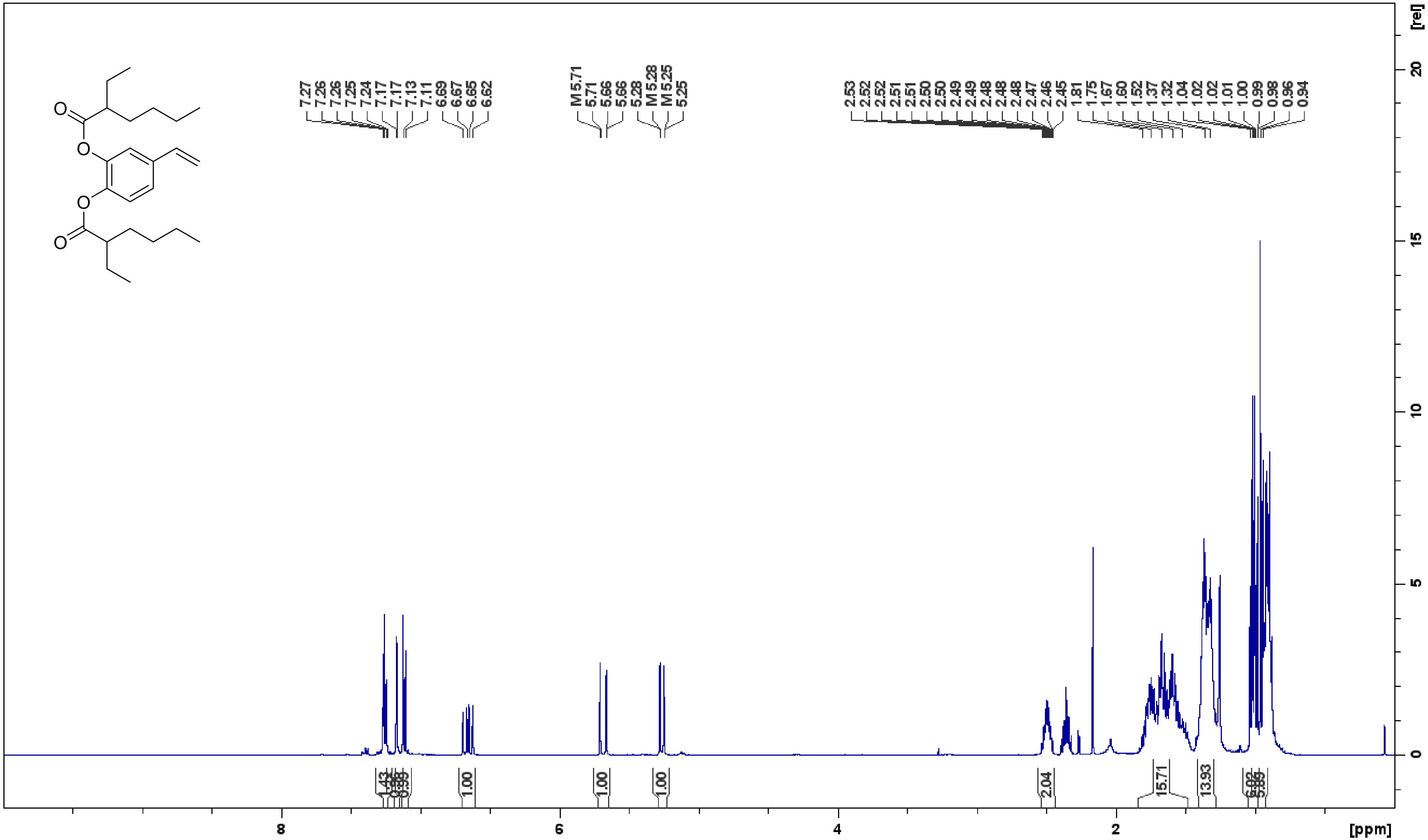

Figure SD 33. ^{1}H-NMR spectrum of 3,4-bis(2-ethyl-hexanoyloxy)styrene (DEHS) in $CDCl_3$ at 25 °C. impurities: 2-ethylhexanoic acid, acetone, grease.

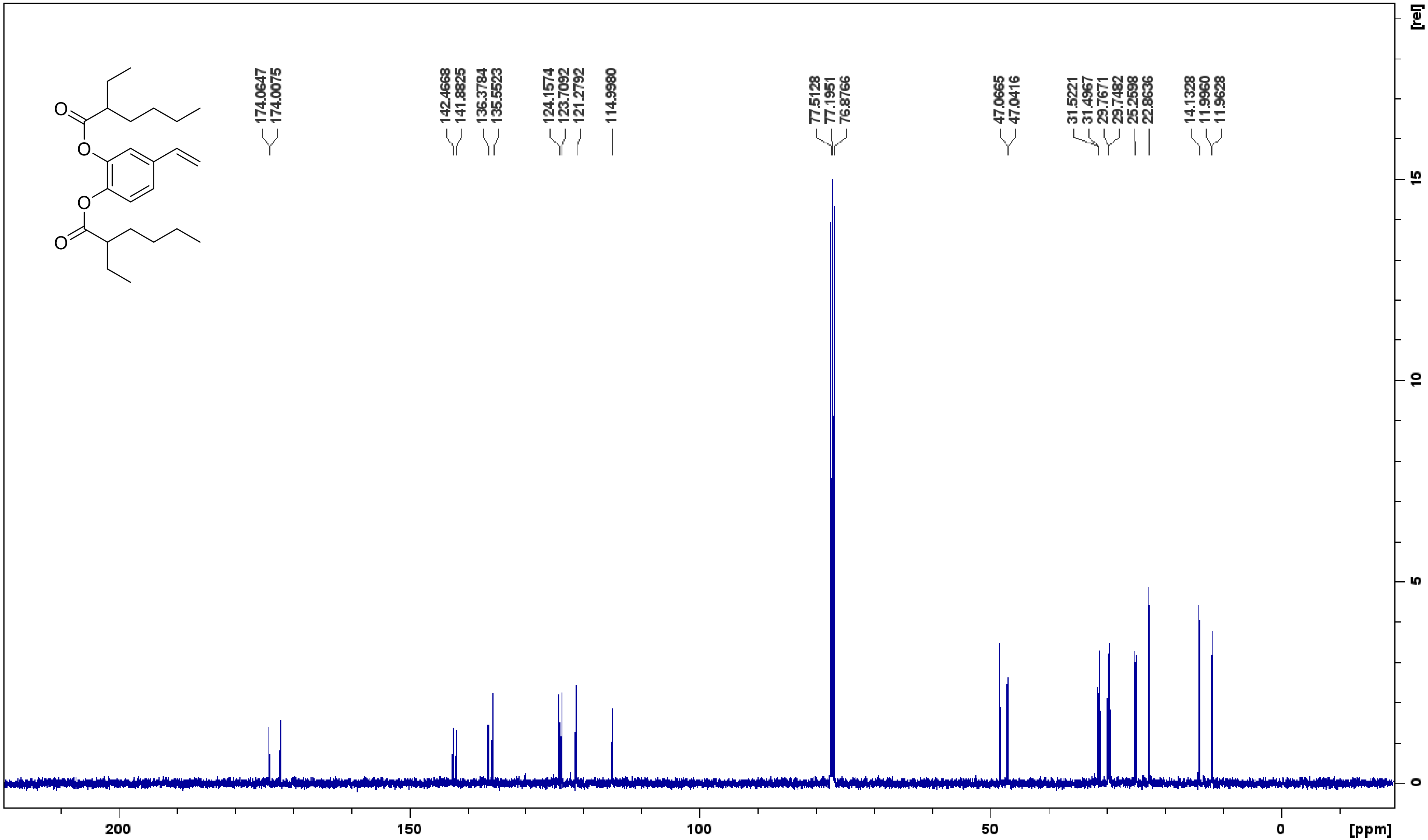

Figure SD 34. ^{13}C-NMR spectrum of 3,4-bis(2-ethyl-hexanoyloxy)styrene (DEHS) in $CDCl_3$ at 25 °C. impurities: 2-ethylhexanoic acid, grease.

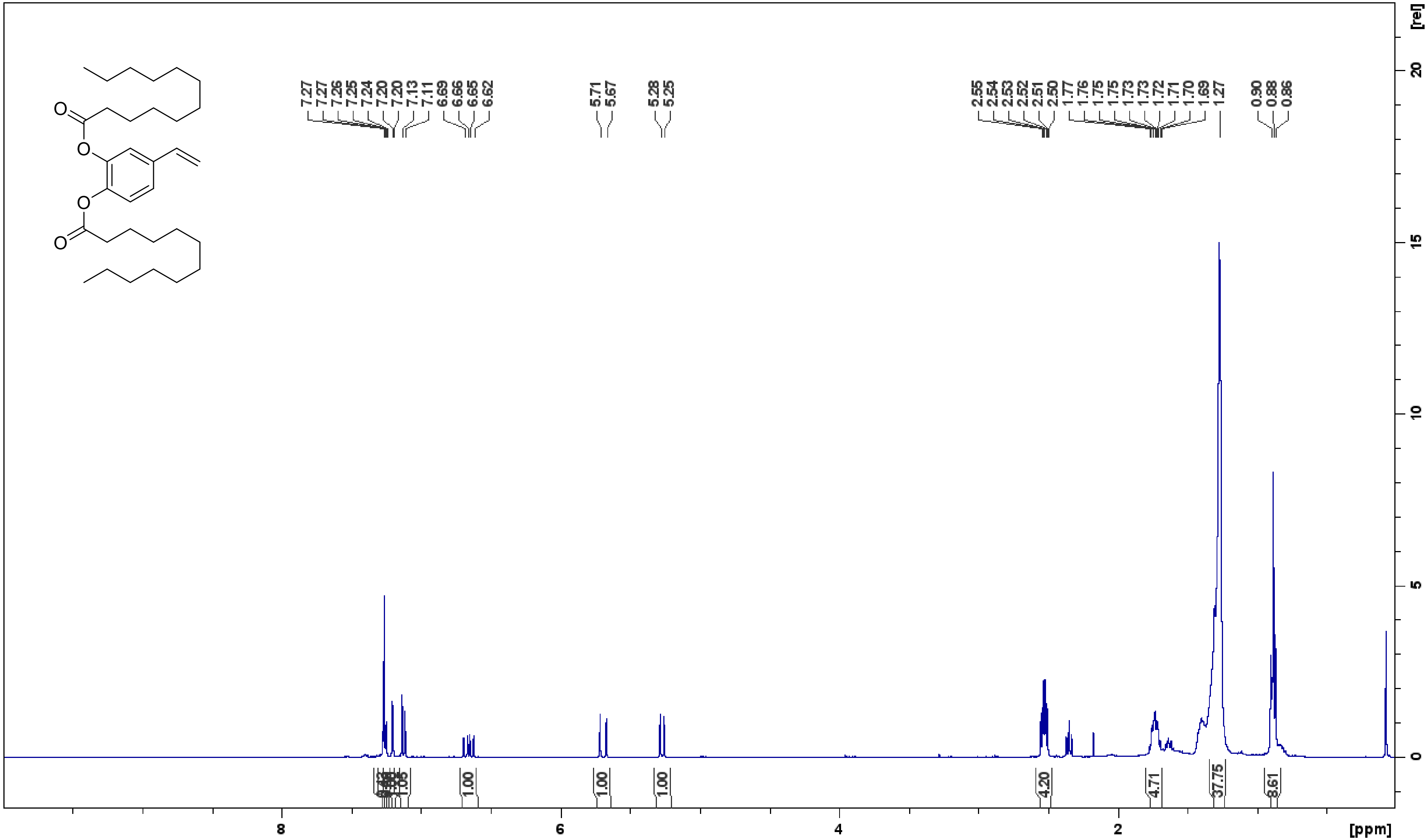

Figure SD 35. ^{1}H-NMR spectrum of 3,4-dilauroyloxystyrene (DLS) in $CDCl_3$ at 25 °C. impurities: lauric acid, acetone, grease.

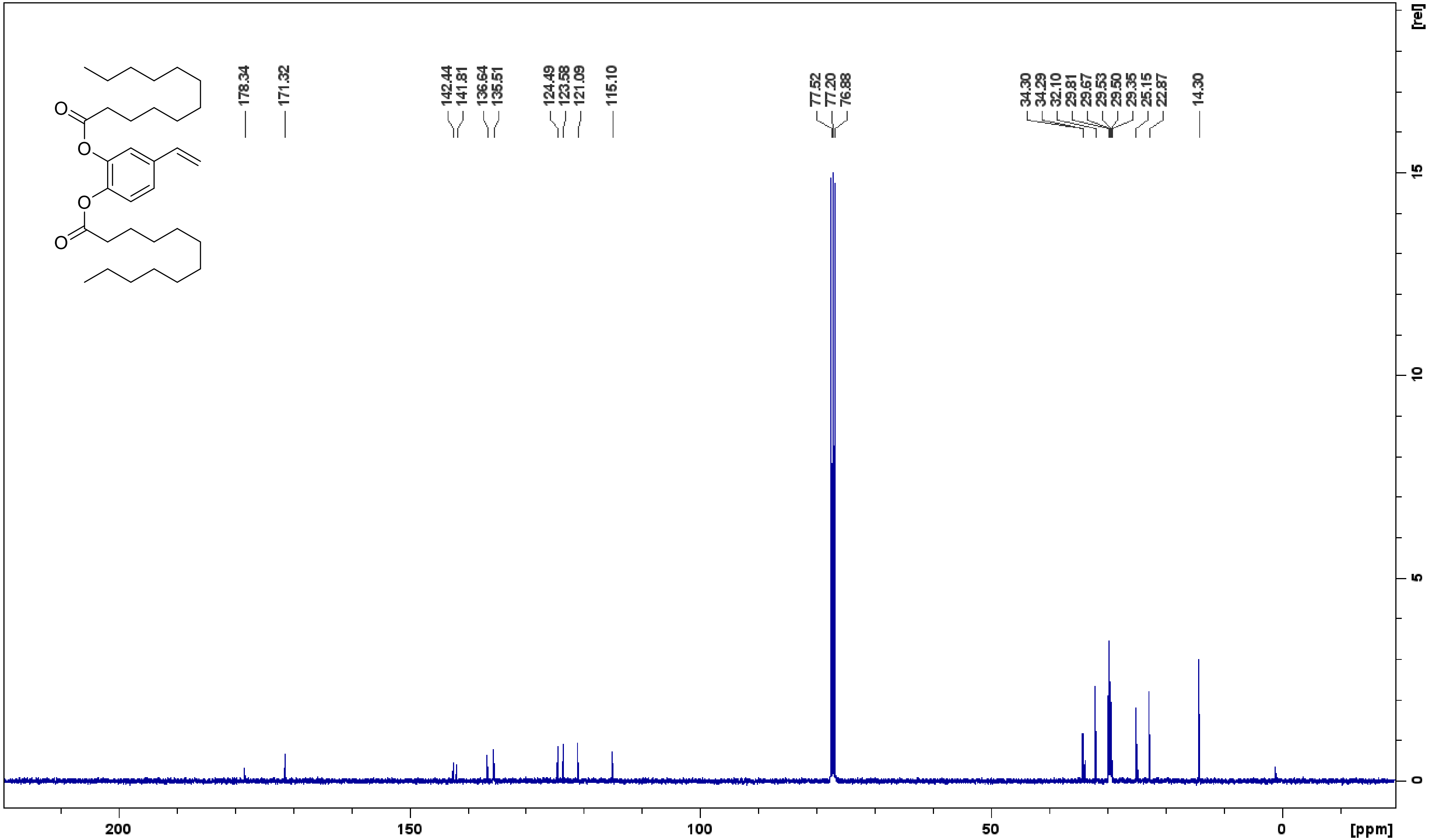

Figure SD 36. ^{13}C-NMR spectrum of 3,4-dilauroyloxystyrene (DLS) in $CDCl_3$ at 25 °C. impurity: grease.

Supplementary References

1. Babij, N. R.; McCusker, E. O.; Whiteker, G. T.; Canturk, B.; Choy, N.; Creemer, L. C.; Amicis, C. V. D.; Hewlett, N. M.; Johnson, P. L.; Knobelsdorf, J. A.; Li, F.; Lorsbach, B. A.; Nugent, B. M.; Ryan, S. J.; Smith, M. R.; Yang, Q., NMR Chemical Shifts of Trace Impurities: Industrially Preferred Solvents Used in Process and Green Chemistry. *Org. Process Res. Dev.* **2016,** *20* (3), 661-667.

2. Gottlieb, H. E.; Kotlyar, V.; Nudelman, A., NMR Chemical Shifts of Common Laboratory Solvents as Trace Impurities. *J. Org. Chem.* **1997,** *62* (21), 7512-7515.
