# Supplementary material for: Integrated preservation of water activity as key to intensified chemoenzymatic synthesis of bio-based styrene derivatives
Source: Commun Chem. 2024 Mar 14;7:57. doi: 10.1038/s42004-024-01138-x (PMC10940287; doi:10.1038/s42004-024-01138-x)
Supplement: Supplementary file 4 — Reporting Summary [file 42004_2024_1138_MOESM4_ESM.pdf]

Corresponding author(s): Selin Kara

Last updated by author(s): Jan 23, 2024

Reporting Summary

Nature Portfolio wishes to improve the reproducibility of the work that we publish. This form provides structure for consistency and transparency in reporting. For further information on Nature Portfolio policies, see our Editorial Policies and the Editorial Policy Checklist.

Statistics

For all statistical analyses, confirm that the following items are present in the figure legend, table legend, main text, or Methods section.

n/a	Confirmed	
☐	☒	The exact sample size (*n*) for each experimental group/condition, given as a discrete number and unit of measurement
☐	☒	A statement on whether measurements were taken from distinct samples or whether the same sample was measured repeatedly
☒	☐	The statistical test(s) used AND whether they are one- or two-sided<br>*Only common tests should be described solely by name; describe more complex techniques in the Methods section.*
☒	☐	A description of all covariates tested
☒	☐	A description of any assumptions or corrections, such as tests of normality and adjustment for multiple comparisons
☐	☒	A full description of the statistical parameters including central tendency (e.g. means) or other basic estimates (e.g. regression coefficient) AND variation (e.g. standard deviation) or associated estimates of uncertainty (e.g. confidence intervals)
☒	☐	For null hypothesis testing, the test statistic (e.g. *F*, *t*, *r*) with confidence intervals, effect sizes, degrees of freedom and *P* value noted<br>*Give P values as exact values whenever suitable.*
☒	☐	For Bayesian analysis, information on the choice of priors and Markov chain Monte Carlo settings
☒	☐	For hierarchical and complex designs, identification of the appropriate level for tests and full reporting of outcomes
☒	☐	Estimates of effect sizes (e.g. Cohen's *d*, Pearson's *r*), indicating how they were calculated

Our web collection on statistics for biologists contains articles on many of the points above.

Software and code

Policy information about availability of computer code

Data collection	Thermodynamic equilibrium calculations were performed using COSMOconf v3.0, Turbomole v6.65, and the BIOVIA COSMOtherm 2020 software environment. For MD simulations, PACKMOL v17.096, the CHARMM-GUI, and GROMACS 2019.4 were used as described in the Methods section of the manuscript.
Data analysis	Data analysis was performed in MS Excel.

For manuscripts utilizing custom algorithms or software that are central to the research but not yet described in published literature, software must be made available to editors and reviewers. We strongly encourage code deposition in a community repository (e.g. GitHub). See the Nature Portfolio guidelines for submitting code & software for further information.

Data

Policy information about availability of data

All manuscripts must include a data availability statement. This statement should provide the following information, where applicable:

- Accession codes, unique identifiers, or web links for publicly available datasets
- A description of any restrictions on data availability
- For clinical datasets or third party data, please ensure that the statement adheres to our policy

Supplementary methods, discussions, and notes are provided in the Supplementary Information, whereas all NMRs are reported as Supplementary Data.

Additionally, the detailed datasets generated during the current study, including both experimental and computational data, are publicly available in the Zenodo open data repository, https://zenodo.org/records/10545172.

Research involving human participants, their data, or biological material

Policy information about studies with human participants or human data. See also policy information about sex, gender (identity/presentation), and sexual orientation and race, ethnicity and racism.

Reporting on sex and gender	This research does not involve human participants, their personal data or biological material.
Reporting on race, ethnicity, or other socially relevant groupings	This research does not involve human participants, their personal data or biological material.
Population characteristics	This research does not involve human participants, their personal data or biological material.
Recruitment	This research does not involve human participants, their personal data or biological material.
Ethics oversight	This research does not involve human participants, their personal data or biological material.

Note that full information on the approval of the study protocol must also be provided in the manuscript.

Field-specific reporting

Please select the one below that is the best fit for your research. If you are not sure, read the appropriate sections before making your selection.

☒ Life sciences ☐ Behavioural & social sciences ☐ Ecological, evolutionary & environmental sciences

For a reference copy of the document with all sections, see nature.com/documents/nr-reporting-summary-flat.pdf

Life sciences study design

All studies must disclose on these points even when the disclosure is negative.

Sample size	No preceding sample size calculations were performed. Experiments were performed in duplicates with high reproducibility.
Data exclusions	No data was excluded.
Replication	The enzymatic activity of the biocatalyst was monitored with our standard activity assay. This revealed months-long shelf-life time of the biocatalyst under chosen storage conditions. Based on this and the established synthetic routine, the biocatalytic decarboxylation itself proved highly reproducible in organic media in close to 100 individual microcentrifuge-scale experiments. The chemocatalytic acylation is even more robust as catalyst deactivation is a non-issue there. All in-house attempts to replicate findings were successful.
Randomization	Guided by the research question, laboratory experiments were drafted, conducted, samples were taken from the reaction mixture and prepared for analysis according to previously established routines. All samples were subjected to HPLC-UV analysis as close in time to sampling as possible without consideration of experimental groups or randomization. Repeated measurements of calibrations and reference standards, ensured the absence of drift caused by the analytical device/detector.
Blinding	Due to operational and safety reasons, the single experimental operator had to be fully aware of reaction conditions of all experiments at all times. This operator set up all experiments, conducted sampling, sample preparation, sample analysis, and data interpretation and was unbiased towards specific outcomes during the experimentations/screenings performed.

Reporting for specific materials, systems and methods

We require information from authors about some types of materials, experimental systems and methods used in many studies. Here, indicate whether each material, system or method listed is relevant to your study. If you are not sure if a list item applies to your research, read the appropriate section before selecting a response.

Materials & experimental systems

n/a	Involved in the study
☒	☐ Antibodies
☒	☐ Eukaryotic cell lines
☒	☐ Palaeontology and archaeology
☒	☐ Animals and other organisms
☒	☐ Clinical data
☒	☐ Dual use research of concern
☒	☐ Plants

Methods

n/a	Involved in the study
☒	☐ ChIP-seq
☒	☐ Flow cytometry
☒	☐ MRI-based neuroimaging

Plants

Seed stocks	No plants used.
Novel plant genotypes	No plants used.
Authentication	No plants used.
